# Continuity of care: time to first outpatient appointment after child and adolescent psychiatric hospital stays in Germany

**DOI:** 10.1186/s12913-026-14322-7

**Published:** 2026-03-13

**Authors:** Stephan Zillmer, Jule Leickert, Annika Vivirito, Dirk Enders, Christoph U. Correll, Charlotte Jaite, Christian J. Bachmann

**Affiliations:** 1 Department of Clinical Psychology and Psychotherapy in Childhood and Adolescence, University of Hildesheim, Hildesheim, Germany; 2 Department of Child and Adolescent Psychiatry, Psychosomatics and Psychotherapy, Charité - Universitaetsmedizin Berlin, Corporate Member of Freie Universitaet Berlin, Humboldt Universitaet zu Berlin, Berlin Institute of Health, Berlin, Germany; 3 Department of Psychiatry and Neurosciences, Campus Charité Mitte (CCM), Charité – Universitätsmedizin Berlin, Corporate Member of Freie Universität Berlin and Humboldt-Universität zu Berlin, Berlin, Germany; 4 Department of Psychiatry, Psychotherapy and Psychosomatics, Vivantes Klinikum am Urban and Vivantes Klinikum im Friedrichshain, Berlin, Germany; 5 InGef - Institute for Applied Health Research Berlin GmbH, Berlin, Germany; 6 Department of Psychiatry and Molecular Medicine, Zucker School of Medicine at Hofstra/Northwell, Hempstead, NY USA; 7 Center for Psychiatric Neuroscience, Feinstein Institute for Medical Research, Manhasset, NY USA; 8 German Center for Mental Health (DZPG), partner site Berlin, Berlin, Germany; 9 German Center for Child and Adolescent Health (DZKJ), partner site Berlin, Berlin, Germany; 10 Einstein Center for Population Diversity (ECPD), Berlin, Germany; 11 Department of Child and Adolescent Psychiatry, Ulm University Medical Center, Ulm, Germany

**Keywords:** Child and adolescent mental health, COVID-19 pandemic, Health care services, Wait times, Anorexia nervosa

## Abstract

**Background:**

Optimizing treatment outcomes requires smooth sectoral transfer, which is especially true in child and adolescent psychiatry where timely outpatient follow-up is crucial. This study measured the time from hospital discharge to first outpatient contact (TDO) within the German healthcare system before and during the COVID-19 pandemic.

**Methods:**

Anonymized data of ~ 700,000 publicly insured children and adolescents (age: 0.0–17.9 years) were retrieved from the InGef database (2018–2021). An exploratory analysis examined TDO among patients with documented outpatient contact following discharge, and length of stay (LOS). Analyses were stratified by index diagnosis, sex, age, residency, socioeconomic status, and outpatient specialty. Results were compared pre-COVID-19 (01/2018–03/2020) vs. intra-COVID-19 (04/2020–12/2021).

**Results:**

Between pre-COVID and intra-COVID, TDO decreased significantly for anorexia nervosa (pediatricians: -22.8 days, |d|=0.28; child and adolescent psychotherapists (CAPPs): -33.4 days, |d|=0.35), anxiety disorders (CAPPs: -27.1 days; |d|=0.22), and depressive disorders (child and adolescent psychiatrists: -25 days, |d|=0.22; CAPPs: -23.6 days, |d|=0.21). Reductions in LOS were significant for depressive disorders (-6.8 days, |d|=0.11). Considering the most rapid mean times to any outpatient provider within diagnoses in the pre-COVID period, depressive disorders (≈ 74 days) and anorexia nervosa (≈ 66 days) showed the longest TDO (anxiety disorders ≈ 47 days; obsessive-compulsive disorders ≈ 61 days; post-traumatic stress disorders ≈ 49 days). Regarding sex, residency and socioeconomic status, only minimal differences were observed.

**Conclusions:**

This study shows delays (> 2 months) between discharge from child and adolescent psychiatric inpatient care and first outpatient contact. Such intervals are noteworthy given the critical need for continuous care in potentially life-threatening conditions like anorexia nervosa, although these intervals shortened during the COVID-19 pandemic. While the underlying mechanisms remain unclear, further investigation is needed to evaluate whether these trends are associated with changes in inter-sectoral transfer.

**Supplementary Information:**

The online version contains supplementary material available at 10.1186/s12913-026-14322-7.

## Introduction

Children and adolescents admitted for psychiatric inpatient care experienced substantial COVID-19 related stress, contributing to their admission and increased symptom severity during treatment [1, 2]. Although numerous studies have examined inpatient versus outpatient care in isolation, little is known about the transfer between these settings for children and adolescents. After discharge, patients are especially vulnerable and face increased risks such as suicidality [3, 4], relapse [5] and readmission [6]. School reintegration is often a particularly socially and emotionally challenging post-discharge adjustment [7, 8]. Accordingly, NICE guidelines recommend a follow-up visit within 7 days of discharge [9], and other studies emphasize the importance of timely follow-up [10, 11].

Alarmingly, about 30% of children and adolescents discharged from psychiatric inpatient care receive no outpatient follow-up within 30 days [12–15]. Aftercare is especially critical for mental disorders such as schizophrenia and mood disorders [10, 15, 16], yet evidence on whether outpatient aftercare reduces readmissions is mixed [17]. This variability likely reflects inconsistent aftercare definitions, differing national contexts, and heterogeneous diagnostic groups [13]. Studies examining the duration until first outpatient contact after discharge and utilization of aftercare are scarce.

Low rates of follow-up treatment are consistent with uneven development and fragmentation of inpatient and outpatient mental health services [18]. In Europe, Germany has the highest density of inpatient psychiatric beds (65 per 100,000 young people^1^), but a relatively low combined number of child and adolescent psychiatrists and psychologists^2^ per inpatient bed (0.63 per bed per 100,000 young people) [19]. As a result, this imbalance between inpatient and outpatient capacity [19, 20] may limit timely and equal regional access and access to low-threshold services [21, 22]. Based on survey data [23] and administrative data [24–26], a recent scoping review estimated that 13–17% of children and adolescents in Germany might require psychiatric or psychotherapeutic treatment by a specialized provider [22]. However, only about 7.3% receive any kind of outpatient psychotherapeutic support [27], and only approximately 1.4% receive guideline-based psychotherapy [28].

Access to psychiatric healthcare for children and adolescents was especially critical during the COVID-19 pandemic, as they were disproportionately affected by lockdown measures and school closures [29]. Numerous studies documented increases in internalizing symptoms [30, 31], anxiety [32] and deterioration in health-related quality of life [33, 34] during the COVID-19 pandemic. There were more pediatric emergency department visits associated with self-harm [35] and rising numbers of female adolescents admitted to hospitals with depressive, anxiety, or eating disorders [36]. Similarly, the proportion of insured children and adolescents with at least one contact with an outpatient child and adolescent psychiatrist/psychotherapist rose between 2019 and 2022 for girls aged 10–14 (+ 7%) and 15–17 (+ 29%) [36]. An increase in utilization during the COVID-19 pandemic was also reported for pediatricians regarding depressive and anxiety disorders [37]. Risk factors for increased pandemic burden among children and adolescents included specific disorders such as eating [38] and depressive disorders [39], female sex [40, 41], older adolescents (12–18 years compared to younger children) [42], rural residence [43], and low socioeconomic status (SES) [42, 44, 45]. In Germany, however, females with higher SES status exhibited increased incidence rates of eating, depressive and anxiety disorders during the COVID-19 pandemic [36], potentially reflecting improved help-seeking or access to care.

An online survey of German outpatient child and adolescent psychotherapists showed that waiting times for an initial consultation doubled during COVID-19 pandemic (from approximately five to ten weeks), while the provision of long-term therapies declined [46]. Additionally, the time from initial request to the start of guideline-based psychotherapy increased by 42% in Germany, from 17.8 weeks in 2018 [47] (Bundespsychotherapeutenkammer [BPtK], 2018) to 25.3 weeks in 2021 [46]. Waiting times may differ when physicians providing child and adolescent psychotherapies are included (initial consultation = 2 weeks in 2016; 3.9 weeks in 2018; start of treatment = 17.9 weeks in 2016, 20.1 weeks in 2018) [48], a group that was not taken into account by Plötner et al. [46]. Data on COVID‑19‑related changes in waiting times for child and adolescent psychiatric and psychotherapeutic outpatient treatment are missing.

Long waiting times for mental health care were already a concern in many countries before the COVID-19 pandemic (in 15 out of 24 OECD countries) [49], and are associated with reduced family engagement in treatment, increased frustration [50], and lower treatment expectations [51]. However, it remains unclear whether longer waiting times also affected access to outpatient follow-up care after inpatient treatment during the COVID-19 pandemic.

To address this gap, this study examined the average time from inpatient discharge to first outpatient contact (TDO) for children and adolescents with psychiatric conditions in Germany. We anticipated that COVID-19 pandemic significantly altered inpatient and outpatient care and therefore analyzed separate pre- and intra-pandemic samples. We also examined the length of inpatient stay (LOS), as this factor may influence both the likelihood and timing of outpatient follow-up [13, 52, 53] as well as the risk of readmission [54, 55]. Previous studies have identified LOS as a predictor of outpatient follow-up within 30 days of discharge [13, 14]. However, the generalizability of these findings is limited, as the cited studies focused either on suicidal children and adolescents (10–18 years) [13] or young adults (18–27 years) [14]. Given that LOS is also predicted by symptom severity [56], a longer LOS is likely associated with increased likelihoods of follow-up and readmission [17]. A Canadian study also found LOS to predict aftercare for children and adolescents and identified additional factors influencing follow-up, including higher income, urban residence, and female sex [12]. Therefore, we stratified our analyses of TDO and LOS by sex, age, SES, region of residence, index diagnosis (depressive disorder, anxiety disorder, obsessive-compulsive disorder [OCD], post-traumatic stress disorder [PTSD], anorexia nervosa), and type of outpatient treatment.

## Methods

### Database and study design

The study used claims data (secondary data) from 4 million people with statutory health insurance from the InGef Research database [57], which is representative of the German population. The study data originated from the same data extraction studies on pandemic-related changes in prevalence and incidence of bulimia nervosa and PTSD by Leickert et al. [58, 59]. In Germany, health insurance is legally required and individuals can choose between private and statutory health insurance, with about 90% of the population being covered by statutory health insurance [60, 61]. Data of female and male children and adolescents between 0.0 and 17.9 years of age (insured for at least one day per quarter) were considered for the periods pre-COVID (01/2018-03/2020) and intra-COVID (04/2020-12/2021). Within our established study population, two independent data extractions were performed. The first extraction identified children and adolescents with ICD-10 diagnoses of depressive disorder (ICD-10: F32-F33, F34.1), anxiety disorder (F40, F41, F93), obsessive-compulsive disorder (F42), or post-traumatic stress disorder (F43.1, F43.8, F43.9) [62] – collectively referred to as the ‘other disorders’ group. The second extraction focused specifically on ‘eating disorders’, identifying children and adolescents with a diagnosis of anorexia nervosa (ICD-10: F50.0, F50.00, F50.01, F50.08, F50.1). For both data extractions, the first diagnosis (= index diagnoses) recorded within the respective study period determined inclusion in the analysis (= prevalent case). The index always refers to the first diagnosis of a patient (patient-level). If there were multiple diagnoses in the same (earliest) quarter, one diagnosis is selected so that each patient is assigned to only one group. Inpatient main and secondary diagnoses and confirmed outpatient diagnoses were used to determine the index diagnosis (and to exclude diagnoses during the study period). The follow-up period was defined as the period from the index quarter to the end of the study period. Analyses were then compiled across all five disorder categories, acknowledging that there was overlap between the ‘other disorders’ and ‘eating disorders’ groups. Based on these prevalent cases, the parameters TDO and LOS were determined as follows: To analyze the TDO (mean duration in days), cases were included that had an outpatient contact (with one of these professional groups: general practitioners, pediatricians, psychiatrists/neurologists, psychological psychotherapists, child and adolescent psychiatrists/child and adolescent psychotherapists) after their first discharge within the study period. Due to small case numbers, results for the professional groups of psychiatrists/neurologists and psychological psychotherapists are presented only in the supplementary tables but were included in the overall analyses (with adjustment for multiple testing). Additionally, only comparisons involving at least 5 cases in both the pre-COVID period and the intra-COVID period were included. This criterion was applied to both variables, TDO and LOS. Outpatient contacts were billed based on the entire catalog of services defined in the German standardized evaluation scale (Einheitlicher Bewertungsmaßstab [EBM]). Only contacts with the specified professional groups were considered. Given that contact was defined using EBM codes, it is not possible to attribute a contact directly to the respective diagnosis and therefore to determine whether it represents continuity of mental health care or general follow-up. The inclusion criterion for the analysis of LOS (mean duration in days) consisted in having at least one inpatient stay (in one of the following departments: child and adolescent psychiatry, general psychiatry, psychosomatics/psychotherapy or pediatrics). Hospital cases with partial or full inpatient status with an admission date between the index quarter and the end of the respective phase were considered. If two hospital cases overlap in the data, they are also counted as two hospitalizations and all days are summed. For supplementary analysis, readmissions are also reported descriptively in the supplement (in one of the following areas: child and adolescent psychiatry, general psychiatry, psychosomatics/psychotherapy, pediatrics, emergency room) that occurred between inpatient discharge and the first outpatient contact in the respective study phase. Because of the anonymization of the insurance data in the InGef database, no ethics committee approval and subject consent was needed for this study. No individuals, statutory health insurance companies, or health care providers can be identified in the data.

### Statistical analysis

The data were stratified and analyzed separately by sex (female, male), age (0–9 years, 10–13 years, 14–17 years), region of residence (urban, rural, unknown), SES (low SES, middle SES, high SES, unknown) and diagnosis (depressive disorder, anxiety disorder, obsessive-compulsive disorder, post-traumatic stress disorder, anorexia nervosa). While included in the overall sample, cases with unknown region of residence and SES were not analyzed as distinct groups. The SES was based on the German Index of Socioeconomic Deprivation (high index value = lower-status) [63]. Due to the use of secondary data, all analyses are based on aggregated data, and no individual-level analyses were performed. The pre-COVID and intra-COVID periods were compared using descriptive statistics (difference in mean duration of days), Welch tests, and Cohen’s d as an estimate of the effect size (small effect: ≥0.2, medium effect: ≥0.5, large effect: ≥0.8) [64]. The analyses were carried out using R Statistical Software (R versions 4.0.2) and Microsoft Excel for Mac version 16.94. The tables were created using the rempsyc package [65]. While the overall study design was exploratory, our primary analysis focused on comparing TDO between pre-and intra-COVID periods, stratified by index diagnoses and type of treatment. Because of the large variations in group sizes among sex, age, SES, and disorder categories, significance tests were used primarily to explore the most relevant results. Given the exploratory nature of the statistical approach and the large number of tests performed, we applied a False Discovery Rate (FDR) correction using the Benjamini-Hochberg procedure [66] to minimize the risk of false-positive findings. The FDR was set at 5%.

## Results

The overall database included for the pre-COVID period 01/2018-03/2020 was 710,629 children and adolescents, and for the intra-COVID period 04/2020-12/2021 was 698,108 children and adolescents that were representative of the German children and adolescents with statutory health insurance.

### Duration until first outpatient contact after inpatient discharge stratified by disorder, age and sex

**Fig. 1:**
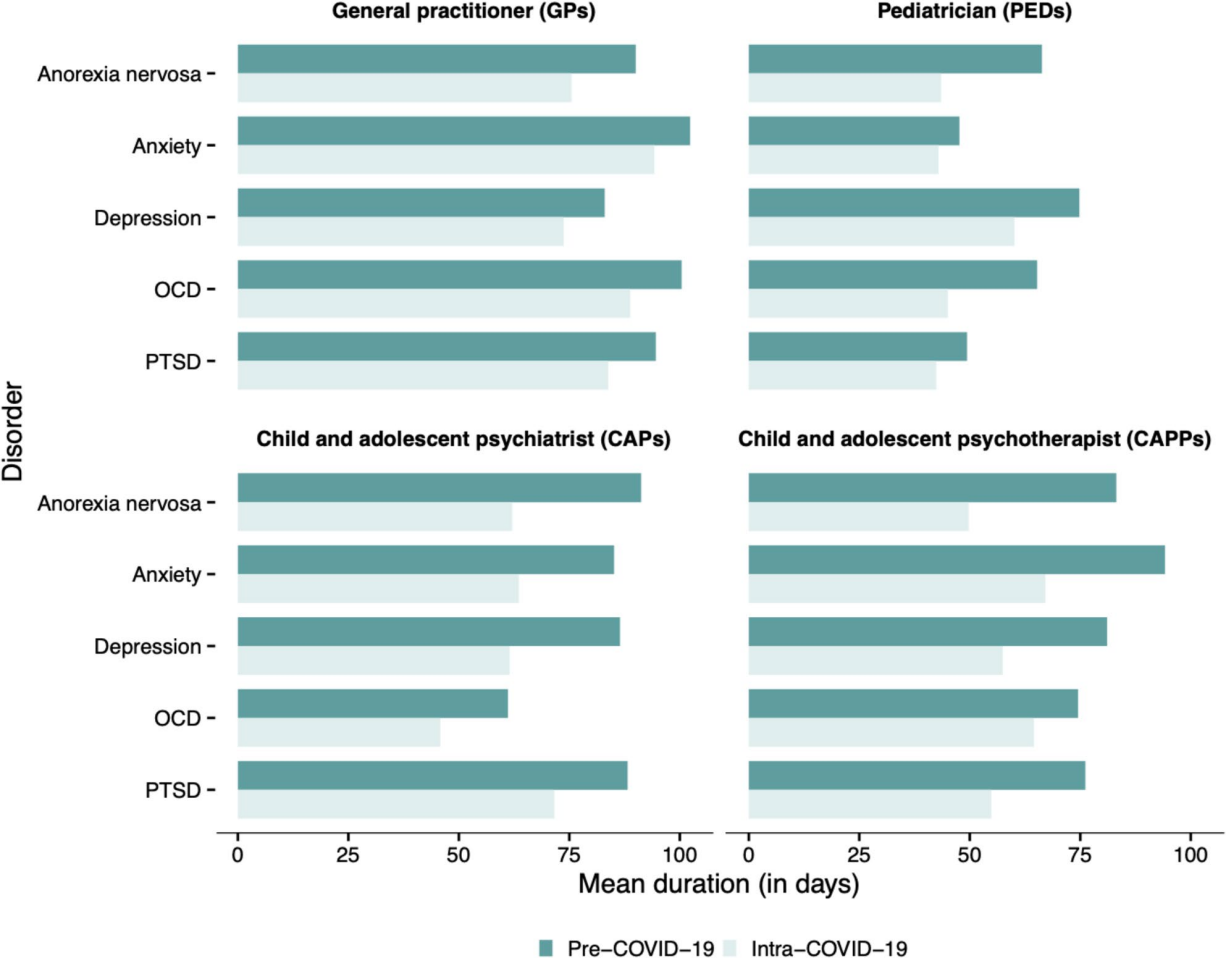
Average time to first outpatient contact after discharge (TDO) by disorder and professional group

Based on disorder-specific comparisons, reductions in TDO were observed across age and sex groups from pre-COVID to intra-COVID: After FDR correction, significant reductions (and at least Cohen’s d > 0.2) in TDO were observed for depressive disorders (child and adolescent psychiatrists (CAPs): -25 days, d = 0.22, *p*<.001; child and adolescent psychotherapists (CAPPs): -23.6 days, d = 0.21, *p*<.001; for an overview, see also Table 1a; Fig. 1), anxiety disorders (CAPPs: -27.1 days, d = 0.22, *p*<.001), and anorexia nervosa (CAPPs: -33.4 days, d = 0.35, *p*=.009; pediatricians (PEDs): -22.8 days, d = 0.28, *p*=.037). These reductions were consistently more pronounced in psychiatry-specific professional groups (CAPs/CAPPs) compared to non-psychiatry-professional groups (general practitioners (GPs)/PEDs). No significant changes in TDO were observed for OCD and PTSD. For a detailed overview of all TDO means between pre-COVID and intra-COVID, as well as the total number of contacts per professional group, see Table 1a, 1b and 1c.

TDO significantly decreased within specific age groups. In the 10–13 age group, reductions were significant for anxiety disorders (CAPs: -26.6 days, d = 0.24, *p*=.005; CAPPs: -28.9 days, d = 0.23, *p*=.009) and depressive disorders (CAPs: -36.9 days, d = 0.30, *p*=.004; CAPPs: -25.9 days, d = 0.23, *p*=.027). The 14–17 age group showed significant reductions for anorexia nervosa (CAPPs: -38.8 days, d = 0.39, *p*=.016; PEDs: -31.0, d = 0.36, *p*=.044). Finally, in the 0–9 age group, there were no significant changes between pre-COVID and intra-COVID.

Examining differences by sex, significant reductions in TDO were observed in female children and adolescents for anxiety disorders (CAPPs: -29.3 days, d = 0.24, *p*=.001) and anorexia nervosa (CAPPs: -32.4 days, d = 0.32, *p*=.023; PEDs: -27.2 days, d = 0.30, *p*=.036), and in male children and adolescents for depressive disorders (CAPs: -39.8 days, d = 0.34, *p*=.002; CAPPs: -41.5 days, d = 0.35, *p*=.002). Within the subgroupings of age or sex, the observed effects were limited to or more pronounced within psychiatry-specific specialist groups.

**Table 1a:** Duration until first outpatient contact after inpatient discharge (TDO) of children and adolescents with psychiatric disorders before vs. during the COVID-19 pandemic, by disorder and professional group

ICD-10				Pre-COVID		Intra-COVID						
	Professional group	*n* 2019	*n* 2021	M	SD	M	SD	ΔM	ΔM [95%]	*p*	*p*.adj	|d|
Anorexia nervosa	General practitioner	291	259	90.02	131.20	75.53	97.50	-14.5	[-34.01, 5.02]	0.139	0.347	0.12
	Pediatrician	212	245	66.33	100.48	43.49	61.53	-22.8	[-37.89, -7.77]	0.004	**0.037**	**0.28**
	Child and adolescent psychiatrist	157	166	91.25	123.23	62.07	88.78	-29.2	[-52.51, -5.85]	0.016	0.085	0.27
	Child and adolescent psychotherapist	202	241	83.16	116.63	49.76	71.74	-33.4	[-51.14, -15.66]	< 0.001	**0.009**	**0.35**
Anxiety disorder	General practitioner	2467	1514	102.34	132.72	94.25	114.28	-8.1	[-16.15, -0.02]	0.042	0.150	0.06
	Pediatrician	3858	2595	47.66	80.73	42.92	67.04	-4.7	[-8.50, -0.98]	0.010	0.064	0.06
	Child and adolescent psychiatrist	1395	975	85.13	124.06	63.57	92.99	-21.6	[-30.75, -12.37]	< 0.001	< 0.001	0.19
	Child and adolescent psychotherapist	1246	911	94.18	140.43	67.10	98.67	-27.1	[-37.72, -16.44]	< 0.001	**< 0.001**	**0.22**
Depressive disorder	General practitioner	2434	1628	83.00	121.48	73.70	100.06	-9.3	[-16.42, -2.19]	0.008	0.052	0.08
	Pediatrician	1407	1182	74.80	107.62	60.10	80.70	-14.7	[-22.15, -7.26]	< 0.001	0.003	0.15
	Child and adolescent psychiatrist	1129	956	86.46	128.99	61.47	91.89	-25.0	[-34.77, -15.22]	< 0.001	**< 0.001**	**0.22**
	Child and adolescent psychotherapist	1203	1042	81.08	127.56	57.47	86.01	-23.6	[-32.76, -14.47]	< 0.001	**< 0.001**	**0.21**
OCD	General practitioner	97	71	100.41	143.81	88.77	109.41	-11.6	[-51.56, 28.29]	0.552	0.728	0.09
	Pediatrician	90	83	65.24	89.71	45.05	54.68	-20.2	[-42.56, 2.17]	0.073	0.222	0.27
	Child and adolescent psychiatrist	59	48	61.12	94.12	45.81	63.50	-15.3	[-46.49, 15.87]	0.320	0.536	0.19
	Child and adolescent psychotherapist	66	54	74.50	121.55	64.52	81.78	-10.0	[-47.95, 27.98]	0.593	0.751	0.09
PTSD	General practitioner	384	242	94.57	127.05	83.79	118.33	-10.8	[-30.69, 9.13]	0.281	0.510	0.09
	Pediatrician	464	275	49.38	83.50	42.41	63.80	-7.0	[-18.42, 4.48]	0.202	0.431	0.09
	Child and adolescent psychiatrist	134	101	88.19	127.81	71.60	99.63	-16.6	[-46.69, 13.52]	0.265	0.496	0.14
	Child and adolescent psychotherapist	183	110	76.15	119.34	54.86	79.05	-21.3	[-46.37, 3.79]	0.068	0.211	0.20

Note. Cohen’s d: small effect: ≥0.2, medium effect: ≥0.5, large effect: ≥0.8; n, sample size; M, mean (in days); SD, standard deviation; ΔM, mean difference (intra-COVID-19 – pre-COVID-19); only comparisons with *n* > 5 are included and only comparisons with *p.adj* < 0.05 and |d| ≥ 0.2 are highlighted

**Table 1b:** Duration until first outpatient contact after inpatient discharge (TDO) of children and adolescents with psychiatric disorders before vs. during the COVID-19 pandemic, by age

ICD-10				Pre-COVID		Intra-COVID						
	Professional group	*n* 2019	*n* 2021	M	SD	M	SD	ΔM	ΔM [95%]	*p*	*p*.adj	|d|
0–9 yrs.												
Anorexia nervosa	General practitioner	< 5	< 5	-	-	-	-	-	-	-	-	-
	Pediatrician	5	8	45.60	91.91	9.88	7.57	-35.7	[-98.02, 26.57]	0.435	0.653	0.64
	Child and adolescent psychiatrist	< 5	< 5	-	-	-	-	-	-	-	-	-
	Child and adolescent psychotherapist	< 5	< 5	-	-	-	-	-	-	-	-	-
Anxiety disorder	General practitioner	748	374	121.47	146.82	103.45	111.48	-18.0	[-34.90, -1.12]	0.022	0.102	0.13
	Pediatrician	2156	1240	39.97	68.15	34.50	55.49	-5.5	[-9.92, -1.00]	0.011	0.066	0.09
	Child and adolescent psychiatrist	533	300	83.69	123.59	66.93	101.08	-16.8	[-33.16, -0.34]	0.035	0.137	0.14
	Child and adolescent psychotherapist	381	220	98.29	145.71	71.34	101.18	-26.9	[-48.72, -5.17]	0.008	0.052	0.21
Depressive disorder	General practitioner	58	17	97.93	141.10	46.88	38.33	-51.0	[-119.14, 17.04]	0.016	0.086	0.41
	Pediatrician	102	55	68.52	95.31	59.53	75.96	-9.0	[-38.19, 20.21]	0.520	0.709	0.10
	Child and adolescent psychiatrist	38	15	92.39	120.45	79.13	110.74	-13.3	[-83.71, 57.18]	0.705	0.812	0.11
	Child and adolescent psychotherapist	23	13	134.39	187.48	39.92	48.16	-94.5	[-198.86, 9.93]	0.031	0.126	0.62
OCD	General practitioner	10	9	123.80	166.14	118.33	130.98	-5.5	[-141.11, 130.18]	0.937	0.960	0.04
	Pediatrician	24	27	75.71	66.12	35.85	40.37	-39.9	[-69.55, -10.16]	0.015	0.082	0.74
	Child and adolescent psychiatrist	< 5	< 5	-	-	-	-	-	-	-	-	-
	Child and adolescent psychotherapist	< 5	< 5	-	-	-	-	-	-	-	-	-
PTSD	General practitioner	86	37	107.01	142.53	141.32	148.91	34.3	[-21.35, 89.98]	0.240	0.473	0.24
	Pediatrician	236	119	35.00	55.02	31.87	51.16	-3.1	[-14.97, 8.72]	0.597	0.753	0.06
	Child and adolescent psychiatrist	25	22	77.60	110.40	63.68	100.22	-13.9	[-74.52, 46.68]	0.653	0.778	0.13
	Child and adolescent psychotherapist	52	20	70.33	130.49	66.75	67.52	-3.6	[-63.81, 56.66]	0.880	0.924	0.03
10–13 yrs.												
Anorexia nervosa	General practitioner	60	62	76.20	102.57	68.47	89.63	-7.7	[-41.88, 26.42]	0.659	0.782	0.08
	Pediatrician	86	105	55.77	89.66	45.88	64.84	-9.9	[-31.84, 12.06]	0.393	0.610	0.13
	Child and adolescent psychiatrist	42	50	91.79	122.70	52.78	77.66	-39.0	[-80.32, 2.31]	0.079	0.234	0.39
	Child and adolescent psychotherapist	58	79	84.45	100.21	59.42	75.55	-25.0	[-54.45, 4.39]	0.113	0.300	0.29
Anxiety disorder	General practitioner	810	454	109.69	135.09	98.48	115.89	-11.2	[-25.98, 3.56]	0.121	0.312	0.09
	Pediatrician	1127	818	51.61	83.76	46.95	67.46	-4.7	[-11.62, 2.30]	0.175	0.397	0.06
	Child and adolescent psychiatrist	534	355	85.67	125.06	59.08	82.91	-26.6	[-41.38, -11.80]	< 0.001	**0.005**	**0.24**
	Child and adolescent psychotherapist	479	360	95.33	139.02	66.39	100.34	-28.9	[-45.88, -12.00]	< 0.001	**0.009**	**0.23**
Depressive disorder	General practitioner	518	336	95.20	125.35	84.14	106.87	-11.1	[-27.32, 5.20]	0.168	0.389	0.09
	Pediatrician	523	431	72.19	101.92	56.32	73.21	-15.9	[-27.35, -4.38]	0.005	0.042	0.18
	Child and adolescent psychiatrist	333	258	96.38	143.52	59.47	86.13	-36.9	[-56.72, -17.11]	< 0.001	**0.004**	**0.30**
	Child and adolescent psychotherapist	345	301	85.81	133.32	59.93	81.85	-25.9	[-43.24, -8.52]	0.003	**0.027**	**0.23**
OCD	General practitioner	35	9	71.03	91.23	61.22	52.44	-9.8	[-72.23, 52.62]	0.678	0.790	0.12
	Pediatrician	37	20	35.08	67.44	52.05	53.30	17.0	[-17.25, 51.19]	0.303	0.521	0.27
	Child and adolescent psychiatrist	29	11	52.69	107.02	38.09	43.02	-14.6	[-80.17, 50.97]	0.542	0.720	0.15
	Child and adolescent psychotherapist	34	13	81.03	127.34	65.85	85.47	-15.2	[-90.37, 60.00]	0.641	0.777	0.13
PTSD	General practitioner	106	60	113.70	137.98	114.57	135.91	0.9	[-42.59, 44.33]	0.969	0.979	0.01
	Pediatrician	134	81	56.73	78.91	46.99	70.75	-9.7	[-30.69, 11.21]	0.350	0.561	0.13
	Child and adolescent psychiatrist	51	29	96.31	147.38	99.62	120.46	3.3	[-59.75, 66.36]	0.914	0.942	0.02
	Child and adolescent psychotherapist	59	35	62.83	94.57	47.03	64.42	-15.8	[-51.22, 19.61]	0.339	0.551	0.19
14–17 yrs.												
Anorexia nervosa	General practitioner	227	196	93.34	137.91	78.13	100.07	-15.2	[-38.50, 8.07]	0.191	0.413	0.12
	Pediatrician	121	132	74.69	107.75	43.64	60.31	-31.0	[-52.34, -9.76]	0.006	**0.044**	**0.36**
	Child and adolescent psychiatrist	114	115	91.44	124.43	66.57	93.46	-24.9	[-53.36, 3.61]	0.089	0.257	0.23
	Child and adolescent psychotherapist	143	158	83.16	123.23	44.37	69.93	-38.8	[-61.16, -16.42]	0.001	**0.016**	**0.39**
Anxiety disorder	General practitioner	909	686	80.04	113.99	86.43	114.36	6.4	[-4.93, 17.70]	0.269	0.496	0.06
	Pediatrician	575	537	68.77	109.06	56.23	85.61	-12.5	[-24.12, -0.96]	0.033	0.131	0.13
	Child and adolescent psychiatrist	328	320	86.59	123.53	65.40	95.72	-21.2	[-38.23, -4.14]	0.015	0.082	0.19
	Child and adolescent psychotherapist	386	331	88.70	137.01	65.05	95.31	-23.6	[-41.21, -6.09]	0.007	0.048	0.20
Depressive disorder	General practitioner	1858	1275	79.13	119.53	71.31	98.59	-7.8	[-15.77, 0.12]	0.046	0.161	0.07
	Pediatrician	782	696	77.37	112.77	62.49	85.36	-14.9	[-25.18, -4.59]	0.004	0.036	0.15
	Child and adolescent psychiatrist	758	683	81.81	122.41	61.84	93.64	-20.0	[-31.31, -8.62]	< 0.001	0.009	0.18
	Child and adolescent psychotherapist	835	728	77.66	122.85	56.76	88.21	-20.9	[-31.64, -10.15]	< 0.001	0.004	0.19
OCD	General practitioner	52	53	115.69	166.05	88.43	113.00	-27.3	[-81.50, 26.98]	0.329	0.544	0.19
	Pediatrician	29	36	95.07	117.99	48.06	64.43	-47.0	[-92.09, -1.94]	0.061	0.197	0.51
	Child and adolescent psychiatrist	28	34	72.61	82.61	52.00	70.44	-20.6	[-58.70, 17.49]	0.301	0.521	0.27
	Child and adolescent psychotherapist	28	37	65.50	121.70	56.57	78.26	-8.9	[-57.65, 39.79]	0.736	0.829	0.09
PTSD	General practitioner	192	145	78.44	111.03	56.38	91.21	-22.1	[-44.27, 0.15]	0.046	0.163	0.21
	Pediatrician	94	75	75.01	129.95	54.17	71.72	-20.8	[-53.64, 11.96]	0.188	0.410	0.19
	Child and adolescent psychiatrist	58	50	85.60	117.55	58.84	83.69	-26.8	[-65.83, 12.30]	0.172	0.393	0.26
	Child and adolescent psychotherapist	72	55	91.28	128.71	55.53	91.13	-35.8	[-75.77, 4.27]	0.069	0.215	0.31

Note. Cohen’s d: small effect: ≥0.2, medium effect: ≥0.5, large effect: ≥0.8; n, sample size; M, mean (in days); SD, standard deviation; ΔM, mean difference (intra-COVID-19 – pre-COVID-19); only comparisons with *n* > 5 are included and only comparisons with *p.adj* < 0.05 and |d| ≥ 0.2 are highlighted

**Table 1c:** Duration until first outpatient contact after inpatient discharge (TDO) of children and adolescents with psychiatric disorders before vs. during the COVID-19 pandemic, by sex

ICD-10				Pre-COVID		Intra-COVID						
	Professional group	*n* 2019	*n* 2021	M	SD	M	SD	ΔM	ΔM [95%]	*p*	*p*.adj	|d|
Female children and adolescents												
Anorexia nervosa	General practitioner	276	186	90.35	132.13	76.60	98.63	-13.7	[-36.02, 8.52]	0.202	0.431	0.11
	Pediatrician	193	121	70.61	103.66	43.39	62.16	-27.2	[-47.67, -6.76]	0.004	**0.036**	**0.30**
	Child and adolescent psychiatrist	148	106	90.49	124.64	59.68	85.16	-30.8	[-58.23, -3.40]	0.020	0.097	0.28
	Child and adolescent psychotherapist	193	150	83.30	118.17	50.87	72.63	-32.4	[-53.94, -10.92]	0.002	**0.023**	**0.32**
Anxiety disorder	General practitioner	1342	848	95.90	129.51	85.60	105.66	-10.3	[-20.70, 0.08]	0.042	0.150	0.09
	Pediatrician	1813	1253	52.05	86.71	43.14	69.60	-8.9	[-14.68, -3.14]	0.002	0.021	0.11
	Child and adolescent psychiatrist	653	510	82.93	122.56	61.76	87.69	-21.2	[-33.75, -8.58]	0.001	0.012	0.19
	Child and adolescent psychotherapist	714	558	91.12	140.89	61.81	96.39	-29.3	[-42.97, -15.64]	< 0.001	**0.001**	**0.24**
Depressive disorder	General practitioner	1711	1240	82.33	122.08	73.83	99.32	-8.5	[-16.76, -0.23]	0.037	0.143	0.08
	Pediatrician	964	901	76.98	108.40	60.13	82.04	-16.9	[-25.62, -8.08]	< 0.001	0.005	0.17
	Child and adolescent psychiatrist	822	758	85.11	126.81	64.38	95.16	-20.7	[-31.85, -9.59]	< 0.001	0.006	0.18
	Child and adolescent psychotherapist	923	852	78.15	124.06	59.31	86.91	-18.8	[-28.88, -8.80]	< 0.001	0.005	0.17
OCD	General practitioner	52	41	100.60	153.79	74.63	88.62	-26.0	[-78.87, 26.95]	0.310	0.527	0.20
	Pediatrician	42	47	62.02	78.99	38.94	44.90	-23.1	[-49.43, 3.25]	0.100	0.274	0.36
	Child and adolescent psychiatrist	26	28	88.73	126.86	56.89	74.38	-31.8	[-86.82, 23.14]	0.272	0.499	0.31
	Child and adolescent psychotherapist	37	36	55.59	103.88	46.14	63.04	-9.5	[-49.01, 30.10]	0.639	0.777	0.11
PTSD	General practitioner	260	162	93.82	123.33	70.54	104.21	-23.3	[-46.11, -0.45]	0.038	0.143	0.20
	Pediatrician	259	154	51.18	89.02	40.17	55.29	-11.0	[-26.61, 4.58]	0.122	0.312	0.14
	Child and adolescent psychiatrist	87	58	84.11	121.91	55.83	81.93	-28.3	[-64.09, 7.52]	0.097	0.271	0.26
	Child and adolescent psychotherapist	125	77	71.52	102.68	54.68	83.57	-16.8	[-44.07, 10.38]	0.205	0.434	0.18
Male children and adolescents												
Anorexia nervosa	General practitioner	15	17	84.07	116.66	60.24	80.65	-23.8	[-92.64, 44.98]	0.513	0.703	0.24
	Pediatrician	19	23	22.84	39.36	44.48	56.31	21.6	[-8.39, 51.66]	0.152	0.366	0.44
	Child and adolescent psychiatrist	9	12	103.67	102.27	92.83	127.46	-10.8	[-112.40, 90.73]	0.831	0.896	0.09
	Child and adolescent psychotherapist	9	15	80.11	81.56	33.00	56.05	-47.1	[-102.04, 7.82]	0.152	0.366	0.71
Anxiety disorder	General practitioner	1125	666	110.01	136.10	105.26	123.60	-4.7	[-17.36, 7.86]	0.450	0.660	0.04
	Pediatrician	2045	1342	43.77	74.84	42.72	64.58	-1.0	[-5.93, 3.84]	0.665	0.786	0.01
	Child and adolescent psychiatrist	742	465	87.06	125.41	65.55	98.54	-21.5	[-34.93, -8.08]	0.001	0.015	0.19
	Child and adolescent psychotherapist	532	353	98.29	139.85	75.45	101.76	-22.8	[-39.80, -5.88]	0.005	0.041	0.18
Depressive disorder	General practitioner	723	388	84.59	120.12	73.28	102.50	-11.3	[-25.41, 2.78]	0.099	0.273	0.10
	Pediatrician	443	281	70.06	105.87	60.01	76.39	-10.1	[-24.33, 4.23]	0.139	0.347	0.11
	Child and adolescent psychiatrist	307	198	90.10	134.80	50.32	77.32	-39.8	[-60.46, -19.10]	< 0.001	**0.002**	**0.34**
	Child and adolescent psychotherapist	280	190	90.75	138.25	49.23	81.55	-41.5	[-63.38, -19.66]	< 0.001	**0.002**	**0.35**
OCD	General practitioner	45	30	100.20	133.08	108.10	131.88	7.9	[-53.36, 69.16]	0.801	0.876	0.06
	Pediatrician	48	36	68.06	98.89	53.03	65.12	-15.0	[-52.25, 22.18]	0.404	0.622	0.17
	Child and adolescent psychiatrist	33	20	39.36	48.75	30.30	40.96	-9.1	[-34.62, 16.49]	0.472	0.678	0.20
	Child and adolescent psychotherapist	29	18	98.62	139.09	101.28	102.59	2.7	[-71.77, 77.08]	0.940	0.962	0.02
PTSD	General practitioner	124	80	96.15	135.02	110.64	139.61	14.5	[-23.97, 52.95]	0.464	0.672	0.11
	Pediatrician	205	121	47.11	76.10	45.26	73.35	-1.9	[-18.73, 15.02]	0.828	0.896	0.02
	Child and adolescent psychiatrist	47	43	95.72	139.13	92.88	117.12	-2.8	[-56.24, 50.56]	0.917	0.943	0.02
	Child and adolescent psychotherapist	58	33	86.14	149.62	55.30	68.55	-30.8	[-84.94, 23.27]	0.183	0.410	0.24

Note. Cohen’s d: small effect: ≥0.2, medium effect: ≥0.5, large effect: ≥0.8; n, sample size; M, mean (in days); SD, standard deviation; ΔM, mean difference (intra-COVID-19 – pre-COVID-19); only comparisons with *n* > 5 are included and only comparisons with *p.adj* < 0.05 and |d| ≥ 0.2 are highlighted

### Duration until first outpatient contact after inpatient discharge stratified by region of residence and SES

Subgrouped by region of residence, reductions in TDO were observed in both rural and urban areas: In rural areas, however, significant changes in TDO were limited to child and adolescent psychotherapists for anorexia nervosa (CAPPs: -35.5 days, d = 0.48, *p*=.006) and depressive disorders (CAPPs: -34.3 days, d = 0.28, *p*<.001). In urban areas, significant reductions were also observed for other professional groups in anorexia nervosa (CAPs: -38.5 days, d = 0.36, *p*=.007; CAPPs: -32.8 days, d = 0.32, *p* = .10, PEDs: -23.3 days, d = 0.30, *p*=.009), depressive disorders (CAPs: -26.2 days, d = 0.23, *p*<.001), and additionally for anxiety disorders (CAPs: -28.7 days, d = 0.23, *p*<.001; CAPPs: -23.5 days, d = 0.21, *p*<.001).

Subgrouped by SES, significant changes in TDO from pre-COVID to intra-COVID were only observed in the low and medium SES groups: In the low SES group, this related to depressive disorders in CAPPs (-32.6 days, d = 0.3, *p* = .008), while in the medium SES group, there were significant reductions with regard to depressive disorders (CAPs: -29.3 days, d = 0.25, *p*<.001; CAPPs: -23.2 days, d = 0.21, *p*<.001), anxiety disorders (CAPPs: -35.9 days, d = 0.27, *p*<.001) and anorexia nervosa (PEDs: -27 days, d = 0.34, *p*=.007). No significant changes in TDO were observed in the group with high SES. Despite a moderate reduction in TDO among anorexia nervosa patients with contact to child and adolescent psychotherapists (CAPPs: -57.2 days, d = 0.50, *p* = .022), this effect did not reach significance after FDR correction.

Similar to the effects observed for age and sex, significant reductions related to region of residence and SES were limited to or more pronounced in psychiatry-specific professional groups (with the exception of anorexia nervosa in contacting pediatricians in medium SES group). For a detailed overview of the comparisons of region of residence and SES, see Additional file 1.

### Duration of inpatient treatment stratified by disorder, age and sex

**Fig. 2:**
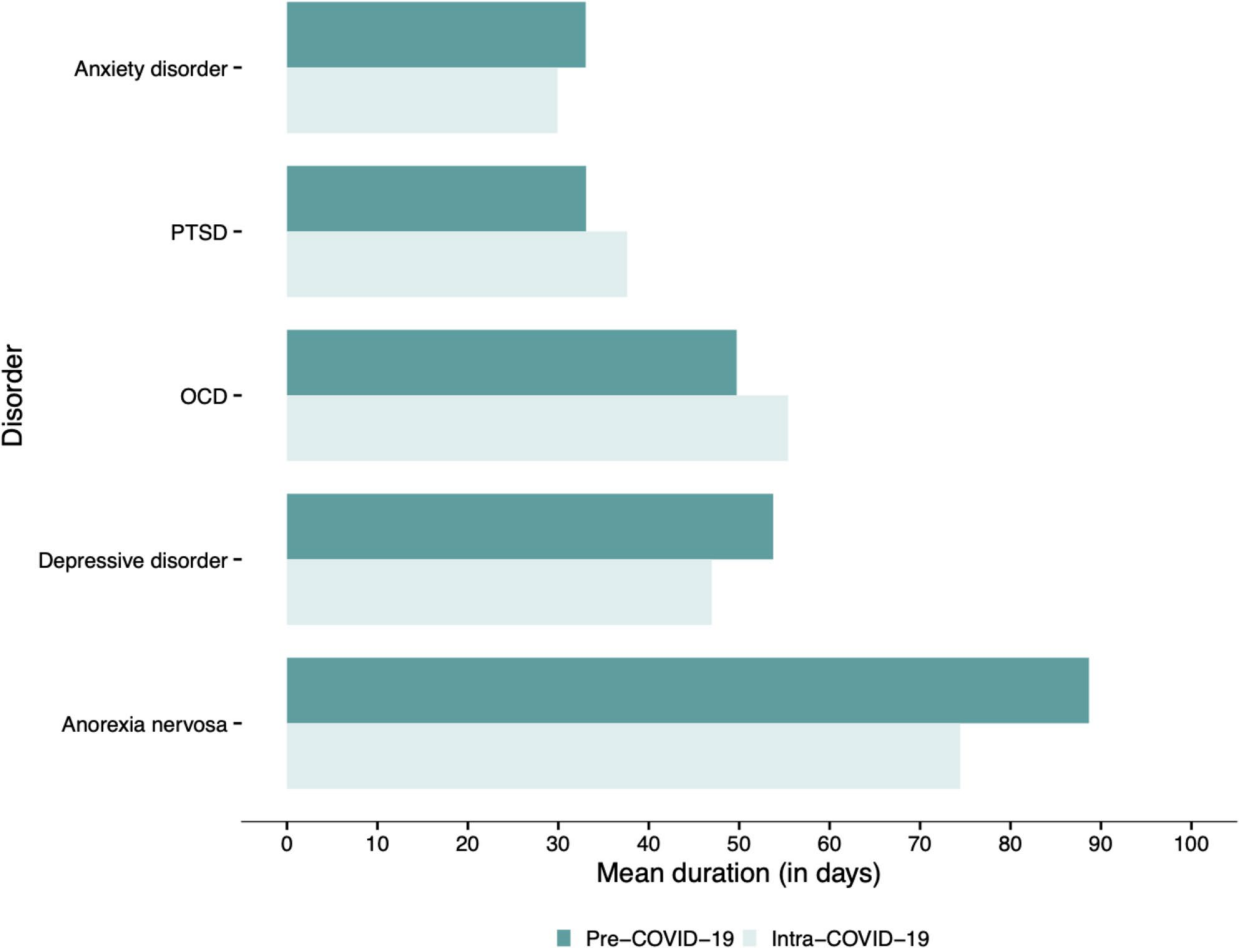
Duration of inpatient treatment (LOS) by disorder

A significant reduction in LOS (after FDR correction) was observed for depressive disorders (-6.8 days, d = 0.11, *p*<.001). LOS ranged from 33.0 days (PTSD) to 88.7 days (anorexia nervosa) pre-COVID and from 29.9 days (anxiety disorders) to 74.4 days (anorexia nervosa) intra-COVID. When stratified by age and sex, no significant differences in LOS were found. For an overview of LOS by disorder group, see Fig. 2; for LOS by disorder group, age, and sex, see Table 2.

### Duration of inpatient treatment stratified by region of residence and SES

Subgrouped by region of residence, a significant reduction in LOS was found in rural areas for anorexia nervosa (-26 days, d = 0.28, *p*=.002). For urban areas, no significant differences were found. For a detailed overview of LOS by region of residence and SES, see Additional file 2.

**Table 2:** Duration of inpatient treatment (LOS) by disorder, age and sex

ICD-10			Pre-COVID		Intra-COVID						
	*n* 2019	*n* 2021	M	SD	M	SD	ΔM	ΔM [95%]	*p*	*p*.adj	|d|
Anorexia nervosa	413	421	88.67	95.61	74.44	73.76	-14.2	[-25.81, -2.66]	0.016	0.118	0.17
Anxiety disorder	5309	3532	33.03	49.01	29.91	43.14	-3.1	[-5.11, -1.13]	0.002	0.050	0.07
Depressive disorder	3260	2438	53.76	64.29	46.96	55.17	-6.8	[-9.98, -3.62]	< 0.001	0.004	0.11
OCD	167	137	49.72	55.09	55.41	57.62	5.7	[-7.02, 18.40]	0.383	0.659	0.10
PTSD	710	453	33.07	56.53	37.61	63.85	4.5	[-2.47, 11.55]	0.217	0.482	0.08
0–9 yrs.											
Anorexia nervosa	7	9	46.86	79.93	26.11	41.91	-20.8	[-81.16, 39.67]	0.550	0.803	0.34
Anxiety disorder	2505	1437	24.93	41.82	22.50	35.13	-2.4	[-4.99, 0.14]	0.052	0.226	0.06
Depressive disorder	133	62	46.02	54.48	39.27	46.95	-6.7	[-22.48, 9.00]	0.377	0.656	0.13
OCD	30	31	49.57	66.81	31.45	49.68	-18.1	[-47.59, 11.36]	0.236	0.496	0.31
PTSD	276	141	25.67	56.50	35.81	64.39	10.1	[-1.89, 22.17]	0.114	0.332	0.17
10–13 yrs.											
Anorexia nervosa	120	141	95.62	106.59	70.38	73.01	-25.2	[-47.14, -3.32]	0.029	0.157	0.28
Anxiety disorder	1607	1109	41.14	53.54	35.28	45.09	-5.9	[-9.71, -2.02]	0.002	0.050	0.12
Depressive disorder	887	687	54.91	65.52	45.43	54.08	-9.4	[-15.54, -3.42]	0.002	0.050	0.16
OCD	70	31	43.66	51.57	46.35	39.48	2.7	[-17.70, 23.09]	0.775	0.898	0.06
PTSD	198	118	38.93	55.93	34.86	53.95	-4.1	[-16.66, 8.51]	0.523	0.795	0.07
14–17 yrs.											
Anorexia nervosa	286	271	86.78	90.95	78.15	74.47	-8.6	[-22.48, 5.22]	0.220	0.485	0.10
Anxiety disorder	1197	986	39.10	53.73	34.68	49.49	-4.4	[-8.79, -0.05]	0.046	0.212	0.09
Depressive disorder	2240	1689	53.76	64.33	47.87	55.87	-5.9	[-9.74, -2.05]	0.002	0.050	0.10
OCD	67	75	56.12	53.02	69.05	63.29	12.9	[-6.40, 32.26]	0.187	0.432	0.22
PTSD	236	194	36.81	56.36	40.60	68.99	3.8	[-8.06, 15.64]	0.539	0.797	0.06
Female children and adolescents											
Anorexia nervosa	387	389	90.98	96.78	76.34	74.62	-14.6	[-26.80, -2.49]	0.019	0.129	0.17
Anxiety disorder	2617	1820	33.21	51.06	30.53	45.40	-2.7	[-5.60, 0.24]	0.066	0.262	0.05
Depressive disorder	2229	1840	57.16	69.24	49.29	57.56	-7.9	[-11.25, -4.48]	< 0.001	0.009	0.12
OCD	84	77	49.57	57.30	54.12	50.62	4.6	[-12.22, 21.31]	0.594	0.845	0.08
PTSD	424	276	34.06	58.84	38.91	63.04	4.9	[-4.33, 14.03]	0.308	0.578	0.08
Male children and adolescents											
Anorexia nervosa	26	32	54.27	68.76	51.31	58.43	-3.0	[-35.69, 29.78]	0.863	0.932	0.05
Anxiety disorder	2692	1712	32.86	46.94	29.26	40.60	-3.6	[-6.30, -0.90]	0.007	0.072	0.08
Depressive disorder	1031	598	46.41	51.28	39.79	46.35	-6.6	[-11.61, -1.63]	0.008	0.072	0.13
OCD	83	60	49.87	53.10	57.07	65.93	7.2	[-12.33, 26.73]	0.487	0.775	0.12
PTSD	286	177	31.61	52.99	35.59	65.22	4.0	[-6.88, 14.85]	0.494	0.775	0.07

Note. Cohen’s d: small effect: ≥0.2, medium effect: ≥0.5, large effect: ≥0.8; n, sample size; M, mean (in days); SD, standard deviation; ΔM, mean difference (intra-COVID-19 – pre-COVID-19); only comparisons with *n* > 5 are included and only comparisons with *p.adj* < 0.05 and |d| ≥ 0.2 are highlighted

### Inpatient readmission and emergency room admission after hospitalization and before first outpatient contact stratified by disorder and readmission facility

For the results of an additional analysis on readmissions after inpatient discharge and before first outpatient contact, see Additional file 3.

## Discussion

This study investigated the average time between discharge and first outpatient contact (TDO), in relation to the length of inpatient stay (LOS) among children and adolescents in Germany.

This exploratory analysis considered the impact of COVID-19-related changes and stratified results by sex, age, region of residence, SES, specialty consulted, and index diagnosis. Studies on such treatment parameters, which may reflect a significant factor in the coordination between in- and outpatient (follow-up) treatment, are scarce. It is important to note that these findings should be interpreted with caution, given the exploratory nature of this analysis and its methodological constraints (descriptive measures, broad timeframes, multiple comparisons).

We observed statistically significant reductions in TDO and marginal reductions in LOS from pre-COVID to intra-COVID, with effects varying by disorder and outpatient specialty. These findings correspond to a period of reduced inpatient capacity resources during COVID-19 pandemic (i.e., lower occupancy rates due to the risk of infections or staff absences due to illness) [67–69]. Changes in inpatient capacity may have been related to patients seeking help with greater severity or acuity [1, 70, 71], despite the risk of contracting COVID in the hospital [72], and reduced capacities. Changes in absolute inpatient admissions thresholds [73] or the use of telehealth billing [74] during the intra-COVID period may also have affected the LOS and TDO. The modest reduction in LOS may be associated with a faster discharge of patients, potentially contributing to a shorter TDO.

By disorder, we found reductions in both duration parameters for depressive disorders, anxiety disorders and anorexia nervosa. Regarding LOS, this result is consistent with another German study [69], which reported an overall reduction in LOS (-1.7 days) in child and adolescent psychiatry departments. The observed reductions might be related to the increased incidence of these disorders during the pandemic [36]. Reductions in LOS and TDO suggest potential adaptations in inpatient capacity utilization and discharge management. A faster transition to outpatient care aligns with findings from [46], which showed an increased demand for treatment of children and adolescents during the pandemic, reflected by doubled waiting times, more frequent therapy extensions, and worsening of symptoms. The increased caseload of child and adolescent psychotherapists [46] could have contributed to a more rapid transition from inpatent to outpatient care.

In general, and regardless of the COVID-19 pandemic, there were substantial intervals between discharge and first outpatient contact for all studied disorders. This finding suggests challenges in ensuring timely outpatient follow-up. Possible barriers to follow-up include lack of adherence to discharge planning [75], limited access to care (particularly in rural areas) [76], long waiting lists [48] or logistical resources (e.g. lack of time or to drive the child to treatment) [77]. Substantial waiting times for specialist treatment were also found for adult insured persons, varying considerably between different practitioners. In a survey study in Germany (insurees aged 40–66), 45.4% reported waiting over 3 weeks for psychiatric specialist care. Furthermore, 58% of those who waited over 3 weeks (for any specialist consultation) considered the waiting time to be too long [78].

The most pronounced disease-specific changes in TDO were observed in anorexia nervosa among specialized practitioners (pediatricians, child and adolescent psychiatrists and child and adolescent psychotherapists). Khalsa et al. [79] reported in a systematic review a relapse rate of anorexia nervosa ranging from 9% to 52% after inpatient treatment, and an increase in relapse rates with increasing TDO. Walsh et al. [80] identified an inflection point for relapse in anorexia nervosa, occurring about 60 days after hospital discharge, with the likelihood of relapse decreasing after that point. Meule et al. [81] showed that the beneficial effects of an inpatient treatment of anorexia nervosa remained stable until a 1-year follow, which the authors also associated with the high rate of their sample receiving outpatient aftercare (94%). The relatively high TDO for anorexia nervosa could be related to a lack of tailored, patient-centered approaches or frequent treatment fatigue after inpatient treatment [16]. Future research should explore innovative approaches to enhance the effectiveness and long-term stability of anorexia nervosa treatment, such as home treatment programs [82], personalized relapse prevention programs and waiting list interventions, and standardization of relapse, remission, and recovery [79, 83–85].

Although not significant after FDR correction, the largest reductions in LOS for anorexia were observed in the 10–13 age group. This result aligns with findings [86], which reported higher hospitalization rates for younger children and adolescents (0–14 years) during the COVID-19 pandemic. An existing longer duration of illness in 14- to 17-year-olds might have precluded a more uniform reduction in LOS between the age groups [87]. Given the greater reductions in TDO for 14-17-year old girls with anorexia nervosa in contact with pediatricians and CAPPs, delayed post-pandemic effects should be monitored. This is also important since age is considered crucial for the long-term manifestation of anorexia nervosa [88].

Similar trends were observed for depressive disorders and anxiety disorders, with decreases in TDO and LOS (across age and sex groups), potentially indicating a shift from inpatient to outpatient care. Findings for OCD and PTSD were less clear. For these two disorders, no significant changes were observed by disorder, sex or age. However, changes in LOS varied greatly and were based on a small number of cases, making it hard to identify any trends. Furthermore, no clear differences could be found between female and male patient groups (except for anorexia nervosa). Female cases showed larger TDO reductions for anxiety disorders, while male cases showed larger reductions for depressive disorders. Male patients with depressive disorders, however, already had a shorter LOS by about ~ 10–11 days in the pre-COVID period. A shift towards outpatient follow-up treatment could have contributed to this finding, given the slightly greater reduction in the TDO observed in male cases.

Another possible interpretation is that the LOS shortened primarily for patient groups who had already longer LOS before COVID-19 pandemic. For example, the changes in LOS for anxiety disorders were comparatively small in terms of absolute days (e.g. -3.1 days for anxiety disorders vs. -14.2 days for anorexia nervosa). This may be due to a floor effect, as anxiety disorders had substantially shorter LOS already before COVID-19 pandemic. A methodologically similar study [73] also described floor effects in terms of LOS for certain federal states in Germany, i.e., diminishing opportunities for further LOS reductions, accompanied by increasing admission pressures in child and adolescent psychiatry hospital systems. These trends in LOS and TDO reduction suggest that efforts to optimize LOS to disorder-specific minimums may have disproportionately affected access to timely care for more treatment-intensive cases, potentially accelerating transitions to outpatient care, as discussed above. This pattern could also apply to the 10–13 and 14–17 age groups with anorexia nervosa, where pre-COVID, 10-13-year-old girls had longer LOS (about ~ 10 days) than 14-17-year-old girls. This finding suggests that the significant reductions in TDO for anorexia nervosa among 14- to 17-year-olds during follow-up visits to PEDs and CAPs may be associated with a quicker shift from inpatient to outpatient care.

There were no significant changes between pre-COVID and intra-COVID observed in the subgroups of SES, limiting the identification of any trends. A floor effect may have contributed to these findings, as cases in the low SES group exhibited descriptively the shortest LOS for anorexia nervosa, anxiety disorders, and depressive disorders compared to the other two SES groups (see Additional file 2). These results should be considered alongside studies that have identified low SES as a COVID-related risk factor [41] or reported an increased risk of new diagnoses of depressive disorders, anxiety disorders and anorexia nervosa during COVID-19 pandemic for female children and adolescents with high SES [36, 45]. One possible explanation for this discrepancy is that the German Socioeconomic Deprivation Index used in the InGef database, based on regional postal code, may only insufficiently reflect SES in Germany.

Another noteworthy observation of our study was the consideration of psychological/psychiatric (CAPPs & CAPs) vs. general medical professional groups (GPs & PEDs). In line with other findings [46], the significant reductions in TDO were mainly related to psychological/psychiatric professional groups (except for follow-up contacts with pediatricians for anorexia nervosa). This result seems plausible, potentially representing a counterbalance, as general practitioners were particularly strained by COVID-19 cases. At the same time, the special role of general practitioners should be emphasized here, where, in the case of female children and adolescents (10–21 years) with anorexia nervosa, contact is proportionally most common [89]. Additionally, unacceptably long waiting times for referrals from general practitioners to specialist practitioners are a common problem [90]. Alternatively, this could reflect a necessary treatment specification, where limited capacity in inpatient departments and in general practice is addressed by specialized outpatient follow-up care. Important in this regard is a recent review [22], which points out the insufficient conditions in outpatient treatment of children and adolescents in Germany. Given the discrepancy between treatment needs and utilization among children and adolescents, they also mention perceived barriers hindering access to treatment. Such barriers may also explain our findings regarding general practitioners, where visits might have been avoided or postponed due to the risk of COVID infection. In conclusion, increasing the ratio of specialized outpatient therapists (child and adolescent psychiatrists/psychotherapists) to the population of children and adolescents in Germany is important. However, expanding capacity alone may not be sufficient [22], as underprovision of mental health services for children and adolescents is common in many European countries, even those with a well-developed outpatient sectors [20]. Masseneck et al. [76] also showed that accessibility in terms of driving time to various psychiatric care providers in Germany is largely ensured, although there are regional disparities. Similarly to Rodney-Wolf and Schmitz [22], Mori et al. [91] highlight the international relevance of this issue and the importance of interventions to promote help-seeking behavior among children and adolescents, such as mental health literacy and school- and community-based programs. Furthermore, digital interventions could help in bridging existing gaps in continuity in care [91]. Chen et al. [92] identified discharge intervention elements such as risk assessment, individualized care, discharge preparation, community linkage, and follow-up support as aspects associated with increased patient and caregiver satisfaction, better patient health outcomes, and increased cost-effectiveness. In case of anorexia nervosa, timely outpatient follow-up treatment should play an important role in discharge planning and relapse prevention [80], while especially tailored and patient-centered approaches should be considered [16]. In this context it should be kept in mind that interventions such as telemedicine are not necessarily preferred by children and adolescents in every area of care where adults would prefer them [93]. Our study also suggests that enhanced collaboration between schools, primary care (general practitioners, pediatricians), inpatient departments (child and adolescent psychiatry and pediatrics) and outpatient professional groups (child and adolescent psychotherapists and -psychiatrists) is needed to systematically improve the infrastructure of mental health services. Future studies are needed to assess the long-term consequences of the changes caused by COVID-19 pandemic on the psychological and psychiatric care of children and adolescents in need across different levels of care and settings. These studies should monitor changes in mental and physical health care capacity and utilization rates post-pandemic to inform resource allocation.

## Strengths and limitations

To the best of our knowledge, this is the first study to examine TDO and LOS based on representative claims data from statutory health insurers in Germany. By investigating these two parameters, the study provides insights concerning the transition from inpatient to outpatient follow-up treatment for children and adolescents in general, but also in relation to the COVID-19 pandemic. Studies that combine such treatment parameters are scarce, both nationally and internationally. Due to the structure of the data, the results are free of non-response or recall bias, as the data were collected prospectively and without the patients’ memory or voluntary participation in the study. Often, studies on such treatment parameters can only selectively report insights for certain professional groups, departments or individual disorders. Another strength of the present study is the inclusion of various parameters and consideration of multiple subgroups, which resulted in a more granular and cohesive context.

In addition to the strengths mentioned, the study has several limitations. A significant limitation is the potential for double counting between the disorder categories of the first (‘other disorders’) and second (‘eating disorders’) data extractions, which may have affected the accuracy or specificity of the analyses. Due to the limitation of aggregated secondary data, quantifying this potential overlap was not feasible. Another key limitation of this study is the lack of consideration for standard follow-up timeframes (e.g. 7, 30, 60, or 90 days post-discharge) for outpatient contacts and readmissions. Consequently, the calculation of means for TDO was based on potentially overly broad time windows for both pre- and intra-COVID periods. Given the expected right-skewed distribution of re-engagement with care (e.g., readmissions [80]), the reported mean TDO values may be susceptible to overestimation bias. A further limitation of this study is the potential for selection bias due to changes in follow-up rates during the COVID-19 pandemic. Our procedure does not capture cases where individuals did not seek medical or therapeutic follow-up care, which may have been more common during the intra-COVID period and could bias the results toward shorter TDO values among those who did receive care. Moreover, this is more likely for discharges that occurred at the end of the study period. Additionally, the statistical methods employed in this study (means, standard deviations, Welch’s t-tests, and Cohen’s d) are based on aggregated secondary data and may be susceptible to bias due to this potential right-skewed distribution of TDO and LOS. Time-to-event methods would be preferable in future studies with access to individual-level data. Due to the lack of individual-level data, we were also unable to model the direct relationship between LOS and TDO. Furthermore, the validity of using all outpatient contacts as a measure of continuity of care is limited, as psychiatric follow-up care is typically provided by specialized mental health professionals, and GPs often serve as a sentinel for identifying severe conditions such as acute underweight or suicidal ideation. This validity is further limited by the fact that the included outpatient follow-up contacts cannot be attributed to the respective diagnosis. A further limitation concerns the lack of differentiation between in-person and telemedicine billing in our study data. An increase in telemedicine follow-up services during COVID-19 pandemic could therefore limit the comparability of the periods and the generalizability of the results. Another important limitation is that, due to the data extraction method, we were only able to make statements about the aforementioned disorders and did not consider other disorders that are common in childhood and adolescence, such as attention-deficit/hyperactivity disorders (ADHD), conduct disorders or substance use-related disorders. This fact may have resulted in an underrepresentation of male cases in our data, who are more likely to receive diagnoses of these disorders [94–96]. Another limitation of this study was its exclusive focus on binary sex categories (female and male), without inclusion of data on individuals with other genders. Due to the data collection based on diagnostic codes (claims data), inpatient or outpatient treatment cannot be attributed solely to the disorders included in the data. Moreover, the validity of the diagnoses cannot be verified for secondary data. The prevalence of the included disorders, from which the computed durations are obtained, may thus have been underestimated due to strict case definitions. A further limitation concerns the assessment of SES, which is based on an area-level deprivation index. This approach inherently carries the risk of ecological misclassification [97], biasing the accuracy of the comparisons between pre- and intra-COVID periods. Moreover, this study is limited to data from statutory health insurers. As a result, the child and adolescent population with private health insurance are not represented in our findings. This implies that the results cannot be generalized to all patients aged 0.0 to 17.9 years, as there are differences regarding SES and morbidity in favor of privately insured patients [98]. Furthermore, despite employing corrections for multiple testing, the inherent limitations of this approach mean we cannot entirely eliminate the possibility of both false-positive and false-negative findings. This is particularly relevant when interpreting trends observed in the data. In addition, the exploratory analysis approach without a priori hypotheses limits the generalizability of the study. Finally, the accuracy of our results is limited for the subgroups where the number of cases was small. This particularly affected the disorders of OCD and PTSD, but also, for example, the age group of 0–9-year-olds.

## Conclusions

Our analysis of TDO in children and adolescents in Germany before and during the COVID-19 pandemic revealed substantial time gaps (> 2 months) between discharge from child and adolescent psychiatric inpatient care to first outpatient contact with selected professional groups. These transition times to outpatient care warrant attention, particularly given the importance of continuity of care in severe mental disorders like anorexia nervosa. While these intervals decreased during the COVID-19 pandemic, these effects should be evaluated considering their methodological constraints. Future studies should examine whether the trends identified in this study are reflected in a sustained trend towards a swifter inter-sectoral transition for youths with mental health problems. Data are needed that explore trends post-COVID and effects of TDO and LOS on outcomes such as rehospitalization and other indicators of quality of care and beneficial treatment results.

## Supplementary Information

Below is the link to the electronic supplementary material.

**Supplementary Material 1:** Duration until first outpatient contact after inpatient discharge (TDO) of children and adolescents with psychiatric disorders before vs. during the COVID-19 pandemic, by sex, age, residency, status, disorder and professional group.

**Supplementary Material 2:** Duration of inpatient stay (LOS) of children and adolescents with psychiatric disorders before vs. during the COVID-19 pandemic, by sex, age, residency, status and disorder.

**Supplementary Material 3:** After hospitalization, inpatient readmission and emergency room admission before first outpatient contact of female and male patients by disorder and readmission facility.

## Data Availability

The datasets utilized and/or analyzed in this study can be requested from the InGef under reasonable conditions.

## Footnotes

1 Signorini et al. [19] defined “young people” as younger than 18 years or the legal transition boundary age, but for Germany age limit was extended to 21.

2 The term „child and adolescent psychologists“ follows the wording of Signorini et al. [19], since the study does not state whether this also includes child and adolescent psychotherapists.

## Abbreviations

GISD: German Index of Socioeconomic Deprivation
ICD: International Classification of Diseases
M: Mean
SD: Standard Deviation
n: Sample size
SES: Socioeconomic Status
GPs: General practitioners
PEDs: Pediatricians
CAPs: Child and Adolescent Psychiatrists
CAPPs: Child and Adolescent Psychotherapists
TDO: Time from discharge to first outpatient contact (Mean duration until first outpatient contact after inpatient discharge in days)
LOS: Length of stay (Mean inpatient duration in days)
EBM: Standardized evaluation scale [Einheitlicher Bewertungsmaßstab]
OCD: Obsessive-compulsive disorder
PTSD: Post-traumatic stress disorder
