# Supplementary material for: Continuity of care: time to first outpatient appointment after child and adolescent psychiatric hospital stays in Germany
Source: BMC Health Serv Res. 2026 Mar 13;26:486. doi: 10.1186/s12913-026-14322-7 (PMC13063905; doi:10.1186/s12913-026-14322-7)
Supplement: Supplementary file 1 — Supplementary Material 1: Duration until first outpatient contact after inpatient discharge (TDO) of children and adolescents with psychiatric disorders before vs. during the COVID-19 pandemic, by sex, age, residency, status, disorder and professional group. [file 12913_2026_14322_MOESM1_ESM.docx]

**Supplemental table 1**. Duration until first outpatient contact after inpatient discharge (TDO) of children and adolescents with psychiatric disorders before vs. during the COVID-19 pandemic, by sex, age, residency, status, disorder and professional group

| Sex | Age | Residency | Status | ICD | Professional group | *Pre-COVID* | *Intra-COVID* | *Pre-COVID* | | *Intra-COVID* | | *p* | *p.adj* | *\|d\|* | *ΔM* | 95% CI *ΔM* |
| --- | --- | --- | --- | --- | --- | --- | --- | --- | --- | --- | --- | --- | --- | --- | --- | --- |
|  |  |  |  |  |  | *n* | *n* | *M* | *SD* | *M* | *SD* |  |  |  |  |  |
| Total | Total | Total | Total | Anorexia nervosa | General practitioner | 291 | 259 | 90.02 | 131.20 | 75.53 | 97.50 | .139 | .347 | 0.12 | -14.50 | [-34.01, 5.02] |
| Total | Total | Total | Total | Anorexia nervosa | Pediatrician | 212 | 245 | 66.33 | 100.48 | 43.49 | 61.53 | .004 | **.037** | **0.28** | -22.83 | [-37.89, -7.77] |
| Total | Total | Total | Total | Anorexia nervosa | Psychiatrist/Neurologist | 32 | 21 | 165.94 | 139.08 | 154.43 | 125.47 | .756 | .843 | 0.09 | -11.51 | [-85.22, 62.20] |
| Total | Total | Total | Total | Anorexia nervosa | Child and adolescent psychiatrist | 157 | 166 | 91.25 | 123.23 | 62.07 | 88.78 | .016 | .085 | 0.27 | -29.18 | [-52.51, -5.85] |
| Total | Total | Total | Total | Anorexia nervosa | Psychological psychotherapist | 28 | 25 | 132.29 | 150.18 | 131.32 | 124.20 | .980 | .986 | 0.01 | -0.97 | [-75.69, 73.76] |
| Total | Total | Total | Total | Anorexia nervosa | Child and adolescent psychotherapist | 202 | 241 | 83.16 | 116.63 | 49.76 | 71.74 | < .001 | **.009** | **0.35** | -33.40 | [-51.14, -15.66] |
| Total | Total | Total | Total | Anxiety disorder | General practitioner | 2,467 | 1,514 | 102.34 | 132.72 | 94.25 | 114.28 | .042 | .150 | 0.06 | -8.09 | [-16.15, -0.02] |
| Total | Total | Total | Total | Anxiety disorder | Pediatrician | 3,858 | 2,595 | 47.66 | 80.73 | 42.92 | 67.04 | .010 | .064 | 0.06 | -4.74 | [-8.50, -0.98] |
| Total | Total | Total | Total | Anxiety disorder | Psychiatrist/Neurologist | 202 | 84 | 192.91 | 184.10 | 141.25 | 140.25 | .011 | .064 | 0.30 | -51.66 | [-95.54, -7.78] |
| Total | Total | Total | Total | Anxiety disorder | Child and adolescent psychiatrist | 1,395 | 975 | 85.13 | 124.06 | 63.57 | 92.99 | < .001 | < .001 | 0.19 | -21.56 | [-30.75, -12.37] |
| Total | Total | Total | Total | Anxiety disorder | Psychological psychotherapist | 137 | 63 | 119.73 | 155.07 | 98.90 | 129.05 | .322 | .538 | 0.14 | -20.83 | [-64.81, 23.16] |
| Total | Total | Total | Total | Anxiety disorder | Child and adolescent psychotherapist | 1,246 | 911 | 94.18 | 140.43 | 67.10 | 98.67 | < .001 | **< .001** | **0.22** | -27.08 | [-37.72, -16.44] |
| Total | Total | Total | Total | Depressive disorder | General practitioner | 2,434 | 1,628 | 83.00 | 121.48 | 73.70 | 100.06 | .008 | .052 | 0.08 | -9.30 | [-16.42, -2.19] |
| Total | Total | Total | Total | Depressive disorder | Pediatrician | 1,407 | 1,182 | 74.80 | 107.62 | 60.10 | 80.70 | < .001 | .003 | 0.15 | -14.70 | [-22.15, -7.26] |
| Total | Total | Total | Total | Depressive disorder | Psychiatrist/Neurologist | 358 | 210 | 194.00 | 187.16 | 146.00 | 137.28 | < .001 | **.009** | **0.28** | -48.00 | [-77.04, -18.96] |
| Total | Total | Total | Total | Depressive disorder | Child and adolescent psychiatrist | 1,129 | 956 | 86.46 | 128.99 | 61.47 | 91.89 | < .001 | **< .001** | **0.22** | -24.99 | [-34.77, -15.22] |
| Total | Total | Total | Total | Depressive disorder | Psychological psychotherapist | 202 | 127 | 153.52 | 170.38 | 92.09 | 123.83 | < .001 | **.005** | **0.40** | -61.44 | [-95.65, -27.23] |
| Total | Total | Total | Total | Depressive disorder | Child and adolescent psychotherapist | 1,203 | 1,042 | 81.08 | 127.56 | 57.47 | 86.01 | < .001 | **< .001** | **0.21** | -23.61 | [-32.76, -14.47] |
| Total | Total | Total | Total | OCD | General practitioner | 97 | 71 | 100.41 | 143.81 | 88.77 | 109.41 | .552 | .728 | 0.09 | -11.64 | [-51.56, 28.29] |
| Total | Total | Total | Total | OCD | Pediatrician | 90 | 83 | 65.24 | 89.71 | 45.05 | 54.68 | .073 | .222 | 0.27 | -20.20 | [-42.56, 2.17] |
| Total | Total | Total | Total | OCD | Psychiatrist/Neurologist | 12 | 13 | 175.58 | 175.67 | 156.15 | 107.93 | .745 | .834 | 0.13 | -19.43 | [-132.69, 93.83] |
| Total | Total | Total | Total | OCD | Child and adolescent psychiatrist | 59 | 48 | 61.12 | 94.12 | 45.81 | 63.50 | .320 | .536 | 0.19 | -15.31 | [-46.49, 15.87] |
| Total | Total | Total | Total | OCD | Child and adolescent psychotherapist | 66 | 54 | 74.50 | 121.55 | 64.52 | 81.78 | .593 | .751 | 0.09 | -9.98 | [-47.95, 27.98] |
| Total | Total | Total | Total | PTSD | General practitioner | 384 | 242 | 94.57 | 127.05 | 83.79 | 118.33 | .281 | .510 | 0.09 | -10.78 | [-30.69, 9.13] |
| Total | Total | Total | Total | PTSD | Pediatrician | 464 | 275 | 49.38 | 83.50 | 42.41 | 63.80 | .202 | .431 | 0.09 | -6.97 | [-18.42, 4.48] |
| Total | Total | Total | Total | PTSD | Psychiatrist/Neurologist | 31 | 21 | 265.39 | 229.33 | 108.43 | 115.80 | .002 | **.024** | **0.82** | -156.96 | [-263.39, -50.52] |
| Total | Total | Total | Total | PTSD | Child and adolescent psychiatrist | 134 | 101 | 88.19 | 127.81 | 71.60 | 99.63 | .265 | .496 | 0.14 | -16.58 | [-46.69, 13.52] |
| Total | Total | Total | Total | PTSD | Psychological psychotherapist | 24 | 13 | 161.00 | 170.34 | 143.31 | 147.72 | .745 | .834 | 0.11 | -17.69 | [-127.67, 92.28] |
| Total | Total | Total | Total | PTSD | Child and adolescent psychotherapist | 183 | 110 | 76.15 | 119.34 | 54.86 | 79.05 | .068 | .211 | 0.20 | -21.29 | [-46.37, 3.79] |
| Female | Total | Total | Total | Anorexia nervosa | General practitioner | 276 | 186 | 90.35 | 132.13 | 76.60 | 98.63 | .202 | .431 | 0.11 | -13.75 | [-36.02, 8.52] |
| Female | Total | Total | Total | Anorexia nervosa | Pediatrician | 193 | 121 | 70.61 | 103.66 | 43.39 | 62.16 | .004 | **.036** | **0.30** | -27.21 | [-47.67, -6.76] |
| Female | Total | Total | Total | Anorexia nervosa | Psychiatrist/Neurologist | 32 | 15 | 165.94 | 139.08 | 156.94 | 129.86 | .830 | .896 | 0.07 | -8.99 | [-92.57, 74.59] |
| Female | Total | Total | Total | Anorexia nervosa | Child and adolescent psychiatrist | 148 | 106 | 90.49 | 124.64 | 59.68 | 85.16 | .020 | .097 | 0.28 | -30.82 | [-58.23, -3.40] |
| Female | Total | Total | Total | Anorexia nervosa | Psychological psychotherapist | 28 | 18 | 132.29 | 150.18 | 127.57 | 128.11 | .910 | .941 | 0.03 | -4.72 | [-88.84, 79.40] |
| Female | Total | Total | Total | Anorexia nervosa | Child and adolescent psychotherapist | 193 | 150 | 83.30 | 118.17 | 50.87 | 72.63 | .002 | **.023** | **0.32** | -32.43 | [-53.94, -10.92] |
| Male | Total | Total | Total | Anorexia nervosa | General practitioner | 15 | 17 | 84.07 | 116.66 | 60.24 | 80.65 | .513 | .703 | 0.24 | -23.83 | [-92.64, 44.98] |
| Male | Total | Total | Total | Anorexia nervosa | Pediatrician | 19 | 23 | 22.84 | 39.36 | 44.48 | 56.31 | .152 | .366 | 0.44 | 21.64 | [-8.39, 51.66] |
| Male | Total | Total | Total | Anorexia nervosa | Child and adolescent psychiatrist | 9 | 12 | 103.67 | 102.27 | 92.83 | 127.46 | .831 | .896 | 0.09 | -10.83 | [-112.40, 90.73] |
| Male | Total | Total | Total | Anorexia nervosa | Child and adolescent psychotherapist | 9 | 15 | 80.11 | 81.56 | 33.00 | 56.05 | .152 | .366 | 0.71 | -47.11 | [-102.04, 7.82] |
| Total | 0-9 | Total | Total | Anorexia nervosa | Pediatrician | 5 | 8 | 45.60 | 91.91 | 9.88 | 7.57 | .435 | .653 | 0.64 | -35.73 | [-98.02, 26.57] |
| Total | 10-13 | Total | Total | Anorexia nervosa | General practitioner | 60 | 62 | 76.20 | 102.57 | 68.47 | 89.63 | .659 | .782 | 0.08 | -7.73 | [-41.88, 26.42] |
| Total | 10-13 | Total | Total | Anorexia nervosa | Pediatrician | 86 | 105 | 55.77 | 89.66 | 45.88 | 64.84 | .393 | .610 | 0.13 | -9.89 | [-31.84, 12.06] |
| Total | 10-13 | Total | Total | Anorexia nervosa | Child and adolescent psychiatrist | 42 | 50 | 91.79 | 122.70 | 52.78 | 77.66 | .079 | .234 | 0.39 | -39.01 | [-80.32, 2.31] |
| Total | 10-13 | Total | Total | Anorexia nervosa | Child and adolescent psychotherapist | 58 | 79 | 84.45 | 100.21 | 59.42 | 75.55 | .113 | .300 | 0.29 | -25.03 | [-54.45, 4.39] |
| Total | 14-17 | Total | Total | Anorexia nervosa | General practitioner | 227 | 196 | 93.34 | 137.91 | 78.13 | 100.07 | .191 | .413 | 0.12 | -15.22 | [-38.50, 8.07] |
| Total | 14-17 | Total | Total | Anorexia nervosa | Pediatrician | 121 | 132 | 74.69 | 107.75 | 43.64 | 60.31 | .006 | **.044** | **0.36** | -31.05 | [-52.34, -9.76] |
| Total | 14-17 | Total | Total | Anorexia nervosa | Psychiatrist/Neurologist | 32 | 18 | 165.94 | 139.08 | 174.50 | 123.43 | .823 | .893 | 0.06 | 8.56 | [-68.67, 85.80] |
| Total | 14-17 | Total | Total | Anorexia nervosa | Child and adolescent psychiatrist | 114 | 115 | 91.44 | 124.43 | 66.57 | 93.46 | .089 | .257 | 0.23 | -24.87 | [-53.36, 3.61] |
| Total | 14-17 | Total | Total | Anorexia nervosa | Psychological psychotherapist | 24 | 20 | 141.71 | 156.47 | 154.10 | 128.10 | .774 | .857 | 0.09 | 12.39 | [-73.26, 98.04] |
| Total | 14-17 | Total | Total | Anorexia nervosa | Child and adolescent psychotherapist | 143 | 158 | 83.16 | 123.23 | 44.37 | 69.93 | .001 | **.016** | **0.39** | -38.79 | [-61.16, -16.42] |
| Total | Total | Urban | Total | Anorexia nervosa | General practitioner | 200 | 169 | 86.16 | 134.32 | 72.26 | 89.27 | .236 | .470 | 0.12 | -13.90 | [-37.63, 9.83] |
| Total | Total | Urban | Total | Anorexia nervosa | Pediatrician | 150 | 173 | 65.04 | 98.16 | 41.10 | 56.66 | .009 | .058 | 0.30 | -23.94 | [-41.15, -6.73] |
| Total | Total | Urban | Total | Anorexia nervosa | Psychiatrist/Neurologist | 27 | 15 | 163.44 | 127.61 | 115.47 | 125.37 | .247 | .477 | 0.38 | -47.98 | [-128.03, 32.08] |
| Total | Total | Urban | Total | Anorexia nervosa | Child and adolescent psychiatrist | 117 | 122 | 95.92 | 125.96 | 57.44 | 85.76 | .007 | **.047** | **0.36** | -38.48 | [-65.70, -11.26] |
| Total | Total | Urban | Total | Anorexia nervosa | Psychological psychotherapist | 21 | 13 | 115.57 | 150.40 | 146.77 | 132.94 | .533 | .713 | 0.22 | 31.20 | [-68.48, 130.87] |
| Total | Total | Urban | Total | Anorexia nervosa | Child and adolescent psychotherapist | 135 | 169 | 85.17 | 129.11 | 52.38 | 75.98 | .010 | .060 | 0.32 | -32.79 | [-56.09, -9.49] |
| Total | Total | Rural | Total | Anorexia nervosa | General practitioner | 91 | 90 | 98.52 | 124.38 | 81.66 | 111.60 | .338 | .551 | 0.14 | -16.86 | [-51.30, 17.58] |
| Total | Total | Rural | Total | Anorexia nervosa | Pediatrician | 62 | 72 | 69.44 | 106.64 | 49.25 | 72.02 | .209 | .439 | 0.23 | -20.19 | [-50.64, 10.27] |
| Total | Total | Rural | Total | Anorexia nervosa | Psychiatrist/Neurologist | 5 | 6 | 179.40 | 209.27 | 251.83 | 54.93 | .494 | .692 | 0.50 | 72.43 | [-100.13, 244.99] |
| Total | Total | Rural | Total | Anorexia nervosa | Child and adolescent psychiatrist | 40 | 44 | 77.58 | 115.29 | 74.91 | 96.54 | .909 | .941 | 0.03 | -2.67 | [-48.00, 42.67] |
| Total | Total | Rural | Total | Anorexia nervosa | Psychological psychotherapist | 7 | 12 | 182.43 | 148.92 | 114.58 | 117.42 | .326 | .541 | 0.52 | -67.85 | [-188.48, 52.79] |
| Total | Total | Rural | Total | Anorexia nervosa | Child and adolescent psychotherapist | 67 | 72 | 79.10 | 86.96 | 43.61 | 60.70 | .006 | **.047** | **0.48** | -35.49 | [-60.28, -10.70] |
| Total | Total | Total | Low SES | Anorexia nervosa | General practitioner | 42 | 30 | 79.83 | 139.12 | 107.00 | 137.22 | .413 | .632 | 0.20 | 27.17 | [-37.65, 91.98] |
| Total | Total | Total | Low SES | Anorexia nervosa | Pediatrician | 27 | 29 | 71.22 | 107.45 | 37.97 | 47.40 | .148 | .363 | 0.41 | -33.26 | [-76.24, 9.72] |
| Total | Total | Total | Low SES | Anorexia nervosa | Child and adolescent psychiatrist | 20 | 15 | 102.10 | 137.69 | 67.87 | 115.03 | .429 | .648 | 0.27 | -34.23 | [-120.31, 51.84] |
| Total | Total | Total | Low SES | Anorexia nervosa | Child and adolescent psychotherapist | 25 | 27 | 98.36 | 122.88 | 33.04 | 46.15 | .018 | .092 | 0.71 | -65.32 | [-115.05, -15.60] |
| Total | Total | Total | Medium SES | Anorexia nervosa | General practitioner | 178 | 160 | 93.53 | 128.75 | 68.89 | 86.44 | .038 | .143 | 0.22 | -24.64 | [-48.29, -0.99] |
| Total | Total | Total | Medium SES | Anorexia nervosa | Pediatrician | 130 | 153 | 67.18 | 98.66 | 40.16 | 58.73 | .007 | **.048** | **0.34** | -27.01 | [-45.62, -8.41] |
| Total | Total | Total | Medium SES | Anorexia nervosa | Psychiatrist/Neurologist | 21 | 12 | 200.67 | 153.63 | 166.08 | 114.33 | .468 | .674 | 0.25 | -34.58 | [-134.55, 65.39] |
| Total | Total | Total | Medium SES | Anorexia nervosa | Child and adolescent psychiatrist | 93 | 106 | 99.18 | 128.51 | 67.96 | 91.40 | .053 | .177 | 0.28 | -31.22 | [-61.93, -0.51] |
| Total | Total | Total | Medium SES | Anorexia nervosa | Psychological psychotherapist | 19 | 19 | 107.26 | 99.12 | 132.32 | 131.08 | .511 | .702 | 0.22 | 25.05 | [-48.84, 98.95] |
| Total | Total | Total | Medium SES | Anorexia nervosa | Child and adolescent psychotherapist | 132 | 151 | 71.41 | 101.77 | 51.83 | 69.87 | .064 | .206 | 0.23 | -19.57 | [-39.71, 0.56] |
| Total | Total | Total | High SES | Anorexia nervosa | General practitioner | 71 | 69 | 87.25 | 134.03 | 77.22 | 100.12 | .616 | .765 | 0.08 | -10.04 | [-49.31, 29.24] |
| Total | Total | Total | High SES | Anorexia nervosa | Pediatrician | 55 | 63 | 61.91 | 102.98 | 54.13 | 72.61 | .641 | .777 | 0.09 | -7.78 | [-39.63, 24.07] |
| Total | Total | Total | High SES | Anorexia nervosa | Psychiatrist/Neurologist | 7 | 6 | 105.14 | 80.18 | 133.50 | 161.60 | .708 | .812 | 0.23 | 28.36 | [-106.86, 163.57] |
| Total | Total | Total | High SES | Anorexia nervosa | Child and adolescent psychiatrist | 44 | 45 | 69.55 | 103.47 | 46.27 | 71.17 | .221 | .453 | 0.26 | -23.28 | [-60.10, 13.55] |
| Total | Total | Total | High SES | Anorexia nervosa | Child and adolescent psychotherapist | 45 | 63 | 109.18 | 147.76 | 51.95 | 84.16 | .022 | .102 | 0.50 | -57.23 | [-101.19, -13.26] |
| Female | Total | Total | Total | Anxiety disorder | General practitioner | 1,342 | 848 | 95.90 | 129.51 | 85.60 | 105.66 | .042 | .150 | 0.09 | -10.31 | [-20.70, 0.08] |
| Female | Total | Total | Total | Anxiety disorder | Pediatrician | 1,813 | 1,253 | 52.05 | 86.71 | 43.14 | 69.60 | .002 | .021 | 0.11 | -8.91 | [-14.68, -3.14] |
| Female | Total | Total | Total | Anxiety disorder | Psychiatrist/Neurologist | 135 | 43 | 208.42 | 188.79 | 138.51 | 131.70 | .008 | .052 | 0.40 | -69.91 | [-130.61, -9.21] |
| Female | Total | Total | Total | Anxiety disorder | Child and adolescent psychiatrist | 653 | 510 | 82.93 | 122.56 | 61.76 | 87.69 | .001 | .012 | 0.19 | -21.16 | [-33.75, -8.58] |
| Female | Total | Total | Total | Anxiety disorder | Psychological psychotherapist | 85 | 40 | 117.18 | 153.22 | 94.78 | 117.68 | .371 | .587 | 0.16 | -22.40 | [-76.11, 31.31] |
| Female | Total | Total | Total | Anxiety disorder | Child and adolescent psychotherapist | 714 | 558 | 91.12 | 140.89 | 61.81 | 96.39 | < .001 | **.001** | **0.24** | -29.31 | [-42.97, -15.64] |
| Male | Total | Total | Total | Anxiety disorder | General practitioner | 1,125 | 666 | 110.01 | 136.10 | 105.26 | 123.60 | .450 | .660 | 0.04 | -4.75 | [-17.36, 7.86] |
| Male | Total | Total | Total | Anxiety disorder | Pediatrician | 2,045 | 1,342 | 43.77 | 74.84 | 42.72 | 64.58 | .665 | .786 | 0.01 | -1.05 | [-5.93, 3.84] |
| Male | Total | Total | Total | Anxiety disorder | Psychiatrist/Neurologist | 67 | 41 | 161.64 | 171.38 | 144.12 | 150.28 | .579 | .745 | 0.11 | -17.52 | [-81.15, 46.11] |
| Male | Total | Total | Total | Anxiety disorder | Child and adolescent psychiatrist | 742 | 465 | 87.06 | 125.41 | 65.55 | 98.54 | .001 | .015 | 0.19 | -21.51 | [-34.93, -8.08] |
| Male | Total | Total | Total | Anxiety disorder | Psychological psychotherapist | 52 | 23 | 123.90 | 159.47 | 106.09 | 149.32 | .643 | .777 | 0.11 | -17.82 | [-94.62, 58.99] |
| Male | Total | Total | Total | Anxiety disorder | Child and adolescent psychotherapist | 532 | 353 | 98.29 | 139.85 | 75.45 | 101.76 | .005 | .041 | 0.18 | -22.84 | [-39.80, -5.88] |
| Total | 0-9 | Total | Total | Anxiety disorder | General practitioner | 748 | 374 | 121.47 | 146.82 | 103.45 | 111.48 | .022 | .102 | 0.13 | -18.01 | [-34.90, -1.12] |
| Total | 0-9 | Total | Total | Anxiety disorder | Pediatrician | 2,156 | 1,240 | 39.97 | 68.15 | 34.50 | 55.49 | .011 | .066 | 0.09 | -5.46 | [-9.92, -1.00] |
| Total | 0-9 | Total | Total | Anxiety disorder | Psychiatrist/Neurologist | 32 | 5 | 173.31 | 200.62 | 108.40 | 106.31 | .302 | .521 | 0.34 | -64.91 | [-246.06, 116.24] |
| Total | 0-9 | Total | Total | Anxiety disorder | Child and adolescent psychiatrist | 533 | 300 | 83.69 | 123.59 | 66.93 | 101.08 | .035 | .137 | 0.14 | -16.75 | [-33.16, -0.34] |
| Total | 0-9 | Total | Total | Anxiety disorder | Psychological psychotherapist | 24 | 8 | 117.25 | 169.74 | 95.62 | 129.96 | .712 | .815 | 0.13 | -21.62 | [-150.72, 107.47] |
| Total | 0-9 | Total | Total | Anxiety disorder | Child and adolescent psychotherapist | 381 | 220 | 98.29 | 145.71 | 71.34 | 101.18 | .008 | .052 | 0.21 | -26.95 | [-48.72, -5.17] |
| Total | 10-13 | Total | Total | Anxiety disorder | General practitioner | 810 | 454 | 109.69 | 135.09 | 98.48 | 115.89 | .121 | .312 | 0.09 | -11.21 | [-25.98, 3.56] |
| Total | 10-13 | Total | Total | Anxiety disorder | Pediatrician | 1,127 | 818 | 51.61 | 83.76 | 46.95 | 67.46 | .175 | .397 | 0.06 | -4.66 | [-11.62, 2.30] |
| Total | 10-13 | Total | Total | Anxiety disorder | Psychiatrist/Neurologist | 32 | 17 | 136.62 | 133.04 | 96.59 | 98.04 | .238 | .472 | 0.33 | -40.04 | [-111.95, 31.88] |
| Total | 10-13 | Total | Total | Anxiety disorder | Child and adolescent psychiatrist | 534 | 355 | 85.67 | 125.06 | 59.08 | 82.91 | < .001 | **.005** | **0.24** | -26.59 | [-41.38, -11.80] |
| Total | 10-13 | Total | Total | Anxiety disorder | Psychological psychotherapist | 36 | 22 | 92.83 | 145.33 | 77.27 | 114.68 | .653 | .778 | 0.12 | -15.56 | [-86.98, 55.86] |
| Total | 10-13 | Total | Total | Anxiety disorder | Child and adolescent psychotherapist | 479 | 360 | 95.33 | 139.02 | 66.39 | 100.34 | < .001 | **.009** | **0.23** | -28.94 | [-45.88, -12.00] |
| Total | 14-17 | Total | Total | Anxiety disorder | General practitioner | 909 | 686 | 80.04 | 113.99 | 86.43 | 114.36 | .269 | .496 | 0.06 | 6.38 | [-4.93, 17.70] |
| Total | 14-17 | Total | Total | Anxiety disorder | Pediatrician | 575 | 537 | 68.77 | 109.06 | 56.23 | 85.61 | .033 | .131 | 0.13 | -12.54 | [-24.12, -0.96] |
| Total | 14-17 | Total | Total | Anxiety disorder | Psychiatrist/Neurologist | 138 | 62 | 210.50 | 188.35 | 156.15 | 150.44 | .031 | .127 | 0.31 | -54.35 | [-107.56, -1.15] |
| Total | 14-17 | Total | Total | Anxiety disorder | Child and adolescent psychiatrist | 328 | 320 | 86.59 | 123.53 | 65.40 | 95.72 | .015 | .082 | 0.19 | -21.18 | [-38.23, -4.14] |
| Total | 14-17 | Total | Total | Anxiety disorder | Psychological psychotherapist | 77 | 33 | 133.08 | 155.11 | 114.12 | 139.20 | .530 | .712 | 0.13 | -18.96 | [-80.36, 42.45] |
| Total | 14-17 | Total | Total | Anxiety disorder | Child and adolescent psychotherapist | 386 | 331 | 88.70 | 137.01 | 65.05 | 95.31 | .007 | .048 | 0.20 | -23.65 | [-41.21, -6.09] |
| Total | Total | Urban | Total | Anxiety disorder | General practitioner | 1,571 | 965 | 108.09 | 137.41 | 102.12 | 117.43 | .245 | .476 | 0.05 | -5.97 | [-16.40, 4.47] |
| Total | Total | Urban | Total | Anxiety disorder | Pediatrician | 2,784 | 1,845 | 44.78 | 75.76 | 43.57 | 67.39 | .569 | .739 | 0.02 | -1.21 | [-5.48, 3.06] |
| Total | Total | Urban | Total | Anxiety disorder | Psychiatrist/Neurologist | 127 | 59 | 195.55 | 179.56 | 143.66 | 146.31 | .039 | .143 | 0.31 | -51.89 | [-104.32, 0.54] |
| Total | Total | Urban | Total | Anxiety disorder | Child and adolescent psychiatrist | 1,029 | 735 | 85.96 | 123.45 | 62.50 | 90.92 | < .001 | **< .001** | **0.21** | -23.46 | [-33.97, -12.95] |
| Total | Total | Urban | Total | Anxiety disorder | Psychological psychotherapist | 98 | 42 | 125.80 | 159.53 | 119.10 | 141.52 | .806 | .878 | 0.04 | -6.70 | [-62.51, 49.11] |
| Total | Total | Urban | Total | Anxiety disorder | Child and adolescent psychotherapist | 888 | 655 | 94.19 | 142.75 | 65.45 | 96.50 | < .001 | **< .001** | **0.23** | -28.74 | [-41.38, -16.10] |
| Total | Total | Rural | Total | Anxiety disorder | General practitioner | 896 | 548 | 92.25 | 123.48 | 80.51 | 107.31 | .057 | .187 | 0.10 | -11.75 | [-24.25, 0.75] |
| Total | Total | Rural | Total | Anxiety disorder | Pediatrician | 1,074 | 749 | 55.12 | 92.00 | 41.13 | 66.01 | < .001 | .005 | 0.17 | -13.99 | [-21.67, -6.31] |
| Total | Total | Rural | Total | Anxiety disorder | Psychiatrist/Neurologist | 75 | 25 | 188.43 | 192.70 | 135.56 | 127.43 | .123 | .313 | 0.30 | -52.87 | [-133.86, 28.13] |
| Total | Total | Rural | Total | Anxiety disorder | Child and adolescent psychiatrist | 366 | 240 | 82.80 | 125.89 | 66.87 | 99.20 | .083 | .244 | 0.14 | -15.93 | [-34.82, 2.97] |
| Total | Total | Rural | Total | Anxiety disorder | Psychological psychotherapist | 39 | 21 | 104.49 | 144.10 | 58.52 | 89.44 | .134 | .339 | 0.36 | -45.96 | [-113.82, 21.90] |
| Total | Total | Rural | Total | Anxiety disorder | Child and adolescent psychotherapist | 358 | 255 | 94.15 | 134.70 | 71.57 | 104.21 | .020 | .096 | 0.18 | -22.57 | [-42.32, -2.83] |
| Total | Total | Total | Low SES | Anxiety disorder | General practitioner | 385 | 226 | 99.87 | 120.31 | 104.88 | 123.64 | .626 | .772 | 0.04 | 5.00 | [-14.96, 24.97] |
| Total | Total | Total | Low SES | Anxiety disorder | Pediatrician | 635 | 428 | 48.74 | 78.61 | 41.43 | 67.17 | .105 | .285 | 0.10 | -7.31 | [-16.41, 1.79] |
| Total | Total | Total | Low SES | Anxiety disorder | Psychiatrist/Neurologist | 25 | 8 | 235.00 | 227.14 | 121.00 | 150.30 | .120 | .312 | 0.54 | -114.00 | [-282.98, 54.98] |
| Total | Total | Total | Low SES | Anxiety disorder | Child and adolescent psychiatrist | 207 | 150 | 86.08 | 126.38 | 59.95 | 94.67 | .026 | .115 | 0.23 | -26.14 | [-50.13, -2.14] |
| Total | Total | Total | Low SES | Anxiety disorder | Psychological psychotherapist | 18 | 6 | 94.44 | 99.02 | 100.50 | 63.47 | .865 | .915 | 0.07 | 6.06 | [-79.09, 91.20] |
| Total | Total | Total | Low SES | Anxiety disorder | Child and adolescent psychotherapist | 189 | 159 | 79.96 | 103.33 | 66.62 | 97.90 | .218 | .450 | 0.13 | -13.35 | [-34.63, 7.93] |
| Total | Total | Total | Medium SES | Anxiety disorder | General practitioner | 1,575 | 991 | 104.12 | 134.89 | 89.90 | 110.70 | .004 | .034 | 0.11 | -14.22 | [-24.24, -4.20] |
| Total | Total | Total | Medium SES | Anxiety disorder | Pediatrician | 2,425 | 1,627 | 47.55 | 82.20 | 43.29 | 65.12 | .067 | .210 | 0.06 | -4.26 | [-9.02, 0.50] |
| Total | Total | Total | Medium SES | Anxiety disorder | Psychiatrist/Neurologist | 135 | 57 | 180.84 | 174.85 | 134.61 | 135.30 | .050 | .173 | 0.28 | -46.22 | [-97.06, 4.61] |
| Total | Total | Total | Medium SES | Anxiety disorder | Child and adolescent psychiatrist | 903 | 648 | 83.13 | 121.34 | 64.05 | 93.08 | < .001 | .009 | 0.17 | -19.08 | [-30.22, -7.94] |
| Total | Total | Total | Medium SES | Anxiety disorder | Psychological psychotherapist | 90 | 41 | 120.40 | 155.09 | 103.56 | 146.27 | .551 | .727 | 0.11 | -16.84 | [-73.12, 39.44] |
| Total | Total | Total | Medium SES | Anxiety disorder | Child and adolescent psychotherapist | 783 | 578 | 103.11 | 149.24 | 67.25 | 100.37 | < .001 | **< .001** | **0.27** | -35.87 | [-49.92, -21.82] |
| Total | Total | Total | High SES | Anxiety disorder | General practitioner | 507 | 296 | 98.65 | 135.01 | 100.89 | 118.27 | .806 | .878 | 0.02 | 2.24 | [-16.27, 20.75] |
| Total | Total | Total | High SES | Anxiety disorder | Pediatrician | 798 | 539 | 47.12 | 77.93 | 42.72 | 72.31 | .290 | .513 | 0.06 | -4.41 | [-12.68, 3.87] |
| Total | Total | Total | High SES | Anxiety disorder | Psychiatrist/Neurologist | 42 | 19 | 206.64 | 185.16 | 169.68 | 154.23 | .421 | .639 | 0.21 | -36.96 | [-132.50, 58.58] |
| Total | Total | Total | High SES | Anxiety disorder | Child and adolescent psychiatrist | 285 | 177 | 90.76 | 130.95 | 64.89 | 91.71 | .013 | .075 | 0.22 | -25.87 | [-47.90, -3.83] |
| Total | Total | Total | High SES | Anxiety disorder | Psychological psychotherapist | 29 | 16 | 133.34 | 183.83 | 86.38 | 101.05 | .275 | .503 | 0.29 | -46.97 | [-144.57, 50.63] |
| Total | Total | Total | High SES | Anxiety disorder | Child and adolescent psychotherapist | 274 | 173 | 78.45 | 134.80 | 67.41 | 94.27 | .309 | .527 | 0.09 | -11.04 | [-34.03, 11.94] |
| Female | Total | Total | Total | Depressive disorder | General practitioner | 1,711 | 1,240 | 82.33 | 122.08 | 73.83 | 99.32 | .037 | .143 | 0.08 | -8.50 | [-16.76, -0.23] |
| Female | Total | Total | Total | Depressive disorder | Pediatrician | 964 | 901 | 76.98 | 108.40 | 60.13 | 82.04 | < .001 | .005 | 0.17 | -16.85 | [-25.62, -8.08] |
| Female | Total | Total | Total | Depressive disorder | Psychiatrist/Neurologist | 251 | 156 | 197.06 | 185.30 | 142.92 | 136.57 | .001 | **.013** | **0.32** | -54.14 | [-87.78, -20.51] |
| Female | Total | Total | Total | Depressive disorder | Child and adolescent psychiatrist | 822 | 758 | 85.11 | 126.81 | 64.38 | 95.16 | < .001 | .006 | 0.18 | -20.72 | [-31.85, -9.59] |
| Female | Total | Total | Total | Depressive disorder | Psychological psychotherapist | 150 | 92 | 144.89 | 163.54 | 98.07 | 129.87 | .015 | .082 | 0.31 | -46.83 | [-86.19, -7.47] |
| Female | Total | Total | Total | Depressive disorder | Child and adolescent psychotherapist | 923 | 852 | 78.15 | 124.06 | 59.31 | 86.91 | < .001 | .005 | 0.17 | -18.84 | [-28.88, -8.80] |
| Male | Total | Total | Total | Depressive disorder | General practitioner | 723 | 388 | 84.59 | 120.12 | 73.28 | 102.50 | .099 | .273 | 0.10 | -11.31 | [-25.41, 2.78] |
| Male | Total | Total | Total | Depressive disorder | Pediatrician | 443 | 281 | 70.06 | 105.87 | 60.01 | 76.39 | .139 | .347 | 0.11 | -10.05 | [-24.33, 4.23] |
| Male | Total | Total | Total | Depressive disorder | Psychiatrist/Neurologist | 107 | 54 | 186.83 | 192.15 | 154.93 | 140.23 | .233 | .467 | 0.18 | -31.91 | [-89.67, 25.86] |
| Male | Total | Total | Total | Depressive disorder | Child and adolescent psychiatrist | 307 | 198 | 90.10 | 134.80 | 50.32 | 77.32 | < .001 | **.002** | **0.34** | -39.78 | [-60.46, -19.10] |
| Male | Total | Total | Total | Depressive disorder | Psychological psychotherapist | 52 | 35 | 178.42 | 188.20 | 76.37 | 106.44 | .002 | **.023** | **0.64** | -102.05 | [-170.86, -33.24] |
| Male | Total | Total | Total | Depressive disorder | Child and adolescent psychotherapist | 280 | 190 | 90.75 | 138.25 | 49.23 | 81.55 | < .001 | **.002** | **0.35** | -41.52 | [-63.38, -19.66] |
| Total | 0-9 | Total | Total | Depressive disorder | General practitioner | 58 | 17 | 97.93 | 141.10 | 46.88 | 38.33 | .016 | .086 | 0.41 | -51.05 | [-119.14, 17.04] |
| Total | 0-9 | Total | Total | Depressive disorder | Pediatrician | 102 | 55 | 68.52 | 95.31 | 59.53 | 75.96 | .520 | .709 | 0.10 | -8.99 | [-38.19, 20.21] |
| Total | 0-9 | Total | Total | Depressive disorder | Child and adolescent psychiatrist | 38 | 15 | 92.39 | 120.45 | 79.13 | 110.74 | .705 | .812 | 0.11 | -13.26 | [-83.71, 57.18] |
| Total | 0-9 | Total | Total | Depressive disorder | Child and adolescent psychotherapist | 23 | 13 | 134.39 | 187.48 | 39.92 | 48.16 | .031 | .126 | 0.62 | -94.47 | [-198.86, 9.93] |
| Total | 10-13 | Total | Total | Depressive disorder | General practitioner | 518 | 336 | 95.20 | 125.35 | 84.14 | 106.87 | .168 | .389 | 0.09 | -11.06 | [-27.32, 5.20] |
| Total | 10-13 | Total | Total | Depressive disorder | Pediatrician | 523 | 431 | 72.19 | 101.92 | 56.32 | 73.21 | .005 | .042 | 0.18 | -15.87 | [-27.35, -4.38] |
| Total | 10-13 | Total | Total | Depressive disorder | Psychiatrist/Neurologist | 19 | 14 | 234.05 | 170.14 | 133.71 | 131.30 | .066 | .208 | 0.65 | -100.34 | [-207.37, 6.69] |
| Total | 10-13 | Total | Total | Depressive disorder | Child and adolescent psychiatrist | 333 | 258 | 96.38 | 143.52 | 59.47 | 86.13 | < .001 | **.004** | **0.30** | -36.92 | [-56.72, -17.11] |
| Total | 10-13 | Total | Total | Depressive disorder | Psychological psychotherapist | 27 | 14 | 104.74 | 116.04 | 60.29 | 83.34 | .168 | .389 | 0.42 | -44.46 | [-113.05, 24.14] |
| Total | 10-13 | Total | Total | Depressive disorder | Child and adolescent psychotherapist | 345 | 301 | 85.81 | 133.32 | 59.93 | 81.85 | .003 | **.027** | **0.23** | -25.88 | [-43.24, -8.52] |
| Total | 14-17 | Total | Total | Depressive disorder | General practitioner | 1,858 | 1,275 | 79.13 | 119.53 | 71.31 | 98.59 | .046 | .161 | 0.07 | -7.83 | [-15.77, 0.12] |
| Total | 14-17 | Total | Total | Depressive disorder | Pediatrician | 782 | 696 | 77.37 | 112.77 | 62.49 | 85.36 | .004 | .036 | 0.15 | -14.89 | [-25.18, -4.59] |
| Total | 14-17 | Total | Total | Depressive disorder | Psychiatrist/Neurologist | 338 | 196 | 192.20 | 188.15 | 146.88 | 137.98 | .002 | **.020** | **0.26** | -45.32 | [-75.49, -15.14] |
| Total | 14-17 | Total | Total | Depressive disorder | Child and adolescent psychiatrist | 758 | 683 | 81.81 | 122.41 | 61.84 | 93.64 | < .001 | .009 | 0.18 | -19.97 | [-31.31, -8.62] |
| Total | 14-17 | Total | Total | Depressive disorder | Psychological psychotherapist | 175 | 113 | 161.05 | 176.34 | 96.03 | 127.68 | < .001 | **.008** | **0.41** | -65.02 | [-102.65, -27.40] |
| Total | 14-17 | Total | Total | Depressive disorder | Child and adolescent psychotherapist | 835 | 728 | 77.66 | 122.85 | 56.76 | 88.21 | < .001 | .004 | 0.19 | -20.89 | [-31.64, -10.15] |
| Total | Total | Urban | Total | Depressive disorder | General practitioner | 1,677 | 1,096 | 89.18 | 126.65 | 75.80 | 101.10 | .002 | .024 | 0.11 | -13.38 | [-22.31, -4.46] |
| Total | Total | Urban | Total | Depressive disorder | Pediatrician | 1,027 | 863 | 72.60 | 105.62 | 58.87 | 78.95 | .001 | .017 | 0.15 | -13.73 | [-22.27, -5.19] |
| Total | Total | Urban | Total | Depressive disorder | Psychiatrist/Neurologist | 263 | 144 | 193.19 | 191.35 | 139.17 | 134.78 | .001 | **.016** | **0.31** | -54.02 | [-89.27, -18.77] |
| Total | Total | Urban | Total | Depressive disorder | Child and adolescent psychiatrist | 836 | 691 | 85.33 | 129.03 | 59.16 | 90.02 | < .001 | **< .001** | **0.23** | -26.17 | [-37.57, -14.78] |
| Total | Total | Urban | Total | Depressive disorder | Psychological psychotherapist | 142 | 91 | 154.13 | 176.42 | 82.63 | 110.69 | < .001 | **.005** | **0.46** | -71.50 | [-112.08, -30.92] |
| Total | Total | Urban | Total | Depressive disorder | Child and adolescent psychotherapist | 854 | 734 | 75.44 | 120.20 | 56.17 | 85.11 | < .001 | .005 | 0.18 | -19.27 | [-29.67, -8.86] |
| Total | Total | Rural | Total | Depressive disorder | General practitioner | 755 | 532 | 69.41 | 108.10 | 69.37 | 97.82 | .995 | .998 | 0.00 | -0.04 | [-11.57, 11.50] |
| Total | Total | Rural | Total | Depressive disorder | Pediatrician | 380 | 319 | 80.74 | 112.78 | 63.41 | 85.30 | .021 | .099 | 0.17 | -17.33 | [-32.38, -2.27] |
| Total | Total | Rural | Total | Depressive disorder | Psychiatrist/Neurologist | 95 | 66 | 196.26 | 176.00 | 160.92 | 142.50 | .162 | .381 | 0.22 | -35.34 | [-86.58, 15.90] |
| Total | Total | Rural | Total | Depressive disorder | Child and adolescent psychiatrist | 293 | 265 | 89.70 | 129.06 | 67.51 | 96.51 | .021 | .099 | 0.19 | -22.19 | [-41.26, -3.12] |
| Total | Total | Rural | Total | Depressive disorder | Psychological psychotherapist | 60 | 36 | 152.10 | 156.56 | 116.00 | 151.24 | .267 | .496 | 0.23 | -36.10 | [-99.98, 27.78] |
| Total | Total | Rural | Total | Depressive disorder | Child and adolescent psychotherapist | 349 | 308 | 94.88 | 143.23 | 60.56 | 88.16 | < .001 | **.005** | **0.28** | -34.32 | [-52.80, -15.85] |
| Total | Total | Total | Low SES | Depressive disorder | General practitioner | 331 | 215 | 85.95 | 119.08 | 84.84 | 113.80 | .912 | .942 | 0.01 | -1.12 | [-21.21, 18.97] |
| Total | Total | Total | Low SES | Depressive disorder | Pediatrician | 212 | 164 | 77.83 | 112.50 | 56.23 | 70.59 | .023 | .106 | 0.22 | -21.60 | [-41.27, -1.93] |
| Total | Total | Total | Low SES | Depressive disorder | Psychiatrist/Neurologist | 35 | 30 | 247.03 | 192.37 | 175.27 | 149.61 | .096 | .270 | 0.41 | -71.76 | [-156.61, 13.09] |
| Total | Total | Total | Low SES | Depressive disorder | Child and adolescent psychiatrist | 138 | 108 | 86.43 | 131.32 | 60.93 | 93.26 | .076 | .227 | 0.22 | -25.51 | [-54.76, 3.74] |
| Total | Total | Total | Low SES | Depressive disorder | Psychological psychotherapist | 16 | 15 | 153.31 | 167.33 | 97.07 | 139.56 | .317 | .533 | 0.36 | -56.25 | [-165.11, 52.62] |
| Total | Total | Total | Low SES | Depressive disorder | Child and adolescent psychotherapist | 165 | 135 | 91.68 | 130.17 | 59.04 | 78.85 | .008 | .052 | 0.30 | -32.64 | [-57.68, -7.60] |
| Total | Total | Total | Medium SES | Depressive disorder | General practitioner | 1,534 | 1,009 | 82.97 | 122.42 | 70.59 | 99.52 | .005 | .041 | 0.11 | -12.38 | [-21.43, -3.33] |
| Total | Total | Total | Medium SES | Depressive disorder | Pediatrician | 899 | 730 | 76.61 | 111.58 | 60.18 | 82.52 | .001 | .012 | 0.17 | -16.44 | [-26.16, -6.71] |
| Total | Total | Total | Medium SES | Depressive disorder | Psychiatrist/Neurologist | 225 | 126 | 201.34 | 191.74 | 140.90 | 138.34 | .001 | **.012** | **0.35** | -60.44 | [-98.50, -22.38] |
| Total | Total | Total | Medium SES | Depressive disorder | Child and adolescent psychiatrist | 693 | 600 | 92.72 | 133.90 | 63.45 | 93.31 | < .001 | **< .001** | **0.25** | -29.27 | [-42.04, -16.50] |
| Total | Total | Total | Medium SES | Depressive disorder | Psychological psychotherapist | 127 | 72 | 160.05 | 162.80 | 109.01 | 136.31 | .019 | .095 | 0.33 | -51.03 | [-95.50, -6.57] |
| Total | Total | Total | Medium SES | Depressive disorder | Child and adolescent psychotherapist | 765 | 656 | 81.96 | 130.24 | 58.74 | 88.95 | < .001 | **.003** | **0.21** | -23.22 | [-35.01, -11.43] |
| Total | Total | Total | High SES | Depressive disorder | General practitioner | 567 | 404 | 81.54 | 120.67 | 75.53 | 93.14 | .382 | .600 | 0.05 | -6.01 | [-20.06, 8.03] |
| Total | Total | Total | High SES | Depressive disorder | Pediatrician | 296 | 288 | 67.14 | 90.44 | 62.11 | 81.60 | .481 | .685 | 0.06 | -5.02 | [-19.01, 8.96] |
| Total | Total | Total | High SES | Depressive disorder | Psychiatrist/Neurologist | 98 | 54 | 158.22 | 169.18 | 141.67 | 127.94 | .498 | .695 | 0.11 | -16.56 | [-68.33, 35.21] |
| Total | Total | Total | High SES | Depressive disorder | Child and adolescent psychiatrist | 298 | 248 | 71.92 | 114.74 | 56.92 | 87.94 | .084 | .246 | 0.15 | -15.00 | [-32.43, 2.42] |
| Total | Total | Total | High SES | Depressive disorder | Psychological psychotherapist | 59 | 40 | 139.54 | 188.39 | 59.75 | 84.31 | .005 | **.042** | **0.51** | -79.79 | [-142.08, -17.50] |
| Total | Total | Total | High SES | Depressive disorder | Child and adolescent psychotherapist | 273 | 251 | 72.22 | 117.87 | 53.31 | 81.96 | .032 | .131 | 0.18 | -18.91 | [-36.44, -1.38] |
| Female | Total | Total | Total | OCD | General practitioner | 52 | 41 | 100.60 | 153.79 | 74.63 | 88.62 | .310 | .527 | 0.20 | -25.96 | [-78.87, 26.95] |
| Female | Total | Total | Total | OCD | Pediatrician | 42 | 47 | 62.02 | 78.99 | 38.94 | 44.90 | .100 | .274 | 0.36 | -23.09 | [-49.43, 3.25] |
| Female | Total | Total | Total | OCD | Psychiatrist/Neurologist | 10 | 8 | 181.00 | 180.97 | 192.25 | 111.61 | .874 | .921 | 0.07 | 11.25 | [-132.40, 154.90] |
| Female | Total | Total | Total | OCD | Child and adolescent psychiatrist | 26 | 28 | 88.73 | 126.86 | 56.89 | 74.38 | .272 | .499 | 0.31 | -31.84 | [-86.82, 23.14] |
| Female | Total | Total | Total | OCD | Child and adolescent psychotherapist | 37 | 36 | 55.59 | 103.88 | 46.14 | 63.04 | .639 | .777 | 0.11 | -9.46 | [-49.01, 30.10] |
| Male | Total | Total | Total | OCD | General practitioner | 45 | 30 | 100.20 | 133.08 | 108.10 | 131.88 | .801 | .876 | 0.06 | 7.90 | [-53.36, 69.16] |
| Male | Total | Total | Total | OCD | Pediatrician | 48 | 36 | 68.06 | 98.89 | 53.03 | 65.12 | .404 | .622 | 0.17 | -15.03 | [-52.25, 22.18] |
| Male | Total | Total | Total | OCD | Child and adolescent psychiatrist | 33 | 20 | 39.36 | 48.75 | 30.30 | 40.96 | .472 | .678 | 0.20 | -9.06 | [-34.62, 16.49] |
| Male | Total | Total | Total | OCD | Child and adolescent psychotherapist | 29 | 18 | 98.62 | 139.09 | 101.28 | 102.59 | .940 | .962 | 0.02 | 2.66 | [-71.77, 77.08] |
| Total | 0-9 | Total | Total | OCD | General practitioner | 10 | 9 | 123.80 | 166.14 | 118.33 | 130.98 | .937 | .960 | 0.04 | -5.47 | [-141.11, 130.18] |
| Total | 0-9 | Total | Total | OCD | Pediatrician | 24 | 27 | 75.71 | 66.12 | 35.85 | 40.37 | .015 | .082 | 0.74 | -39.86 | [-69.55, -10.16] |
| Total | 10-13 | Total | Total | OCD | General practitioner | 35 | 9 | 71.03 | 91.23 | 61.22 | 52.44 | .678 | .790 | 0.12 | -9.81 | [-72.23, 52.62] |
| Total | 10-13 | Total | Total | OCD | Pediatrician | 37 | 20 | 35.08 | 67.44 | 52.05 | 53.30 | .303 | .521 | 0.27 | 16.97 | [-17.25, 51.19] |
| Total | 10-13 | Total | Total | OCD | Child and adolescent psychiatrist | 29 | 11 | 52.69 | 107.02 | 38.09 | 43.02 | .542 | .720 | 0.15 | -14.60 | [-80.17, 50.97] |
| Total | 10-13 | Total | Total | OCD | Child and adolescent psychotherapist | 34 | 13 | 81.03 | 127.34 | 65.85 | 85.47 | .641 | .777 | 0.13 | -15.18 | [-90.37, 60.00] |
| Total | 14-17 | Total | Total | OCD | General practitioner | 52 | 53 | 115.69 | 166.05 | 88.43 | 113.00 | .329 | .544 | 0.19 | -27.26 | [-81.50, 26.98] |
| Total | 14-17 | Total | Total | OCD | Pediatrician | 29 | 36 | 95.07 | 117.99 | 48.06 | 64.43 | .061 | .197 | 0.51 | -47.01 | [-92.09, -1.94] |
| Total | 14-17 | Total | Total | OCD | Psychiatrist/Neurologist | 9 | 10 | 162.56 | 203.24 | 164.50 | 112.82 | .980 | .986 | 0.01 | 1.94 | [-143.76, 147.65] |
| Total | 14-17 | Total | Total | OCD | Child and adolescent psychiatrist | 28 | 34 | 72.61 | 82.61 | 52.00 | 70.44 | .301 | .521 | 0.27 | -20.61 | [-58.70, 17.49] |
| Total | 14-17 | Total | Total | OCD | Child and adolescent psychotherapist | 28 | 37 | 65.50 | 121.70 | 56.57 | 78.26 | .736 | .829 | 0.09 | -8.93 | [-57.65, 39.79] |
| Total | Total | Urban | Total | OCD | General practitioner | 64 | 40 | 118.38 | 165.95 | 93.88 | 123.95 | .393 | .610 | 0.16 | -24.50 | [-84.26, 35.26] |
| Total | Total | Urban | Total | OCD | Pediatrician | 65 | 62 | 71.89 | 96.75 | 46.13 | 58.39 | .071 | .216 | 0.32 | -25.76 | [-53.72, 2.19] |
| Total | Total | Urban | Total | OCD | Psychiatrist/Neurologist | 10 | 8 | 193.10 | 184.77 | 161.62 | 124.91 | .673 | .788 | 0.20 | -31.47 | [-181.47, 118.52] |
| Total | Total | Urban | Total | OCD | Child and adolescent psychiatrist | 40 | 34 | 66.72 | 104.56 | 47.85 | 62.62 | .342 | .553 | 0.21 | -18.87 | [-59.04, 21.30] |
| Total | Total | Urban | Total | OCD | Child and adolescent psychotherapist | 39 | 37 | 89.46 | 146.51 | 67.92 | 90.13 | .440 | .653 | 0.18 | -21.54 | [-76.59, 33.50] |
| Total | Total | Rural | Total | OCD | General practitioner | 33 | 31 | 65.58 | 76.99 | 82.19 | 88.77 | .428 | .647 | 0.20 | 16.62 | [-24.02, 57.26] |
| Total | Total | Rural | Total | OCD | Pediatrician | 25 | 21 | 47.96 | 66.69 | 41.86 | 42.98 | .710 | .814 | 0.11 | -6.10 | [-39.26, 27.05] |
| Total | Total | Rural | Total | OCD | Child and adolescent psychiatrist | 19 | 14 | 49.32 | 68.11 | 40.86 | 67.71 | .726 | .824 | 0.12 | -8.46 | [-55.36, 38.45] |
| Total | Total | Rural | Total | OCD | Child and adolescent psychotherapist | 27 | 17 | 52.89 | 68.87 | 57.12 | 61.58 | .833 | .897 | 0.06 | 4.23 | [-35.94, 44.39] |
| Total | Total | Total | Low SES | OCD | General practitioner | 12 | 14 | 157.83 | 167.49 | 119.07 | 142.90 | .536 | .714 | 0.25 | -38.76 | [-158.01, 80.49] |
| Total | Total | Total | Low SES | OCD | Pediatrician | 13 | 13 | 106.15 | 127.07 | 23.92 | 26.33 | .040 | .146 | 0.90 | -82.23 | [-152.77, -11.69] |
| Total | Total | Total | Low SES | OCD | Child and adolescent psychiatrist | 7 | 9 | 107.43 | 103.82 | 32.33 | 26.09 | .111 | .296 | 1.06 | -75.10 | [-145.00, -5.19] |
| Total | Total | Total | Low SES | OCD | Child and adolescent psychotherapist | 9 | 7 | 83.33 | 93.84 | 42.14 | 40.72 | .262 | .494 | 0.54 | -41.19 | [-116.04, 33.66] |
| Total | Total | Total | Medium SES | OCD | General practitioner | 61 | 36 | 91.13 | 137.13 | 77.58 | 105.29 | .587 | .748 | 0.11 | -13.55 | [-65.59, 38.49] |
| Total | Total | Total | Medium SES | OCD | Pediatrician | 64 | 53 | 52.67 | 81.56 | 56.09 | 59.65 | .794 | .872 | 0.05 | 3.42 | [-22.96, 29.81] |
| Total | Total | Total | Medium SES | OCD | Psychiatrist/Neurologist | 8 | 8 | 180.50 | 203.74 | 167.50 | 96.81 | .874 | .921 | 0.08 | -13.00 | [-169.31, 143.31] |
| Total | Total | Total | Medium SES | OCD | Child and adolescent psychiatrist | 34 | 25 | 51.18 | 101.84 | 57.12 | 66.89 | .788 | .867 | 0.07 | 5.94 | [-39.92, 51.81] |
| Total | Total | Total | Medium SES | OCD | Child and adolescent psychotherapist | 38 | 33 | 76.71 | 128.79 | 59.52 | 79.81 | .496 | .693 | 0.16 | -17.20 | [-67.96, 33.57] |
| Total | Total | Total | High SES | OCD | General practitioner | 24 | 21 | 95.29 | 147.90 | 87.76 | 91.40 | .836 | .898 | 0.06 | -7.53 | [-80.65, 65.59] |
| Total | Total | Total | High SES | OCD | Pediatrician | 13 | 17 | 86.23 | 74.03 | 26.76 | 46.03 | .020 | .097 | 1.00 | -59.47 | [-102.55, -16.38] |
| Total | Total | Total | High SES | OCD | Child and adolescent psychiatrist | 18 | 14 | 61.89 | 72.15 | 34.29 | 73.36 | .297 | .519 | 0.38 | -27.60 | [-78.37, 23.16] |
| Total | Total | Total | High SES | OCD | Child and adolescent psychotherapist | 19 | 14 | 65.89 | 123.35 | 87.50 | 99.61 | .582 | .747 | 0.19 | 21.61 | [-57.09, 100.30] |
| Female | Total | Total | Total | PTSD | General practitioner | 260 | 162 | 93.82 | 123.33 | 70.54 | 104.21 | .038 | .143 | 0.20 | -23.28 | [-46.11, -0.45] |
| Female | Total | Total | Total | PTSD | Pediatrician | 259 | 154 | 51.18 | 89.02 | 40.17 | 55.29 | .122 | .312 | 0.14 | -11.01 | [-26.61, 4.58] |
| Female | Total | Total | Total | PTSD | Psychiatrist/Neurologist | 25 | 15 | 272.44 | 215.54 | 99.07 | 123.56 | .003 | .026 | 0.93 | -173.37 | [-293.07, -53.68] |
| Female | Total | Total | Total | PTSD | Child and adolescent psychiatrist | 87 | 58 | 84.11 | 121.91 | 55.83 | 81.93 | .097 | .271 | 0.26 | -28.29 | [-64.09, 7.52] |
| Female | Total | Total | Total | PTSD | Psychological psychotherapist | 17 | 9 | 182.35 | 192.47 | 115.00 | 105.53 | .261 | .493 | 0.40 | -67.35 | [-203.54, 68.83] |
| Female | Total | Total | Total | PTSD | Child and adolescent psychotherapist | 125 | 77 | 71.52 | 102.68 | 54.68 | 83.57 | .205 | .434 | 0.18 | -16.84 | [-44.07, 10.38] |
| Male | Total | Total | Total | PTSD | General practitioner | 124 | 80 | 96.15 | 135.02 | 110.64 | 139.61 | .464 | .672 | 0.11 | 14.49 | [-23.97, 52.95] |
| Male | Total | Total | Total | PTSD | Pediatrician | 205 | 121 | 47.11 | 76.10 | 45.26 | 73.35 | .828 | .896 | 0.02 | -1.85 | [-18.73, 15.02] |
| Male | Total | Total | Total | PTSD | Psychiatrist/Neurologist | 6 | 6 | 236.00 | 302.12 | 131.83 | 99.86 | .453 | .664 | 0.46 | -104.17 | [-358.77, 150.44] |
| Male | Total | Total | Total | PTSD | Child and adolescent psychiatrist | 47 | 43 | 95.72 | 139.13 | 92.88 | 117.12 | .917 | .943 | 0.02 | -2.84 | [-56.24, 50.56] |
| Male | Total | Total | Total | PTSD | Child and adolescent psychotherapist | 58 | 33 | 86.14 | 149.62 | 55.30 | 68.55 | .183 | .410 | 0.24 | -30.83 | [-84.94, 23.27] |
| Total | 0-9 | Total | Total | PTSD | General practitioner | 86 | 37 | 107.01 | 142.53 | 141.32 | 148.91 | .240 | .473 | 0.24 | 34.31 | [-21.35, 89.98] |
| Total | 0-9 | Total | Total | PTSD | Pediatrician | 236 | 119 | 35.00 | 55.02 | 31.87 | 51.16 | .597 | .753 | 0.06 | -3.13 | [-14.97, 8.72] |
| Total | 0-9 | Total | Total | PTSD | Child and adolescent psychiatrist | 25 | 22 | 77.60 | 110.40 | 63.68 | 100.22 | .653 | .778 | 0.13 | -13.92 | [-74.52, 46.68] |
| Total | 0-9 | Total | Total | PTSD | Child and adolescent psychotherapist | 52 | 20 | 70.33 | 130.49 | 66.75 | 67.52 | .880 | .924 | 0.03 | -3.58 | [-63.81, 56.66] |
| Total | 10-13 | Total | Total | PTSD | General practitioner | 106 | 60 | 113.70 | 137.98 | 114.57 | 135.91 | .969 | .979 | 0.01 | 0.87 | [-42.59, 44.33] |
| Total | 10-13 | Total | Total | PTSD | Pediatrician | 134 | 81 | 56.73 | 78.91 | 46.99 | 70.75 | .350 | .561 | 0.13 | -9.74 | [-30.69, 11.21] |
| Total | 10-13 | Total | Total | PTSD | Child and adolescent psychiatrist | 51 | 29 | 96.31 | 147.38 | 99.62 | 120.46 | .914 | .942 | 0.02 | 3.31 | [-59.75, 66.36] |
| Total | 10-13 | Total | Total | PTSD | Child and adolescent psychotherapist | 59 | 35 | 62.83 | 94.57 | 47.03 | 64.42 | .339 | .551 | 0.19 | -15.80 | [-51.22, 19.61] |
| Total | 14-17 | Total | Total | PTSD | General practitioner | 192 | 145 | 78.44 | 111.03 | 56.38 | 91.21 | .046 | .163 | 0.21 | -22.06 | [-44.27, 0.15] |
| Total | 14-17 | Total | Total | PTSD | Pediatrician | 94 | 75 | 75.01 | 129.95 | 54.17 | 71.72 | .188 | .410 | 0.19 | -20.84 | [-53.64, 11.96] |
| Total | 14-17 | Total | Total | PTSD | Psychiatrist/Neurologist | 27 | 17 | 276.26 | 243.61 | 115.29 | 118.81 | .006 | **.043** | **0.78** | -160.97 | [-285.50, -36.43] |
| Total | 14-17 | Total | Total | PTSD | Child and adolescent psychiatrist | 58 | 50 | 85.60 | 117.55 | 58.84 | 83.69 | .172 | .393 | 0.26 | -26.76 | [-65.83, 12.30] |
| Total | 14-17 | Total | Total | PTSD | Psychological psychotherapist | 16 | 8 | 178.69 | 196.27 | 140.50 | 180.59 | .642 | .777 | 0.20 | -38.19 | [-200.64, 124.27] |
| Total | 14-17 | Total | Total | PTSD | Child and adolescent psychotherapist | 72 | 55 | 91.28 | 128.71 | 55.53 | 91.13 | .069 | .215 | 0.31 | -35.75 | [-75.77, 4.27] |
| Total | Total | Urban | Total | PTSD | General practitioner | 219 | 159 | 98.62 | 127.81 | 85.79 | 115.29 | .308 | .527 | 0.10 | -12.83 | [-37.89, 12.23] |
| Total | Total | Urban | Total | PTSD | Pediatrician | 295 | 188 | 47.39 | 76.77 | 40.55 | 61.70 | .282 | .510 | 0.10 | -6.84 | [-19.88, 6.20] |
| Total | Total | Urban | Total | PTSD | Psychiatrist/Neurologist | 16 | 15 | 265.38 | 219.30 | 123.07 | 122.50 | .034 | .137 | 0.79 | -142.31 | [-268.56, -16.06] |
| Total | Total | Urban | Total | PTSD | Child and adolescent psychiatrist | 85 | 69 | 93.41 | 136.48 | 67.90 | 100.73 | .184 | .410 | 0.21 | -25.51 | [-64.20, 13.17] |
| Total | Total | Urban | Total | PTSD | Psychological psychotherapist | 16 | 9 | 153.25 | 127.09 | 153.11 | 173.11 | .998 | .998 | 0.00 | -0.14 | [-118.36, 118.09] |
| Total | Total | Urban | Total | PTSD | Child and adolescent psychotherapist | 104 | 66 | 68.14 | 108.52 | 48.33 | 72.90 | .157 | .372 | 0.21 | -19.81 | [-49.52, 9.90] |
| Total | Total | Rural | Total | PTSD | General practitioner | 165 | 83 | 89.19 | 126.23 | 79.96 | 124.58 | .584 | .748 | 0.07 | -9.23 | [-42.38, 23.92] |
| Total | Total | Rural | Total | PTSD | Pediatrician | 169 | 87 | 52.86 | 94.24 | 46.41 | 68.31 | .532 | .713 | 0.07 | -6.44 | [-28.77, 15.88] |
| Total | Total | Rural | Total | PTSD | Psychiatrist/Neurologist | 15 | 6 | 265.40 | 247.32 | 71.83 | 96.81 | .019 | .095 | 0.89 | -193.57 | [-399.99, 12.85] |
| Total | Total | Rural | Total | PTSD | Child and adolescent psychiatrist | 49 | 32 | 79.12 | 111.95 | 79.59 | 98.32 | .984 | .989 | 0.00 | 0.47 | [-47.11, 48.05] |
| Total | Total | Rural | Total | PTSD | Child and adolescent psychotherapist | 79 | 44 | 86.70 | 132.22 | 64.66 | 87.43 | .270 | .496 | 0.19 | -22.04 | [-65.64, 21.57] |
| Total | Total | Total | Low SES | PTSD | General practitioner | 84 | 49 | 84.13 | 106.07 | 117.33 | 149.91 | .177 | .399 | 0.27 | 33.20 | [-10.47, 76.86] |
| Total | Total | Total | Low SES | PTSD | Pediatrician | 94 | 71 | 45.43 | 87.37 | 40.25 | 69.92 | .673 | .788 | 0.06 | -5.17 | [-29.93, 19.59] |
| Total | Total | Total | Low SES | PTSD | Psychiatrist/Neurologist | 7 | 6 | 230.71 | 140.68 | 88.83 | 82.53 | .051 | .173 | 1.20 | -141.88 | [-270.40, -13.36] |
| Total | Total | Total | Low SES | PTSD | Child and adolescent psychiatrist | 32 | 21 | 109.56 | 138.30 | 78.05 | 96.93 | .334 | .547 | 0.25 | -31.51 | [-99.62, 36.59] |
| Total | Total | Total | Low SES | PTSD | Child and adolescent psychotherapist | 44 | 24 | 95.16 | 140.06 | 73.50 | 79.46 | .419 | .637 | 0.18 | -21.66 | [-82.53, 39.22] |
| Total | Total | Total | Medium SES | PTSD | General practitioner | 225 | 156 | 92.38 | 127.44 | 75.09 | 104.52 | .148 | .363 | 0.15 | -17.29 | [-41.51, 6.93] |
| Total | Total | Total | Medium SES | PTSD | Pediatrician | 278 | 166 | 52.11 | 84.06 | 44.72 | 63.18 | .294 | .516 | 0.10 | -7.39 | [-22.17, 7.40] |
| Total | Total | Total | Medium SES | PTSD | Psychiatrist/Neurologist | 20 | 14 | 266.05 | 241.45 | 116.71 | 133.26 | .028 | .119 | 0.73 | -149.34 | [-289.02, -9.65] |
| Total | Total | Total | Medium SES | PTSD | Child and adolescent psychiatrist | 80 | 64 | 79.47 | 127.47 | 75.77 | 99.53 | .845 | .902 | 0.03 | -3.71 | [-41.81, 34.39] |
| Total | Total | Total | Medium SES | PTSD | Psychological psychotherapist | 15 | 9 | 122.93 | 100.46 | 135.78 | 178.75 | .847 | .902 | 0.10 | 12.84 | [-98.16, 123.84] |
| Total | Total | Total | Medium SES | PTSD | Child and adolescent psychotherapist | 111 | 68 | 71.71 | 112.30 | 53.24 | 85.51 | .216 | .450 | 0.18 | -18.48 | [-49.56, 12.61] |
| Total | Total | Total | High SES | PTSD | General practitioner | 75 | 37 | 112.84 | 145.90 | 76.08 | 121.73 | .164 | .382 | 0.27 | -36.76 | [-91.28, 17.76] |
| Total | Total | Total | High SES | PTSD | Pediatrician | 92 | 38 | 45.18 | 78.14 | 36.32 | 54.97 | .465 | .672 | 0.12 | -8.87 | [-36.16, 18.42] |
| Total | Total | Total | High SES | PTSD | Child and adolescent psychiatrist | 22 | 16 | 88.77 | 114.53 | 46.50 | 106.01 | .249 | .477 | 0.38 | -42.27 | [-113.80, 29.25] |
| Total | Total | Total | High SES | PTSD | Child and adolescent psychotherapist | 28 | 18 | 63.89 | 112.04 | 36.17 | 42.88 | .245 | .476 | 0.30 | -27.73 | [-82.04, 26.59] |
| Female | 10-13 | Total | Total | Anorexia nervosa | General practitioner | 56 | 55 | 76.07 | 106.00 | 69.73 | 93.58 | .739 | .831 | 0.06 | -6.34 | [-43.57, 30.88] |
| Female | 10-13 | Total | Total | Anorexia nervosa | Pediatrician | 75 | 95 | 59.68 | 93.80 | 45.98 | 65.23 | .284 | .511 | 0.17 | -13.70 | [-37.65, 10.25] |
| Female | 10-13 | Total | Total | Anorexia nervosa | Child and adolescent psychiatrist | 37 | 47 | 91.22 | 124.15 | 52.00 | 78.36 | .099 | .273 | 0.39 | -39.22 | [-82.75, 4.31] |
| Female | 10-13 | Total | Total | Anorexia nervosa | Child and adolescent psychotherapist | 55 | 72 | 83.18 | 102.04 | 59.12 | 76.26 | .147 | .362 | 0.27 | -24.06 | [-55.06, 6.94] |
| Female | 14-17 | Total | Total | Anorexia nervosa | General practitioner | 217 | 186 | 93.32 | 138.06 | 79.03 | 100.33 | .231 | .465 | 0.12 | -14.30 | [-38.21, 9.62] |
| Female | 14-17 | Total | Total | Anorexia nervosa | Pediatrician | 114 | 121 | 78.32 | 109.85 | 43.07 | 60.96 | .003 | **.029** | **0.40** | -35.25 | [-57.80, -12.70] |
| Female | 14-17 | Total | Total | Anorexia nervosa | Psychiatrist/Neurologist | 32 | 15 | 165.94 | 139.08 | 181.53 | 127.39 | .707 | .812 | 0.12 | 15.60 | [-67.54, 98.73] |
| Female | 14-17 | Total | Total | Anorexia nervosa | Child and adolescent psychiatrist | 110 | 106 | 90.65 | 125.87 | 63.55 | 88.40 | .068 | .211 | 0.25 | -27.10 | [-56.20, 2.01] |
| Female | 14-17 | Total | Total | Anorexia nervosa | Psychological psychotherapist | 24 | 18 | 141.71 | 156.47 | 151.83 | 134.11 | .823 | .893 | 0.07 | 10.12 | [-79.95, 100.20] |
| Female | 14-17 | Total | Total | Anorexia nervosa | Child and adolescent psychotherapist | 137 | 150 | 83.90 | 124.65 | 46.35 | 71.22 | .002 | **.024** | **0.37** | -37.54 | [-60.78, -14.31] |
| Male | 10-13 | Total | Total | Anorexia nervosa | Pediatrician | 11 | 10 | 29.09 | 48.22 | 44.90 | 64.25 | .536 | .714 | 0.28 | 15.81 | [-32.48, 64.10] |
| Male | 14-17 | Total | Total | Anorexia nervosa | General practitioner | 10 | 10 | 93.80 | 141.96 | 61.40 | 98.70 | .562 | .734 | 0.27 | -32.40 | [-139.56, 74.76] |
| Male | 14-17 | Total | Total | Anorexia nervosa | Pediatrician | 7 | 11 | 15.57 | 24.21 | 49.91 | 54.82 | .091 | .261 | 0.75 | 34.34 | [-9.07, 77.75] |
| Male | 14-17 | Total | Total | Anorexia nervosa | Child and adolescent psychotherapist | 6 | 8 | 66.33 | 90.98 | 7.25 | 7.30 | .173 | .395 | 1.00 | -59.08 | [-121.53, 3.36] |
| Female | Total | Urban | Total | Anorexia nervosa | General practitioner | 188 | 158 | 85.51 | 135.10 | 71.73 | 89.17 | .257 | .487 | 0.12 | -13.78 | [-38.40, 10.85] |
| Female | Total | Urban | Total | Anorexia nervosa | Pediatrician | 134 | 156 | 70.87 | 102.00 | 42.10 | 58.34 | .004 | **.037** | **0.35** | -28.77 | [-47.58, -9.96] |
| Female | Total | Urban | Total | Anorexia nervosa | Psychiatrist/Neurologist | 27 | 13 | 163.44 | 127.61 | 122.15 | 134.02 | .364 | .579 | 0.32 | -41.29 | [-127.09, 44.50] |
| Female | Total | Urban | Total | Anorexia nervosa | Child and adolescent psychiatrist | 108 | 113 | 95.28 | 128.11 | 54.95 | 80.49 | .006 | **.044** | **0.38** | -40.33 | [-68.41, -12.25] |
| Female | Total | Urban | Total | Anorexia nervosa | Psychological psychotherapist | 21 | 12 | 115.57 | 150.40 | 148.67 | 138.67 | .529 | .712 | 0.23 | 33.10 | [-70.70, 136.89] |
| Female | Total | Urban | Total | Anorexia nervosa | Child and adolescent psychotherapist | 128 | 157 | 85.31 | 131.08 | 53.44 | 77.02 | .016 | .086 | 0.30 | -31.87 | [-56.33, -7.41] |
| Male | Total | Urban | Total | Anorexia nervosa | General practitioner | 12 | 11 | 96.33 | 126.40 | 79.82 | 94.87 | .725 | .824 | 0.15 | -16.52 | [-108.55, 75.52] |
| Male | Total | Urban | Total | Anorexia nervosa | Pediatrician | 16 | 17 | 16.19 | 24.97 | 31.88 | 37.86 | .169 | .389 | 0.49 | 15.69 | [-6.34, 37.73] |
| Male | Total | Urban | Total | Anorexia nervosa | Child and adolescent psychiatrist | 9 | 9 | 103.67 | 102.27 | 88.78 | 139.08 | .800 | .876 | 0.12 | -14.89 | [-127.67, 97.90] |
| Male | Total | Urban | Total | Anorexia nervosa | Child and adolescent psychotherapist | 7 | 12 | 82.57 | 92.56 | 38.50 | 61.79 | .291 | .513 | 0.59 | -44.07 | [-113.17, 25.02] |
| Female | Total | Rural | Total | Anorexia nervosa | General practitioner | 88 | 84 | 100.68 | 125.67 | 85.75 | 114.35 | .416 | .634 | 0.12 | -14.93 | [-50.89, 21.03] |
| Female | Total | Rural | Total | Anorexia nervosa | Pediatrician | 59 | 66 | 70.00 | 108.22 | 46.44 | 70.76 | .158 | .375 | 0.26 | -23.56 | [-55.30, 8.18] |
| Female | Total | Rural | Total | Anorexia nervosa | Psychiatrist/Neurologist | 5 | 5 | 179.40 | 209.27 | 247.40 | 60.20 | .523 | .709 | 0.44 | 68.00 | [-122.87, 258.87] |
| Female | Total | Rural | Total | Anorexia nervosa | Child and adolescent psychiatrist | 40 | 41 | 77.58 | 115.29 | 72.71 | 96.76 | .838 | .898 | 0.05 | -4.87 | [-51.18, 41.44] |
| Female | Total | Rural | Total | Anorexia nervosa | Psychological psychotherapist | 7 | 11 | 182.43 | 148.92 | 104.55 | 117.63 | .269 | .496 | 0.60 | -77.88 | [-201.31, 45.55] |
| Female | Total | Rural | Total | Anorexia nervosa | Child and adolescent psychotherapist | 65 | 69 | 79.34 | 88.15 | 45.03 | 61.60 | .011 | .064 | 0.45 | -34.31 | [-59.94, -8.68] |
| Total | 10-13 | Total | Low SES | Anorexia nervosa | Pediatrician | 11 | 13 | 29.55 | 30.96 | 54.77 | 57.64 | .190 | .413 | 0.53 | 25.22 | [-12.85, 63.29] |
| Total | 10-13 | Total | Low SES | Anorexia nervosa | Child and adolescent psychotherapist | 7 | 8 | 144.86 | 165.23 | 68.38 | 68.35 | .291 | .513 | 0.62 | -76.48 | [-201.20, 48.23] |
| Total | 10-13 | Total | Medium SES | Anorexia nervosa | General practitioner | 35 | 41 | 69.54 | 93.81 | 60.95 | 80.15 | .672 | .788 | 0.10 | -8.59 | [-47.70, 30.51] |
| Total | 10-13 | Total | Medium SES | Anorexia nervosa | Pediatrician | 52 | 66 | 42.58 | 54.71 | 50.17 | 73.84 | .523 | .709 | 0.11 | 7.59 | [-16.44, 31.62] |
| Total | 10-13 | Total | Medium SES | Anorexia nervosa | Child and adolescent psychiatrist | 24 | 28 | 104.04 | 129.92 | 47.07 | 73.93 | .066 | .208 | 0.55 | -56.97 | [-113.41, -0.53] |
| Total | 10-13 | Total | Medium SES | Anorexia nervosa | Child and adolescent psychotherapist | 40 | 49 | 59.48 | 66.12 | 56.53 | 75.19 | .845 | .902 | 0.04 | -2.94 | [-32.71, 26.82] |
| Total | 10-13 | Total | High SES | Anorexia nervosa | General practitioner | 16 | 17 | 94.81 | 132.62 | 79.71 | 101.18 | .717 | .818 | 0.13 | -15.11 | [-95.29, 65.07] |
| Total | 10-13 | Total | High SES | Anorexia nervosa | Pediatrician | 23 | 26 | 98.13 | 145.09 | 30.54 | 37.19 | .040 | .146 | 0.66 | -67.59 | [-125.33, -9.86] |
| Total | 10-13 | Total | High SES | Anorexia nervosa | Child and adolescent psychiatrist | 14 | 15 | 85.43 | 125.89 | 56.07 | 86.59 | .475 | .681 | 0.27 | -29.36 | [-107.53, 48.81] |
| Total | 10-13 | Total | High SES | Anorexia nervosa | Child and adolescent psychotherapist | 11 | 22 | 136.82 | 125.45 | 62.59 | 81.61 | .096 | .270 | 0.76 | -74.23 | [-145.10, -3.35] |
| Total | 14-17 | Total | Low SES | Anorexia nervosa | General practitioner | 33 | 26 | 82.79 | 152.29 | 108.42 | 139.17 | .503 | .699 | 0.17 | 25.64 | [-49.75, 101.02] |
| Total | 14-17 | Total | Low SES | Anorexia nervosa | Pediatrician | 15 | 15 | 106.20 | 133.30 | 25.60 | 33.78 | .038 | .143 | 0.83 | -80.60 | [-150.19, -11.01] |
| Total | 14-17 | Total | Low SES | Anorexia nervosa | Child and adolescent psychiatrist | 16 | 8 | 117.50 | 148.99 | 67.25 | 143.95 | .439 | .653 | 0.34 | -50.25 | [-175.36, 74.86] |
| Total | 14-17 | Total | Low SES | Anorexia nervosa | Child and adolescent psychotherapist | 18 | 18 | 80.28 | 102.21 | 15.94 | 19.99 | .017 | .088 | 0.87 | -64.33 | [-112.45, -16.22] |
| Total | 14-17 | Total | Medium SES | Anorexia nervosa | General practitioner | 140 | 119 | 100.56 | 136.54 | 71.63 | 88.66 | .042 | .150 | 0.25 | -28.93 | [-57.52, -0.33] |
| Total | 14-17 | Total | Medium SES | Anorexia nervosa | Pediatrician | 76 | 84 | 82.95 | 117.10 | 33.24 | 43.52 | .001 | **.012** | **0.57** | -49.71 | [-76.59, -22.83] |
| Total | 14-17 | Total | Medium SES | Anorexia nervosa | Psychiatrist/Neurologist | 21 | 11 | 200.67 | 153.63 | 172.82 | 117.39 | .573 | .739 | 0.20 | -27.85 | [-131.86, 76.16] |
| Total | 14-17 | Total | Medium SES | Anorexia nervosa | Child and adolescent psychiatrist | 68 | 77 | 98.24 | 129.73 | 76.31 | 96.56 | .256 | .485 | 0.19 | -21.92 | [-58.88, 15.04] |
| Total | 14-17 | Total | Medium SES | Anorexia nervosa | Psychological psychotherapist | 17 | 16 | 106.47 | 97.78 | 151.50 | 134.44 | .283 | .511 | 0.38 | 45.03 | [-34.82, 124.88] |
| Total | 14-17 | Total | Medium SES | Anorexia nervosa | Child and adolescent psychotherapist | 91 | 100 | 77.35 | 114.18 | 48.34 | 67.48 | .036 | .142 | 0.31 | -29.01 | [-55.33, -2.69] |
| Total | 14-17 | Total | High SES | Anorexia nervosa | General practitioner | 54 | 51 | 81.09 | 133.59 | 77.84 | 101.21 | .888 | .928 | 0.03 | -3.25 | [-48.78, 42.28] |
| Total | 14-17 | Total | High SES | Anorexia nervosa | Pediatrician | 30 | 33 | 38.00 | 43.77 | 78.30 | 88.34 | .024 | .109 | 0.57 | 40.30 | [5.32, 75.28] |
| Total | 14-17 | Total | High SES | Anorexia nervosa | Psychiatrist/Neurologist | 7 | 5 | 105.14 | 80.18 | 158.60 | 167.08 | .537 | .714 | 0.44 | 53.46 | [-87.22, 194.13] |
| Total | 14-17 | Total | High SES | Anorexia nervosa | Child and adolescent psychiatrist | 30 | 30 | 62.13 | 92.69 | 41.37 | 63.18 | .315 | .533 | 0.26 | -20.77 | [-60.91, 19.38] |
| Total | 14-17 | Total | High SES | Anorexia nervosa | Child and adolescent psychotherapist | 34 | 40 | 100.24 | 154.93 | 47.25 | 86.80 | .083 | .243 | 0.43 | -52.99 | [-109.13, 3.16] |
| Female | 0-9 | Total | Total | Anxiety disorder | General practitioner | 304 | 141 | 113.79 | 141.13 | 90.18 | 95.79 | .039 | .146 | 0.18 | -23.62 | [-49.29, 2.06] |
| Female | 0-9 | Total | Total | Anxiety disorder | Pediatrician | 853 | 481 | 41.86 | 69.34 | 31.88 | 52.47 | .003 | .030 | 0.16 | -9.98 | [-17.10, -2.85] |
| Female | 0-9 | Total | Total | Anxiety disorder | Child and adolescent psychiatrist | 171 | 95 | 74.12 | 105.27 | 60.09 | 85.46 | .240 | .473 | 0.14 | -14.02 | [-38.77, 10.73] |
| Female | 0-9 | Total | Total | Anxiety disorder | Child and adolescent psychotherapist | 149 | 85 | 98.48 | 151.85 | 63.47 | 92.61 | .030 | .123 | 0.26 | -35.01 | [-70.56, 0.55] |
| Female | 10-13 | Total | Total | Anxiety disorder | General practitioner | 420 | 256 | 107.60 | 137.63 | 87.28 | 102.43 | .029 | .122 | 0.16 | -20.32 | [-39.82, -0.82] |
| Female | 10-13 | Total | Total | Anxiety disorder | Pediatrician | 587 | 432 | 51.19 | 83.64 | 44.20 | 67.56 | .141 | .351 | 0.09 | -6.98 | [-16.58, 2.61] |
| Female | 10-13 | Total | Total | Anxiety disorder | Psychiatrist/Neurologist | 21 | 10 | 149.00 | 149.51 | 107.70 | 99.69 | .371 | .587 | 0.30 | -41.30 | [-143.73, 61.13] |
| Female | 10-13 | Total | Total | Anxiety disorder | Child and adolescent psychiatrist | 261 | 194 | 88.83 | 130.87 | 57.90 | 78.26 | .002 | **.023** | **0.28** | -30.93 | [-51.65, -10.21] |
| Female | 10-13 | Total | Total | Anxiety disorder | Psychological psychotherapist | 17 | 13 | 36.00 | 52.09 | 65.69 | 68.21 | .206 | .435 | 0.50 | 29.69 | [-13.30, 72.69] |
| Female | 10-13 | Total | Total | Anxiety disorder | Child and adolescent psychotherapist | 281 | 230 | 92.08 | 135.89 | 59.95 | 96.82 | .002 | **.023** | **0.27** | -32.13 | [-53.03, -11.23] |
| Female | 14-17 | Total | Total | Anxiety disorder | General practitioner | 618 | 451 | 79.15 | 115.24 | 83.21 | 110.44 | .561 | .734 | 0.04 | 4.05 | [-9.69, 17.80] |
| Female | 14-17 | Total | Total | Anxiety disorder | Pediatrician | 373 | 340 | 76.72 | 117.22 | 57.71 | 88.29 | .014 | .080 | 0.18 | -19.01 | [-34.35, -3.66] |
| Female | 14-17 | Total | Total | Anxiety disorder | Psychiatrist/Neurologist | 104 | 32 | 219.61 | 193.74 | 151.78 | 140.32 | .033 | .133 | 0.37 | -67.82 | [-140.24, 4.59] |
| Female | 14-17 | Total | Total | Anxiety disorder | Child and adolescent psychiatrist | 221 | 221 | 82.78 | 124.95 | 65.87 | 96.25 | .112 | .298 | 0.15 | -16.90 | [-37.70, 3.89] |
| Female | 14-17 | Total | Total | Anxiety disorder | Psychological psychotherapist | 57 | 24 | 146.82 | 169.63 | 118.50 | 139.45 | .438 | .653 | 0.18 | -28.32 | [-105.31, 48.66] |
| Female | 14-17 | Total | Total | Anxiety disorder | Child and adolescent psychotherapist | 284 | 243 | 86.31 | 140.10 | 63.00 | 97.62 | .026 | .113 | 0.19 | -23.31 | [-44.27, -2.35] |
| Male | 0-9 | Total | Total | Anxiety disorder | General practitioner | 444 | 233 | 126.72 | 150.52 | 111.49 | 119.46 | .151 | .366 | 0.11 | -15.23 | [-37.53, 7.06] |
| Male | 0-9 | Total | Total | Anxiety disorder | Pediatrician | 1,303 | 759 | 38.73 | 67.36 | 36.16 | 57.30 | .359 | .574 | 0.04 | -2.56 | [-8.28, 3.15] |
| Male | 0-9 | Total | Total | Anxiety disorder | Child and adolescent psychiatrist | 362 | 205 | 88.21 | 131.25 | 70.10 | 107.60 | .077 | .227 | 0.15 | -18.10 | [-39.22, 3.01] |
| Male | 0-9 | Total | Total | Anxiety disorder | Psychological psychotherapist | 13 | 5 | 141.15 | 204.15 | 134.40 | 156.46 | .942 | .962 | 0.03 | -6.75 | [-206.16, 192.66] |
| Male | 0-9 | Total | Total | Anxiety disorder | Child and adolescent psychotherapist | 232 | 135 | 98.17 | 141.97 | 76.30 | 106.26 | .095 | .269 | 0.17 | -21.87 | [-49.45, 5.71] |
| Male | 10-13 | Total | Total | Anxiety disorder | General practitioner | 390 | 198 | 111.94 | 132.45 | 112.96 | 130.10 | .929 | .954 | 0.01 | 1.02 | [-21.50, 23.54] |
| Male | 10-13 | Total | Total | Anxiety disorder | Pediatrician | 540 | 386 | 52.06 | 83.96 | 50.02 | 67.29 | .681 | .792 | 0.03 | -2.05 | [-12.16, 8.07] |
| Male | 10-13 | Total | Total | Anxiety disorder | Psychiatrist/Neurologist | 11 | 7 | 113.00 | 96.04 | 80.71 | 101.11 | .514 | .703 | 0.33 | -32.29 | [-125.13, 60.56] |
| Male | 10-13 | Total | Total | Anxiety disorder | Child and adolescent psychiatrist | 273 | 161 | 82.65 | 119.40 | 60.50 | 88.41 | .028 | .119 | 0.20 | -22.15 | [-43.37, -0.92] |
| Male | 10-13 | Total | Total | Anxiety disorder | Psychological psychotherapist | 19 | 9 | 143.68 | 181.32 | 94.00 | 164.35 | .480 | .685 | 0.28 | -49.68 | [-189.49, 90.12] |
| Male | 10-13 | Total | Total | Anxiety disorder | Child and adolescent psychotherapist | 198 | 130 | 99.93 | 143.57 | 77.78 | 105.69 | .109 | .293 | 0.17 | -22.16 | [-50.90, 6.59] |
| Male | 14-17 | Total | Total | Anxiety disorder | General practitioner | 291 | 235 | 81.92 | 111.45 | 92.60 | 121.52 | .299 | .520 | 0.09 | 10.68 | [-9.27, 30.63] |
| Male | 14-17 | Total | Total | Anxiety disorder | Pediatrician | 202 | 197 | 54.08 | 90.57 | 53.66 | 80.94 | .961 | .973 | 0.00 | -0.42 | [-17.29, 16.45] |
| Male | 14-17 | Total | Total | Anxiety disorder | Psychiatrist/Neurologist | 34 | 30 | 182.65 | 170.47 | 160.80 | 162.83 | .602 | .755 | 0.13 | -21.85 | [-103.81, 60.11] |
| Male | 14-17 | Total | Total | Anxiety disorder | Child and adolescent psychiatrist | 107 | 99 | 94.45 | 120.76 | 64.35 | 95.02 | .047 | .165 | 0.28 | -30.10 | [-59.93, -0.26] |
| Male | 14-17 | Total | Total | Anxiety disorder | Psychological psychotherapist | 20 | 9 | 93.90 | 96.18 | 102.44 | 146.24 | .876 | .921 | 0.08 | 8.54 | [-80.63, 97.72] |
| Male | 14-17 | Total | Total | Anxiety disorder | Child and adolescent psychotherapist | 102 | 88 | 95.35 | 128.47 | 70.72 | 88.93 | .122 | .312 | 0.22 | -24.64 | [-56.55, 7.28] |
| Female | Total | Urban | Total | Anxiety disorder | General practitioner | 875 | 551 | 101.96 | 134.75 | 92.99 | 110.34 | .171 | .392 | 0.07 | -8.97 | [-22.39, 4.45] |
| Female | Total | Urban | Total | Anxiety disorder | Pediatrician | 1,325 | 904 | 49.86 | 83.30 | 43.86 | 70.81 | .068 | .211 | 0.08 | -6.00 | [-12.64, 0.63] |
| Female | Total | Urban | Total | Anxiety disorder | Psychiatrist/Neurologist | 87 | 28 | 219.69 | 186.68 | 136.25 | 138.33 | .014 | .079 | 0.47 | -83.44 | [-158.54, -8.34] |
| Female | Total | Urban | Total | Anxiety disorder | Child and adolescent psychiatrist | 483 | 396 | 86.23 | 126.51 | 61.68 | 86.09 | .001 | **.012** | **0.22** | -24.55 | [-39.19, -9.92] |
| Female | Total | Urban | Total | Anxiety disorder | Psychological psychotherapist | 61 | 26 | 128.07 | 168.83 | 106.42 | 123.70 | .508 | .701 | 0.14 | -21.64 | [-93.67, 50.39] |
| Female | Total | Urban | Total | Anxiety disorder | Child and adolescent psychotherapist | 532 | 412 | 94.69 | 145.68 | 61.44 | 97.23 | < .001 | **.002** | **0.26** | -33.24 | [-49.56, -16.93] |
| Male | Total | Urban | Total | Anxiety disorder | General practitioner | 696 | 414 | 115.79 | 140.40 | 114.27 | 125.35 | .852 | .904 | 0.01 | -1.52 | [-17.94, 14.90] |
| Male | Total | Urban | Total | Anxiety disorder | Pediatrician | 1,459 | 941 | 40.17 | 67.89 | 43.29 | 63.96 | .254 | .484 | 0.05 | 3.13 | [-2.31, 8.56] |
| Male | Total | Urban | Total | Anxiety disorder | Psychiatrist/Neurologist | 40 | 31 | 143.05 | 152.27 | 150.35 | 155.14 | .843 | .902 | 0.05 | 7.30 | [-64.70, 79.31] |
| Male | Total | Urban | Total | Anxiety disorder | Child and adolescent psychiatrist | 546 | 339 | 85.71 | 120.80 | 63.45 | 96.38 | .003 | .026 | 0.20 | -22.27 | [-37.46, -7.07] |
| Male | Total | Urban | Total | Anxiety disorder | Psychological psychotherapist | 37 | 16 | 122.05 | 145.06 | 139.69 | 168.85 | .719 | .818 | 0.12 | 17.63 | [-71.77, 107.04] |
| Male | Total | Urban | Total | Anxiety disorder | Child and adolescent psychotherapist | 356 | 243 | 93.45 | 138.46 | 72.25 | 95.07 | .027 | .116 | 0.17 | -21.21 | [-41.22, -1.19] |
| Female | Total | Rural | Total | Anxiety disorder | General practitioner | 467 | 296 | 84.56 | 118.39 | 72.02 | 95.15 | .108 | .292 | 0.11 | -12.54 | [-28.55, 3.48] |
| Female | Total | Rural | Total | Anxiety disorder | Pediatrician | 488 | 348 | 57.99 | 95.18 | 40.86 | 66.05 | .002 | **.024** | **0.20** | -17.13 | [-28.72, -5.54] |
| Female | Total | Rural | Total | Anxiety disorder | Psychiatrist/Neurologist | 48 | 15 | 188.00 | 192.85 | 142.73 | 122.88 | .290 | .513 | 0.25 | -45.27 | [-149.18, 58.64] |
| Female | Total | Rural | Total | Anxiety disorder | Child and adolescent psychiatrist | 170 | 114 | 73.54 | 110.40 | 62.05 | 93.41 | .346 | .557 | 0.11 | -11.49 | [-36.15, 13.17] |
| Female | Total | Rural | Total | Anxiety disorder | Psychological psychotherapist | 24 | 14 | 89.50 | 101.31 | 73.14 | 106.50 | .646 | .777 | 0.16 | -16.36 | [-84.39, 51.68] |
| Female | Total | Rural | Total | Anxiety disorder | Child and adolescent psychotherapist | 182 | 145 | 80.69 | 125.64 | 63.26 | 94.50 | .153 | .367 | 0.15 | -17.42 | [-42.06, 7.21] |
| Male | Total | Rural | Total | Anxiety disorder | General practitioner | 429 | 252 | 100.63 | 128.41 | 90.47 | 119.45 | .298 | .520 | 0.08 | -10.16 | [-29.63, 9.31] |
| Male | Total | Rural | Total | Anxiety disorder | Pediatrician | 586 | 401 | 52.73 | 89.28 | 41.37 | 66.06 | .022 | .101 | 0.14 | -11.36 | [-21.60, -1.11] |
| Male | Total | Rural | Total | Anxiety disorder | Psychiatrist/Neurologist | 27 | 10 | 189.19 | 196.12 | 124.80 | 140.00 | .280 | .510 | 0.35 | -64.39 | [-197.41, 68.64] |
| Male | Total | Rural | Total | Anxiety disorder | Child and adolescent psychiatrist | 196 | 126 | 90.83 | 137.70 | 71.23 | 104.33 | .149 | .364 | 0.16 | -19.60 | [-47.73, 8.54] |
| Male | Total | Rural | Total | Anxiety disorder | Psychological psychotherapist | 15 | 7 | 128.47 | 196.21 | 29.29 | 24.34 | .075 | .225 | 0.60 | -99.18 | [-246.95, 48.58] |
| Male | Total | Rural | Total | Anxiety disorder | Child and adolescent psychotherapist | 176 | 110 | 108.06 | 142.50 | 82.53 | 115.32 | .098 | .272 | 0.19 | -25.54 | [-57.15, 6.08] |
| Total | 0-9 | Total | Low SES | Anxiety disorder | General practitioner | 116 | 58 | 134.54 | 137.15 | 111.14 | 111.00 | .229 | .461 | 0.18 | -23.41 | [-64.09, 17.28] |
| Total | 0-9 | Total | Low SES | Anxiety disorder | Pediatrician | 335 | 187 | 35.48 | 56.11 | 31.14 | 45.06 | .335 | .547 | 0.08 | -4.34 | [-13.72, 5.04] |
| Total | 0-9 | Total | Low SES | Anxiety disorder | Child and adolescent psychiatrist | 79 | 46 | 79.32 | 121.20 | 68.39 | 100.10 | .588 | .748 | 0.10 | -10.93 | [-52.34, 30.49] |
| Total | 0-9 | Total | Low SES | Anxiety disorder | Child and adolescent psychotherapist | 63 | 42 | 70.52 | 91.53 | 80.71 | 104.09 | .608 | .759 | 0.11 | 10.19 | [-27.58, 47.96] |
| Total | 0-9 | Total | Medium SES | Anxiety disorder | General practitioner | 476 | 224 | 120.76 | 149.38 | 99.06 | 114.62 | .035 | .137 | 0.16 | -21.70 | [-43.81, 0.41] |
| Total | 0-9 | Total | Medium SES | Anxiety disorder | Pediatrician | 1,357 | 751 | 40.36 | 69.68 | 35.02 | 56.75 | .057 | .187 | 0.08 | -5.34 | [-11.16, 0.49] |
| Total | 0-9 | Total | Medium SES | Anxiety disorder | Child and adolescent psychiatrist | 359 | 201 | 83.11 | 122.94 | 65.19 | 101.91 | .065 | .207 | 0.15 | -17.92 | [-37.92, 2.08] |
| Total | 0-9 | Total | Medium SES | Anxiety disorder | Psychological psychotherapist | 17 | 5 | 132.65 | 192.85 | 104.80 | 161.14 | .755 | .843 | 0.15 | -27.85 | [-214.25, 158.56] |
| Total | 0-9 | Total | Medium SES | Anxiety disorder | Child and adolescent psychotherapist | 238 | 127 | 111.70 | 158.15 | 74.10 | 103.04 | .007 | **.047** | **0.27** | -37.60 | [-68.07, -7.12] |
| Total | 0-9 | Total | High SES | Anxiety disorder | General practitioner | 156 | 92 | 113.90 | 146.15 | 109.30 | 104.45 | .774 | .857 | 0.03 | -4.60 | [-38.68, 29.48] |
| Total | 0-9 | Total | High SES | Anxiety disorder | Pediatrician | 464 | 302 | 42.06 | 71.48 | 35.29 | 58.22 | .152 | .366 | 0.10 | -6.76 | [-16.41, 2.88] |
| Total | 0-9 | Total | High SES | Anxiety disorder | Child and adolescent psychiatrist | 95 | 53 | 89.48 | 129.01 | 72.26 | 100.46 | .369 | .586 | 0.14 | -17.22 | [-57.42, 22.98] |
| Total | 0-9 | Total | High SES | Anxiety disorder | Child and adolescent psychotherapist | 80 | 51 | 80.26 | 137.96 | 56.75 | 94.30 | .249 | .477 | 0.19 | -23.52 | [-66.68, 19.64] |
| Total | 10-13 | Total | Low SES | Anxiety disorder | General practitioner | 123 | 60 | 111.59 | 131.55 | 112.65 | 118.91 | .956 | .971 | 0.01 | 1.06 | [-38.31, 40.44] |
| Total | 10-13 | Total | Low SES | Anxiety disorder | Pediatrician | 190 | 143 | 57.47 | 89.37 | 42.29 | 64.78 | .073 | .222 | 0.19 | -15.19 | [-32.49, 2.12] |
| Total | 10-13 | Total | Low SES | Anxiety disorder | Child and adolescent psychiatrist | 75 | 62 | 89.39 | 134.76 | 46.60 | 71.99 | .019 | .095 | 0.39 | -42.79 | [-80.09, -5.48] |
| Total | 10-13 | Total | Low SES | Anxiety disorder | Child and adolescent psychotherapist | 75 | 65 | 94.03 | 120.38 | 58.11 | 83.91 | .041 | .147 | 0.34 | -35.92 | [-70.81, -1.03] |
| Total | 10-13 | Total | Medium SES | Anxiety disorder | General practitioner | 528 | 312 | 112.03 | 137.84 | 92.93 | 112.40 | .029 | .123 | 0.15 | -19.11 | [-37.16, -1.05] |
| Total | 10-13 | Total | Medium SES | Anxiety disorder | Pediatrician | 710 | 535 | 49.29 | 80.23 | 47.00 | 65.91 | .581 | .746 | 0.03 | -2.29 | [-10.64, 6.06] |
| Total | 10-13 | Total | Medium SES | Anxiety disorder | Psychiatrist/Neurologist | 23 | 10 | 147.70 | 149.65 | 68.70 | 88.58 | .070 | .216 | 0.59 | -79.00 | [-179.07, 21.08] |
| Total | 10-13 | Total | Medium SES | Anxiety disorder | Child and adolescent psychiatrist | 329 | 232 | 83.81 | 120.01 | 62.48 | 87.87 | .015 | .084 | 0.20 | -21.33 | [-39.46, -3.20] |
| Total | 10-13 | Total | Medium SES | Anxiety disorder | Psychological psychotherapist | 26 | 15 | 83.92 | 111.05 | 90.93 | 134.70 | .866 | .915 | 0.06 | 7.01 | [-69.30, 83.32] |
| Total | 10-13 | Total | Medium SES | Anxiety disorder | Child and adolescent psychotherapist | 307 | 229 | 101.24 | 146.26 | 63.50 | 102.62 | < .001 | **.009** | **0.29** | -37.74 | [-59.89, -15.59] |
| Total | 10-13 | Total | High SES | Anxiety disorder | General practitioner | 159 | 82 | 100.43 | 128.86 | 109.24 | 126.15 | .611 | .761 | 0.07 | 8.82 | [-25.28, 42.91] |
| Total | 10-13 | Total | High SES | Anxiety disorder | Pediatrician | 227 | 140 | 53.93 | 89.61 | 51.49 | 75.66 | .780 | .862 | 0.03 | -2.44 | [-20.25, 15.38] |
| Total | 10-13 | Total | High SES | Anxiety disorder | Psychiatrist/Neurologist | 8 | 5 | 111.75 | 80.31 | 175.40 | 94.06 | .250 | .477 | 0.74 | 63.65 | [-31.96, 159.26] |
| Total | 10-13 | Total | High SES | Anxiety disorder | Child and adolescent psychiatrist | 130 | 61 | 88.23 | 132.52 | 58.85 | 73.17 | .051 | .173 | 0.25 | -29.38 | [-64.96, 6.21] |
| Total | 10-13 | Total | High SES | Anxiety disorder | Child and adolescent psychotherapist | 97 | 66 | 77.61 | 128.26 | 84.56 | 106.28 | .707 | .812 | 0.06 | 6.95 | [-30.54, 44.44] |
| Total | 14-17 | Total | Low SES | Anxiety disorder | General practitioner | 146 | 108 | 62.46 | 79.77 | 97.19 | 132.94 | .017 | .087 | 0.33 | 34.74 | [8.45, 61.02] |
| Total | 14-17 | Total | Low SES | Anxiety disorder | Pediatrician | 110 | 98 | 74.04 | 105.50 | 59.83 | 96.31 | .311 | .527 | 0.14 | -14.21 | [-41.78, 13.36] |
| Total | 14-17 | Total | Low SES | Anxiety disorder | Psychiatrist/Neurologist | 18 | 6 | 250.94 | 210.52 | 148.33 | 166.06 | .250 | .477 | 0.51 | -102.61 | [-288.58, 83.36] |
| Total | 14-17 | Total | Low SES | Anxiety disorder | Child and adolescent psychiatrist | 53 | 42 | 91.49 | 123.62 | 70.40 | 115.77 | .395 | .610 | 0.18 | -21.09 | [-69.77, 27.59] |
| Total | 14-17 | Total | Low SES | Anxiety disorder | Child and adolescent psychotherapist | 51 | 52 | 70.94 | 88.42 | 65.87 | 109.05 | .796 | .873 | 0.05 | -5.08 | [-43.46, 33.31] |
| Total | 14-17 | Total | Medium SES | Anxiety disorder | General practitioner | 571 | 455 | 82.94 | 115.51 | 83.32 | 107.34 | .957 | .971 | 0.00 | 0.38 | [-13.41, 14.17] |
| Total | 14-17 | Total | Medium SES | Anxiety disorder | Pediatrician | 358 | 341 | 71.36 | 117.83 | 55.68 | 77.65 | .037 | .143 | 0.16 | -15.69 | [-30.56, -0.82] |
| Total | 14-17 | Total | Medium SES | Anxiety disorder | Psychiatrist/Neurologist | 90 | 44 | 192.22 | 177.07 | 152.43 | 142.44 | .165 | .384 | 0.24 | -39.79 | [-99.85, 20.27] |
| Total | 14-17 | Total | Medium SES | Anxiety disorder | Child and adolescent psychiatrist | 215 | 215 | 82.12 | 121.24 | 64.68 | 90.25 | .091 | .261 | 0.16 | -17.44 | [-37.65, 2.76] |
| Total | 14-17 | Total | Medium SES | Anxiety disorder | Psychological psychotherapist | 47 | 21 | 136.15 | 160.73 | 112.29 | 157.14 | .569 | .739 | 0.15 | -23.86 | [-106.00, 58.27] |
| Total | 14-17 | Total | Medium SES | Anxiety disorder | Child and adolescent psychotherapist | 238 | 222 | 96.94 | 144.02 | 67.18 | 96.65 | .009 | .058 | 0.24 | -29.76 | [-52.33, -7.18] |
| Total | 14-17 | Total | High SES | Anxiety disorder | General practitioner | 192 | 122 | 84.79 | 129.75 | 88.93 | 122.48 | .775 | .857 | 0.03 | 4.15 | [-24.67, 32.96] |
| Total | 14-17 | Total | High SES | Anxiety disorder | Pediatrician | 107 | 97 | 54.66 | 77.14 | 53.18 | 99.97 | .906 | .940 | 0.02 | -1.49 | [-25.87, 22.89] |
| Total | 14-17 | Total | High SES | Anxiety disorder | Psychiatrist/Neurologist | 30 | 12 | 241.07 | 205.84 | 173.67 | 182.30 | .309 | .527 | 0.34 | -67.40 | [-201.06, 66.26] |
| Total | 14-17 | Total | High SES | Anxiety disorder | Child and adolescent psychiatrist | 60 | 63 | 98.25 | 132.51 | 64.54 | 100.73 | .116 | .306 | 0.29 | -33.71 | [-75.18, 7.76] |
| Total | 14-17 | Total | High SES | Anxiety disorder | Psychological psychotherapist | 16 | 9 | 137.38 | 183.34 | 108.67 | 122.96 | .645 | .777 | 0.17 | -28.71 | [-163.35, 105.93] |
| Total | 14-17 | Total | High SES | Anxiety disorder | Child and adolescent psychotherapist | 97 | 56 | 77.80 | 139.84 | 56.91 | 76.09 | .233 | .467 | 0.17 | -20.89 | [-60.56, 18.77] |
| Female | 0-9 | Total | Total | Depressive disorder | General practitioner | 19 | 6 | 110.21 | 136.76 | 40.83 | 32.03 | .053 | .178 | 0.57 | -69.38 | [-181.26, 42.51] |
| Female | 0-9 | Total | Total | Depressive disorder | Pediatrician | 34 | 20 | 49.09 | 65.94 | 51.40 | 61.83 | .898 | .935 | 0.04 | 2.31 | [-33.30, 37.92] |
| Female | 0-9 | Total | Total | Depressive disorder | Child and adolescent psychotherapist | 5 | 5 | 134.00 | 249.24 | 58.60 | 63.79 | .548 | .725 | 0.41 | -75.40 | [-300.91, 150.11] |
| Female | 10-13 | Total | Total | Depressive disorder | General practitioner | 359 | 268 | 102.19 | 134.95 | 79.56 | 101.22 | .017 | .087 | 0.19 | -22.63 | [-41.89, -3.38] |
| Female | 10-13 | Total | Total | Depressive disorder | Pediatrician | 360 | 335 | 73.83 | 103.11 | 56.07 | 75.78 | .010 | .060 | 0.20 | -17.77 | [-31.30, -4.23] |
| Female | 10-13 | Total | Total | Depressive disorder | Psychiatrist/Neurologist | 12 | 11 | 239.17 | 184.40 | 131.45 | 149.16 | .138 | .346 | 0.64 | -107.71 | [-245.60, 30.18] |
| Female | 10-13 | Total | Total | Depressive disorder | Child and adolescent psychiatrist | 244 | 208 | 90.54 | 135.64 | 61.31 | 88.24 | .006 | **.046** | **0.25** | -29.22 | [-50.73, -7.72] |
| Female | 10-13 | Total | Total | Depressive disorder | Psychological psychotherapist | 22 | 11 | 95.73 | 106.31 | 69.00 | 92.50 | .465 | .672 | 0.26 | -26.73 | [-100.60, 47.14] |
| Female | 10-13 | Total | Total | Depressive disorder | Child and adolescent psychotherapist | 269 | 242 | 84.35 | 128.59 | 61.30 | 85.33 | .016 | .086 | 0.21 | -23.05 | [-42.19, -3.90] |
| Female | 14-17 | Total | Total | Depressive disorder | General practitioner | 1,333 | 966 | 76.58 | 117.62 | 72.45 | 99.04 | .361 | .576 | 0.04 | -4.14 | [-13.26, 4.99] |
| Female | 14-17 | Total | Total | Depressive disorder | Pediatrician | 570 | 546 | 80.63 | 113.41 | 62.94 | 86.26 | .003 | .032 | 0.18 | -17.69 | [-29.55, -5.83] |
| Female | 14-17 | Total | Total | Depressive disorder | Psychiatrist/Neurologist | 239 | 145 | 194.95 | 185.48 | 143.79 | 136.09 | .002 | **.024** | **0.30** | -51.16 | [-85.94, -16.38] |
| Female | 14-17 | Total | Total | Depressive disorder | Child and adolescent psychiatrist | 570 | 547 | 81.39 | 121.15 | 65.59 | 97.94 | .016 | .086 | 0.14 | -15.81 | [-28.76, -2.86] |
| Female | 14-17 | Total | Total | Depressive disorder | Psychological psychotherapist | 128 | 81 | 153.34 | 170.35 | 102.01 | 134.10 | .016 | .086 | 0.33 | -51.33 | [-95.11, -7.55] |
| Female | 14-17 | Total | Total | Depressive disorder | Child and adolescent psychotherapist | 649 | 605 | 75.14 | 120.95 | 58.51 | 87.79 | .005 | .041 | 0.16 | -16.63 | [-28.40, -4.86] |
| Male | 0-9 | Total | Total | Depressive disorder | General practitioner | 39 | 11 | 91.95 | 144.54 | 50.18 | 42.47 | .121 | .312 | 0.32 | -41.77 | [-128.79, 45.26] |
| Male | 0-9 | Total | Total | Depressive disorder | Pediatrician | 68 | 35 | 78.24 | 106.13 | 64.17 | 83.46 | .464 | .672 | 0.14 | -14.06 | [-54.46, 26.34] |
| Male | 0-9 | Total | Total | Depressive disorder | Child and adolescent psychiatrist | 30 | 12 | 68.00 | 75.36 | 84.42 | 123.23 | .673 | .788 | 0.18 | 16.42 | [-44.55, 77.38] |
| Male | 0-9 | Total | Total | Depressive disorder | Child and adolescent psychotherapist | 18 | 8 | 134.50 | 175.70 | 28.25 | 35.29 | .024 | .107 | 0.71 | -106.25 | [-230.43, 17.93] |
| Male | 10-13 | Total | Total | Depressive disorder | General practitioner | 159 | 68 | 79.40 | 98.92 | 102.16 | 125.96 | .188 | .410 | 0.21 | 22.76 | [-7.82, 53.34] |
| Male | 10-13 | Total | Total | Depressive disorder | Pediatrician | 163 | 96 | 68.55 | 99.45 | 57.21 | 63.80 | .265 | .496 | 0.13 | -11.34 | [-33.52, 10.85] |
| Male | 10-13 | Total | Total | Depressive disorder | Child and adolescent psychiatrist | 89 | 50 | 112.42 | 162.96 | 51.80 | 77.08 | .004 | **.033** | **0.44** | -60.62 | [-108.59, -12.64] |
| Male | 10-13 | Total | Total | Depressive disorder | Child and adolescent psychotherapist | 76 | 59 | 91.00 | 149.67 | 54.34 | 66.00 | .059 | .191 | 0.30 | -36.66 | [-77.66, 4.34] |
| Male | 14-17 | Total | Total | Depressive disorder | General practitioner | 525 | 309 | 85.61 | 124.11 | 67.74 | 97.24 | .021 | .099 | 0.16 | -17.87 | [-34.02, -1.72] |
| Male | 14-17 | Total | Total | Depressive disorder | Pediatrician | 212 | 150 | 68.60 | 110.82 | 60.83 | 82.29 | .445 | .654 | 0.08 | -7.77 | [-28.68, 13.14] |
| Male | 14-17 | Total | Total | Depressive disorder | Psychiatrist/Neurologist | 99 | 51 | 185.57 | 195.26 | 155.69 | 144.24 | .291 | .513 | 0.17 | -29.88 | [-90.57, 30.81] |
| Male | 14-17 | Total | Total | Depressive disorder | Child and adolescent psychiatrist | 188 | 136 | 83.06 | 126.47 | 46.77 | 72.21 | .001 | **.017** | **0.34** | -36.29 | [-59.93, -12.66] |
| Male | 14-17 | Total | Total | Depressive disorder | Psychological psychotherapist | 47 | 32 | 182.04 | 192.06 | 80.88 | 110.25 | .004 | **.036** | **0.62** | -101.17 | [-174.88, -27.45] |
| Male | 14-17 | Total | Total | Depressive disorder | Child and adolescent psychotherapist | 186 | 123 | 86.42 | 129.24 | 48.15 | 90.10 | .002 | **.025** | **0.33** | -38.27 | [-64.53, -12.01] |
| Female | Total | Urban | Total | Depressive disorder | General practitioner | 1,171 | 830 | 87.45 | 127.53 | 74.66 | 98.86 | .012 | .068 | 0.11 | -12.79 | [-23.15, -2.43] |
| Female | Total | Urban | Total | Depressive disorder | Pediatrician | 704 | 654 | 75.48 | 106.35 | 58.83 | 80.27 | .001 | .016 | 0.18 | -16.65 | [-26.73, -6.57] |
| Female | Total | Urban | Total | Depressive disorder | Psychiatrist/Neurologist | 184 | 105 | 201.50 | 191.98 | 139.48 | 136.12 | .002 | **.020** | **0.36** | -62.02 | [-103.69, -20.36] |
| Female | Total | Urban | Total | Depressive disorder | Child and adolescent psychiatrist | 601 | 541 | 83.32 | 125.29 | 63.25 | 94.95 | .002 | .024 | 0.18 | -20.07 | [-33.08, -7.07] |
| Female | Total | Urban | Total | Depressive disorder | Psychological psychotherapist | 105 | 65 | 141.19 | 167.91 | 86.15 | 109.36 | .011 | .064 | 0.37 | -55.04 | [-100.93, -9.15] |
| Female | Total | Urban | Total | Depressive disorder | Child and adolescent psychotherapist | 653 | 605 | 73.70 | 117.46 | 58.08 | 86.93 | .007 | .050 | 0.15 | -15.62 | [-27.12, -4.13] |
| Male | Total | Urban | Total | Depressive disorder | General practitioner | 506 | 266 | 93.20 | 124.64 | 79.36 | 107.92 | .109 | .294 | 0.12 | -13.83 | [-31.52, 3.85] |
| Male | Total | Urban | Total | Depressive disorder | Pediatrician | 323 | 209 | 66.33 | 103.91 | 59.00 | 74.84 | .346 | .557 | 0.08 | -7.32 | [-23.61, 8.96] |
| Male | Total | Urban | Total | Depressive disorder | Psychiatrist/Neurologist | 79 | 39 | 173.82 | 189.70 | 138.33 | 132.84 | .242 | .475 | 0.20 | -35.49 | [-101.90, 30.92] |
| Male | Total | Urban | Total | Depressive disorder | Child and adolescent psychiatrist | 235 | 150 | 90.46 | 138.28 | 44.39 | 67.60 | < .001 | **.001** | **0.40** | -46.07 | [-69.84, -22.31] |
| Male | Total | Urban | Total | Depressive disorder | Psychological psychotherapist | 37 | 26 | 190.84 | 196.43 | 73.81 | 115.67 | .004 | **.037** | **0.70** | -117.03 | [-201.34, -32.72] |
| Male | Total | Urban | Total | Depressive disorder | Child and adolescent psychotherapist | 201 | 129 | 81.07 | 128.85 | 47.22 | 75.68 | .003 | **.029** | **0.30** | -33.86 | [-58.44, -9.28] |
| Female | Total | Rural | Total | Depressive disorder | General practitioner | 539 | 410 | 71.34 | 108.69 | 72.16 | 100.36 | .904 | .939 | 0.01 | 0.82 | [-12.69, 14.33] |
| Female | Total | Rural | Total | Depressive disorder | Pediatrician | 260 | 247 | 81.03 | 113.90 | 63.55 | 86.61 | .052 | .174 | 0.17 | -17.48 | [-35.16, 0.21] |
| Female | Total | Rural | Total | Depressive disorder | Psychiatrist/Neurologist | 67 | 51 | 184.87 | 166.35 | 150.00 | 138.57 | .217 | .450 | 0.22 | -34.87 | [-91.32, 21.59] |
| Female | Total | Rural | Total | Depressive disorder | Child and adolescent psychiatrist | 221 | 217 | 89.96 | 131.04 | 67.21 | 95.83 | .038 | .143 | 0.20 | -22.75 | [-44.28, -1.22] |
| Female | Total | Rural | Total | Depressive disorder | Psychological psychotherapist | 45 | 27 | 153.53 | 154.35 | 126.74 | 168.48 | .503 | .699 | 0.17 | -26.79 | [-103.01, 49.43] |
| Female | Total | Rural | Total | Depressive disorder | Child and adolescent psychotherapist | 270 | 247 | 88.89 | 138.38 | 62.30 | 86.96 | .009 | .056 | 0.23 | -26.58 | [-46.72, -6.45] |
| Male | Total | Rural | Total | Depressive disorder | General practitioner | 216 | 122 | 64.59 | 106.70 | 60.00 | 88.50 | .671 | .788 | 0.05 | -4.59 | [-26.91, 17.72] |
| Male | Total | Rural | Total | Depressive disorder | Pediatrician | 120 | 72 | 80.11 | 110.78 | 62.93 | 81.20 | .219 | .451 | 0.17 | -17.18 | [-46.61, 12.26] |
| Male | Total | Rural | Total | Depressive disorder | Psychiatrist/Neurologist | 28 | 15 | 223.54 | 197.74 | 198.07 | 154.21 | .644 | .777 | 0.14 | -25.47 | [-140.89, 89.95] |
| Male | Total | Rural | Total | Depressive disorder | Child and adolescent psychiatrist | 72 | 48 | 88.92 | 123.69 | 68.85 | 100.54 | .331 | .545 | 0.17 | -20.06 | [-62.08, 21.95] |
| Male | Total | Rural | Total | Depressive disorder | Psychological psychotherapist | 15 | 9 | 147.80 | 168.50 | 83.78 | 79.14 | .222 | .454 | 0.45 | -64.02 | [-181.90, 53.85] |
| Male | Total | Rural | Total | Depressive disorder | Child and adolescent psychotherapist | 79 | 61 | 115.38 | 157.93 | 53.49 | 93.29 | .005 | **.037** | **0.46** | -61.89 | [-106.56, -17.22] |
| Total | 0-9 | Total | Medium SES | Depressive disorder | General practitioner | 40 | 10 | 110.97 | 158.75 | 46.40 | 40.35 | .026 | .115 | 0.45 | -64.57 | [-164.47, 35.32] |
| Total | 0-9 | Total | Medium SES | Depressive disorder | Pediatrician | 70 | 37 | 76.63 | 99.36 | 64.24 | 81.49 | .491 | .692 | 0.13 | -12.39 | [-49.68, 24.91] |
| Total | 0-9 | Total | Medium SES | Depressive disorder | Child and adolescent psychiatrist | 26 | 9 | 78.58 | 98.75 | 95.89 | 137.25 | .734 | .829 | 0.16 | 17.31 | [-65.57, 100.19] |
| Total | 0-9 | Total | Medium SES | Depressive disorder | Child and adolescent psychotherapist | 17 | 5 | 169.76 | 205.93 | 16.60 | 10.24 | .008 | .052 | 0.83 | -153.16 | [-336.88, 30.55] |
| Total | 0-9 | Total | High SES | Depressive disorder | General practitioner | 11 | 6 | 44.09 | 57.68 | 53.17 | 38.81 | .705 | .812 | 0.17 | 9.08 | [-42.80, 60.95] |
| Total | 0-9 | Total | High SES | Depressive disorder | Pediatrician | 15 | 14 | 28.80 | 30.92 | 53.21 | 69.79 | .245 | .476 | 0.46 | 24.41 | [-14.41, 63.23] |
| Total | 0-9 | Total | High SES | Depressive disorder | Child and adolescent psychiatrist | 8 | 6 | 35.00 | 51.13 | 54.00 | 54.15 | .521 | .709 | 0.36 | 19.00 | [-36.48, 74.48] |
| Total | 10-13 | Total | Low SES | Depressive disorder | General practitioner | 72 | 34 | 79.51 | 96.59 | 109.21 | 111.33 | .187 | .410 | 0.29 | 29.69 | [-11.70, 71.09] |
| Total | 10-13 | Total | Low SES | Depressive disorder | Pediatrician | 79 | 61 | 79.48 | 105.87 | 58.93 | 65.30 | .160 | .378 | 0.23 | -20.55 | [-50.78, 9.68] |
| Total | 10-13 | Total | Low SES | Depressive disorder | Child and adolescent psychiatrist | 36 | 27 | 128.47 | 181.77 | 86.33 | 121.87 | .276 | .503 | 0.26 | -42.14 | [-121.49, 37.21] |
| Total | 10-13 | Total | Low SES | Depressive disorder | Child and adolescent psychotherapist | 47 | 32 | 84.47 | 128.77 | 65.81 | 67.80 | .405 | .622 | 0.17 | -18.66 | [-67.36, 30.05] |
| Total | 10-13 | Total | Medium SES | Depressive disorder | General practitioner | 317 | 217 | 89.19 | 118.07 | 72.00 | 100.20 | .071 | .217 | 0.15 | -17.18 | [-36.38, 2.01] |
| Total | 10-13 | Total | Medium SES | Depressive disorder | Pediatrician | 331 | 260 | 71.05 | 102.76 | 54.17 | 73.68 | .020 | .097 | 0.19 | -16.88 | [-31.68, -2.08] |
| Total | 10-13 | Total | Medium SES | Depressive disorder | Psychiatrist/Neurologist | 11 | 10 | 244.27 | 183.30 | 163.90 | 137.58 | .268 | .496 | 0.49 | -80.37 | [-220.17, 59.43] |
| Total | 10-13 | Total | Medium SES | Depressive disorder | Child and adolescent psychiatrist | 212 | 160 | 102.87 | 147.65 | 60.25 | 81.15 | < .001 | **.009** | **0.34** | -42.62 | [-67.98, -17.26] |
| Total | 10-13 | Total | Medium SES | Depressive disorder | Psychological psychotherapist | 12 | 8 | 137.67 | 133.64 | 80.38 | 102.64 | .294 | .516 | 0.47 | -57.29 | [-166.90, 52.31] |
| Total | 10-13 | Total | Medium SES | Depressive disorder | Child and adolescent psychotherapist | 226 | 195 | 88.04 | 139.41 | 63.89 | 87.96 | .032 | .129 | 0.20 | -24.16 | [-46.84, -1.48] |
| Total | 10-13 | Total | High SES | Depressive disorder | General practitioner | 128 | 85 | 119.28 | 152.41 | 105.08 | 117.41 | .445 | .654 | 0.10 | -14.20 | [-52.46, 24.07] |
| Total | 10-13 | Total | High SES | Depressive disorder | Pediatrician | 113 | 110 | 70.40 | 97.20 | 59.95 | 76.61 | .373 | .587 | 0.12 | -10.45 | [-33.46, 12.56] |
| Total | 10-13 | Total | High SES | Depressive disorder | Child and adolescent psychiatrist | 85 | 71 | 66.62 | 106.99 | 47.49 | 79.60 | .203 | .432 | 0.20 | -19.13 | [-49.23, 10.97] |
| Total | 10-13 | Total | High SES | Depressive disorder | Child and adolescent psychotherapist | 72 | 74 | 79.68 | 117.16 | 46.97 | 69.23 | .043 | .153 | 0.34 | -32.71 | [-63.82, -1.59] |
| Total | 14-17 | Total | Low SES | Depressive disorder | General practitioner | 252 | 180 | 87.18 | 125.14 | 80.63 | 114.19 | .572 | .739 | 0.05 | -6.55 | [-29.64, 16.53] |
| Total | 14-17 | Total | Low SES | Depressive disorder | Pediatrician | 116 | 99 | 77.83 | 117.97 | 55.30 | 74.82 | .092 | .261 | 0.22 | -22.52 | [-49.46, 4.41] |
| Total | 14-17 | Total | Low SES | Depressive disorder | Psychiatrist/Neurologist | 32 | 28 | 245.88 | 201.38 | 181.14 | 151.30 | .162 | .381 | 0.36 | -64.73 | [-155.93, 26.47] |
| Total | 14-17 | Total | Low SES | Depressive disorder | Child and adolescent psychiatrist | 98 | 81 | 62.40 | 92.64 | 52.46 | 80.71 | .444 | .654 | 0.11 | -9.94 | [-35.68, 15.80] |
| Total | 14-17 | Total | Low SES | Depressive disorder | Psychological psychotherapist | 11 | 13 | 160.82 | 188.24 | 103.62 | 148.16 | .425 | .644 | 0.34 | -57.20 | [-191.75, 77.35] |
| Total | 14-17 | Total | Low SES | Depressive disorder | Child and adolescent psychotherapist | 115 | 102 | 95.41 | 132.48 | 57.21 | 82.53 | .011 | .064 | 0.34 | -38.20 | [-68.01, -8.39] |
| Total | 14-17 | Total | Medium SES | Depressive disorder | General practitioner | 1,177 | 782 | 80.35 | 122.11 | 70.51 | 99.89 | .051 | .174 | 0.09 | -9.84 | [-20.12, 0.45] |
| Total | 14-17 | Total | Medium SES | Depressive disorder | Pediatrician | 498 | 433 | 80.30 | 118.65 | 63.43 | 87.46 | .013 | .075 | 0.16 | -16.87 | [-30.43, -3.31] |
| Total | 14-17 | Total | Medium SES | Depressive disorder | Psychiatrist/Neurologist | 214 | 116 | 199.13 | 192.32 | 138.91 | 138.82 | .001 | **.017** | **0.34** | -60.22 | [-99.86, -20.57] |
| Total | 14-17 | Total | Medium SES | Depressive disorder | Child and adolescent psychiatrist | 455 | 431 | 88.81 | 128.78 | 63.97 | 96.53 | .001 | **.017** | **0.22** | -24.84 | [-39.89, -9.79] |
| Total | 14-17 | Total | Medium SES | Depressive disorder | Psychological psychotherapist | 115 | 64 | 162.38 | 165.87 | 112.59 | 140.19 | .035 | .137 | 0.32 | -49.79 | [-97.84, -1.74] |
| Total | 14-17 | Total | Medium SES | Depressive disorder | Child and adolescent psychotherapist | 522 | 456 | 76.46 | 121.99 | 57.00 | 89.75 | .004 | .037 | 0.18 | -19.46 | [-33.05, -5.88] |
| Total | 14-17 | Total | High SES | Depressive disorder | General practitioner | 428 | 313 | 71.22 | 108.38 | 67.93 | 84.64 | .644 | .777 | 0.03 | -3.28 | [-17.72, 11.15] |
| Total | 14-17 | Total | High SES | Depressive disorder | Pediatrician | 168 | 164 | 68.36 | 88.83 | 64.32 | 86.00 | .674 | .788 | 0.05 | -4.04 | [-22.85, 14.77] |
| Total | 14-17 | Total | High SES | Depressive disorder | Psychiatrist/Neurologist | 92 | 52 | 157.40 | 168.42 | 146.21 | 128.19 | .655 | .779 | 0.07 | -11.19 | [-63.96, 41.58] |
| Total | 14-17 | Total | High SES | Depressive disorder | Child and adolescent psychiatrist | 205 | 171 | 75.56 | 119.52 | 60.93 | 92.12 | .181 | .408 | 0.14 | -14.63 | [-36.54, 7.28] |
| Total | 14-17 | Total | High SES | Depressive disorder | Psychological psychotherapist | 49 | 36 | 157.98 | 199.96 | 63.83 | 87.60 | .005 | **.037** | **0.58** | -94.15 | [-164.00, -24.29] |
| Total | 14-17 | Total | High SES | Depressive disorder | Child and adolescent psychotherapist | 198 | 170 | 70.50 | 118.94 | 55.87 | 87.81 | .177 | .399 | 0.14 | -14.63 | [-36.29, 7.03] |
| Female | 10-13 | Total | Total | OCD | General practitioner | 20 | 5 | 67.65 | 63.09 | 88.40 | 55.02 | .492 | .692 | 0.34 | 20.75 | [-39.78, 81.28] |
| Female | 10-13 | Total | Total | OCD | Pediatrician | 17 | 14 | 29.59 | 44.26 | 55.21 | 57.80 | .186 | .410 | 0.50 | 25.63 | [-10.30, 61.55] |
| Female | 10-13 | Total | Total | OCD | Child and adolescent psychiatrist | 9 | 6 | 87.89 | 178.87 | 53.67 | 51.50 | .601 | .755 | 0.24 | -34.22 | [-182.88, 114.44] |
| Female | 10-13 | Total | Total | OCD | Child and adolescent psychotherapist | 16 | 8 | 34.12 | 48.02 | 56.62 | 52.26 | .326 | .541 | 0.46 | 22.50 | [-19.43, 64.43] |
| Female | 14-17 | Total | Total | OCD | General practitioner | 30 | 32 | 128.73 | 191.89 | 70.16 | 92.55 | .137 | .346 | 0.39 | -58.58 | [-132.83, 15.68] |
| Female | 14-17 | Total | Total | OCD | Pediatrician | 21 | 20 | 74.71 | 93.49 | 34.40 | 43.85 | .086 | .249 | 0.55 | -40.31 | [-85.39, 4.77] |
| Female | 14-17 | Total | Total | OCD | Psychiatrist/Neurologist | 7 | 6 | 166.57 | 218.72 | 208.83 | 109.17 | .663 | .786 | 0.24 | 42.26 | [-151.31, 235.83] |
| Female | 14-17 | Total | Total | OCD | Child and adolescent psychiatrist | 17 | 20 | 89.18 | 95.64 | 63.10 | 82.64 | .386 | .602 | 0.29 | -26.08 | [-83.50, 31.35] |
| Female | 14-17 | Total | Total | OCD | Child and adolescent psychotherapist | 18 | 26 | 66.28 | 137.71 | 41.88 | 68.17 | .494 | .692 | 0.24 | -24.39 | [-85.80, 37.02] |
| Male | 0-9 | Total | Total | OCD | General practitioner | 8 | 5 | 152.75 | 175.19 | 138.40 | 157.44 | .882 | .925 | 0.08 | -14.35 | [-203.13, 174.43] |
| Male | 0-9 | Total | Total | OCD | Pediatrician | 20 | 14 | 64.20 | 63.49 | 42.79 | 50.98 | .285 | .511 | 0.36 | -21.41 | [-61.52, 18.70] |
| Male | 10-13 | Total | Total | OCD | Pediatrician | 20 | 6 | 39.75 | 83.17 | 44.67 | 44.86 | .853 | .904 | 0.06 | 4.92 | [-65.14, 74.97] |
| Male | 10-13 | Total | Total | OCD | Child and adolescent psychiatrist | 20 | 5 | 36.85 | 50.55 | 19.40 | 22.60 | .268 | .496 | 0.37 | -17.45 | [-63.41, 28.51] |
| Male | 10-13 | Total | Total | OCD | Child and adolescent psychotherapist | 18 | 5 | 122.72 | 159.78 | 80.60 | 129.21 | .561 | .734 | 0.27 | -42.12 | [-195.13, 110.89] |
| Male | 14-17 | Total | Total | OCD | General practitioner | 22 | 21 | 97.91 | 124.65 | 116.29 | 136.30 | .647 | .778 | 0.14 | 18.38 | [-59.63, 96.39] |
| Male | 14-17 | Total | Total | OCD | Pediatrician | 8 | 16 | 148.50 | 161.90 | 65.12 | 81.79 | .207 | .436 | 0.73 | -83.38 | [-179.77, 13.02] |
| Male | 14-17 | Total | Total | OCD | Child and adolescent psychiatrist | 11 | 14 | 47.00 | 51.04 | 36.14 | 46.43 | .589 | .748 | 0.22 | -10.86 | [-49.15, 27.43] |
| Male | 14-17 | Total | Total | OCD | Child and adolescent psychotherapist | 10 | 11 | 64.10 | 92.78 | 91.27 | 92.45 | .510 | .702 | 0.29 | 27.17 | [-52.14, 106.48] |
| Female | Total | Urban | Total | OCD | General practitioner | 34 | 22 | 123.79 | 181.35 | 54.18 | 66.34 | .048 | .165 | 0.47 | -69.61 | [-148.81, 9.59] |
| Female | Total | Urban | Total | OCD | Pediatrician | 30 | 34 | 75.57 | 85.68 | 35.94 | 44.45 | .028 | .119 | 0.59 | -39.63 | [-72.51, -6.75] |
| Female | Total | Urban | Total | OCD | Psychiatrist/Neurologist | 8 | 5 | 204.25 | 192.49 | 225.60 | 113.59 | .807 | .878 | 0.13 | 21.35 | [-166.52, 209.22] |
| Female | Total | Urban | Total | OCD | Child and adolescent psychiatrist | 20 | 21 | 88.05 | 135.75 | 58.67 | 74.90 | .401 | .619 | 0.27 | -29.38 | [-96.06, 37.29] |
| Female | Total | Urban | Total | OCD | Child and adolescent psychotherapist | 25 | 25 | 62.04 | 119.89 | 47.36 | 72.62 | .603 | .755 | 0.15 | -14.68 | [-69.63, 40.27] |
| Male | Total | Urban | Total | OCD | General practitioner | 30 | 18 | 112.23 | 149.43 | 142.39 | 158.99 | .520 | .709 | 0.20 | 30.16 | [-59.27, 119.58] |
| Male | Total | Urban | Total | OCD | Pediatrician | 35 | 28 | 68.74 | 106.48 | 58.50 | 70.70 | .649 | .778 | 0.11 | -10.24 | [-56.15, 35.66] |
| Male | Total | Urban | Total | OCD | Child and adolescent psychiatrist | 20 | 13 | 45.40 | 55.30 | 30.38 | 29.99 | .322 | .538 | 0.32 | -15.02 | [-47.93, 17.90] |
| Male | Total | Urban | Total | OCD | Child and adolescent psychotherapist | 14 | 12 | 138.43 | 179.38 | 110.75 | 110.09 | .635 | .777 | 0.18 | -27.68 | [-144.58, 89.22] |
| Female | Total | Rural | Total | OCD | General practitioner | 18 | 19 | 56.78 | 63.24 | 98.32 | 105.91 | .156 | .372 | 0.47 | 41.54 | [-15.07, 98.15] |
| Female | Total | Rural | Total | OCD | Pediatrician | 12 | 13 | 28.17 | 46.37 | 46.77 | 46.94 | .330 | .544 | 0.40 | 18.60 | [-18.02, 55.22] |
| Female | Total | Rural | Total | OCD | Child and adolescent psychiatrist | 6 | 7 | 91.00 | 102.14 | 51.57 | 78.42 | .461 | .672 | 0.44 | -39.43 | [-137.55, 58.69] |
| Female | Total | Rural | Total | OCD | Child and adolescent psychotherapist | 12 | 11 | 42.17 | 60.54 | 43.36 | 35.22 | .954 | .971 | 0.02 | 1.20 | [-39.80, 42.19] |
| Male | Total | Rural | Total | OCD | General practitioner | 15 | 12 | 76.13 | 92.06 | 56.67 | 44.48 | .479 | .685 | 0.26 | -19.47 | [-76.36, 37.42] |
| Male | Total | Rural | Total | OCD | Pediatrician | 13 | 8 | 66.23 | 78.56 | 33.88 | 37.17 | .220 | .452 | 0.49 | -32.36 | [-90.82, 26.11] |
| Male | Total | Rural | Total | OCD | Child and adolescent psychiatrist | 13 | 7 | 30.08 | 36.64 | 30.14 | 59.29 | .998 | .998 | 0.00 | 0.07 | [-41.70, 41.84] |
| Male | Total | Rural | Total | OCD | Child and adolescent psychotherapist | 15 | 6 | 61.47 | 75.84 | 82.33 | 92.06 | .638 | .777 | 0.26 | 20.87 | [-55.28, 97.01] |
| Total | 0-9 | Total | Medium SES | OCD | General practitioner | 5 | 5 | 155.20 | 209.09 | 75.00 | 92.70 | .468 | .674 | 0.50 | -80.20 | [-280.68, 120.28] |
| Total | 0-9 | Total | Medium SES | OCD | Pediatrician | 16 | 14 | 67.31 | 66.24 | 40.00 | 29.71 | .152 | .366 | 0.52 | -27.31 | [-65.00, 10.37] |
| Total | 10-13 | Total | Medium SES | OCD | Pediatrician | 28 | 14 | 29.54 | 67.13 | 63.64 | 56.67 | .095 | .269 | 0.53 | 34.11 | [-6.90, 75.11] |
| Total | 10-13 | Total | Medium SES | OCD | Child and adolescent psychiatrist | 20 | 6 | 57.50 | 125.56 | 61.83 | 46.38 | .899 | .936 | 0.04 | 4.33 | [-99.40, 108.07] |
| Total | 10-13 | Total | Medium SES | OCD | Child and adolescent psychotherapist | 19 | 10 | 83.37 | 122.60 | 43.80 | 48.00 | .227 | .461 | 0.38 | -39.57 | [-119.10, 39.97] |
| Total | 10-13 | Total | High SES | OCD | General practitioner | 6 | 5 | 62.00 | 81.48 | 31.20 | 37.55 | .436 | .653 | 0.47 | -30.80 | [-108.76, 47.16] |
| Total | 14-17 | Total | Low SES | OCD | General practitioner | 6 | 12 | 248.83 | 195.25 | 120.17 | 154.21 | .196 | .422 | 0.77 | -128.67 | [-293.42, 36.09] |
| Total | 14-17 | Total | Low SES | OCD | Pediatrician | 6 | 6 | 175.00 | 153.30 | 19.17 | 21.77 | .057 | .187 | 1.42 | -155.83 | [-279.73, -31.93] |
| Total | 14-17 | Total | Low SES | OCD | Child and adolescent psychiatrist | 5 | 8 | 144.60 | 100.55 | 35.12 | 26.42 | .076 | .227 | 1.71 | -109.47 | [-181.20, -37.75] |
| Total | 14-17 | Total | Low SES | OCD | Child and adolescent psychotherapist | 5 | 6 | 113.80 | 110.31 | 33.33 | 36.58 | .194 | .417 | 1.03 | -80.47 | [-173.56, 12.62] |
| Total | 14-17 | Total | Medium SES | OCD | General practitioner | 31 | 29 | 94.48 | 151.17 | 77.52 | 112.09 | .622 | .769 | 0.13 | -16.97 | [-84.69, 50.75] |
| Total | 14-17 | Total | Medium SES | OCD | Pediatrician | 20 | 25 | 73.35 | 103.78 | 60.88 | 72.69 | .652 | .778 | 0.14 | -12.47 | [-64.10, 39.16] |
| Total | 14-17 | Total | Medium SES | OCD | Psychiatrist/Neurologist | 6 | 6 | 172.33 | 239.44 | 165.83 | 104.90 | .953 | .971 | 0.04 | -6.50 | [-215.67, 202.67] |
| Total | 14-17 | Total | Medium SES | OCD | Child and adolescent psychiatrist | 12 | 18 | 45.42 | 60.13 | 58.56 | 74.17 | .598 | .753 | 0.19 | 13.14 | [-37.26, 63.54] |
| Total | 14-17 | Total | Medium SES | OCD | Child and adolescent psychotherapist | 16 | 21 | 63.31 | 146.60 | 67.00 | 94.17 | .931 | .955 | 0.03 | 3.69 | [-74.03, 81.41] |
| Total | 14-17 | Total | High SES | OCD | General practitioner | 15 | 12 | 106.27 | 170.79 | 83.08 | 56.19 | .628 | .773 | 0.17 | -23.18 | [-124.24, 77.87] |
| Total | 14-17 | Total | High SES | OCD | Child and adolescent psychiatrist | 11 | 8 | 69.55 | 83.17 | 54.12 | 94.31 | .717 | .818 | 0.18 | -15.42 | [-95.50, 64.66] |
| Total | 14-17 | Total | High SES | OCD | Child and adolescent psychotherapist | 7 | 10 | 36.00 | 42.83 | 48.60 | 58.00 | .615 | .764 | 0.24 | 12.60 | [-38.07, 63.27] |
| Female | 0-9 | Total | Total | PTSD | General practitioner | 46 | 13 | 108.61 | 129.98 | 131.77 | 151.57 | .623 | .769 | 0.17 | 23.16 | [-59.84, 106.16] |
| Female | 0-9 | Total | Total | PTSD | Pediatrician | 112 | 51 | 35.05 | 52.45 | 21.47 | 33.71 | .049 | .170 | 0.29 | -13.58 | [-29.29, 2.12] |
| Female | 0-9 | Total | Total | PTSD | Child and adolescent psychiatrist | 9 | 6 | 72.67 | 104.81 | 22.67 | 42.11 | .226 | .459 | 0.58 | -50.00 | [-139.12, 39.12] |
| Female | 0-9 | Total | Total | PTSD | Child and adolescent psychotherapist | 30 | 9 | 43.90 | 51.71 | 65.67 | 55.75 | .317 | .533 | 0.41 | 21.77 | [-17.42, 60.96] |
| Female | 10-13 | Total | Total | PTSD | General practitioner | 73 | 39 | 118.82 | 147.61 | 106.41 | 125.84 | .641 | .777 | 0.09 | -12.41 | [-67.02, 42.20] |
| Female | 10-13 | Total | Total | PTSD | Pediatrician | 82 | 46 | 49.12 | 64.98 | 42.52 | 50.62 | .525 | .710 | 0.11 | -6.60 | [-28.35, 15.15] |
| Female | 10-13 | Total | Total | PTSD | Child and adolescent psychiatrist | 32 | 18 | 98.84 | 144.11 | 76.83 | 119.66 | .566 | .738 | 0.16 | -22.01 | [-100.52, 56.50] |
| Female | 10-13 | Total | Total | PTSD | Child and adolescent psychotherapist | 40 | 23 | 62.67 | 89.82 | 43.83 | 60.64 | .326 | .541 | 0.23 | -18.85 | [-60.15, 22.45] |
| Female | 14-17 | Total | Total | PTSD | General practitioner | 141 | 110 | 76.05 | 103.87 | 50.58 | 81.75 | .031 | .126 | 0.27 | -25.47 | [-49.11, -1.83] |
| Female | 14-17 | Total | Total | PTSD | Pediatrician | 65 | 57 | 81.57 | 142.94 | 55.00 | 68.80 | .186 | .410 | 0.23 | -26.57 | [-67.29, 14.15] |
| Female | 14-17 | Total | Total | PTSD | Psychiatrist/Neurologist | 21 | 13 | 287.76 | 231.74 | 110.31 | 129.53 | .008 | .052 | 0.89 | -177.45 | [-315.55, -39.36] |
| Female | 14-17 | Total | Total | PTSD | Child and adolescent psychiatrist | 46 | 34 | 76.11 | 109.06 | 50.56 | 59.00 | .183 | .410 | 0.28 | -25.55 | [-66.02, 14.92] |
| Female | 14-17 | Total | Total | PTSD | Psychological psychotherapist | 13 | 5 | 186.00 | 216.07 | 89.60 | 110.05 | .234 | .467 | 0.49 | -96.40 | [-297.57, 104.77] |
| Female | 14-17 | Total | Total | PTSD | Child and adolescent psychotherapist | 55 | 45 | 93.02 | 126.48 | 58.02 | 97.79 | .122 | .312 | 0.31 | -35.00 | [-80.10, 10.11] |
| Male | 0-9 | Total | Total | PTSD | General practitioner | 40 | 24 | 105.17 | 157.40 | 146.50 | 150.48 | .301 | .521 | 0.27 | 41.33 | [-37.05, 119.70] |
| Male | 0-9 | Total | Total | PTSD | Pediatrician | 124 | 68 | 34.95 | 57.46 | 39.68 | 60.14 | .598 | .753 | 0.08 | 4.72 | [-12.55, 22.00] |
| Male | 0-9 | Total | Total | PTSD | Child and adolescent psychiatrist | 16 | 16 | 80.38 | 116.69 | 79.06 | 112.00 | .974 | .983 | 0.01 | -1.31 | [-80.57, 77.94] |
| Male | 0-9 | Total | Total | PTSD | Child and adolescent psychotherapist | 22 | 11 | 106.36 | 187.88 | 67.64 | 78.58 | .412 | .631 | 0.24 | -38.73 | [-155.22, 77.76] |
| Male | 10-13 | Total | Total | PTSD | General practitioner | 33 | 21 | 102.36 | 115.12 | 129.71 | 155.03 | .492 | .692 | 0.21 | 27.35 | [-44.82, 99.52] |
| Male | 10-13 | Total | Total | PTSD | Pediatrician | 52 | 35 | 68.73 | 96.39 | 52.86 | 91.24 | .439 | .653 | 0.17 | -15.87 | [-56.31, 24.57] |
| Male | 10-13 | Total | Total | PTSD | Child and adolescent psychiatrist | 19 | 11 | 92.05 | 156.63 | 136.91 | 117.57 | .383 | .600 | 0.31 | 44.86 | [-62.01, 151.72] |
| Male | 10-13 | Total | Total | PTSD | Child and adolescent psychotherapist | 19 | 12 | 63.16 | 106.48 | 53.17 | 73.56 | .760 | .845 | 0.10 | -9.99 | [-78.90, 58.92] |
| Male | 14-17 | Total | Total | PTSD | General practitioner | 51 | 35 | 85.04 | 129.70 | 74.60 | 115.60 | .697 | .807 | 0.08 | -10.44 | [-63.87, 42.99] |
| Male | 14-17 | Total | Total | PTSD | Pediatrician | 29 | 18 | 60.31 | 95.22 | 51.56 | 82.40 | .741 | .832 | 0.10 | -8.75 | [-62.03, 44.52] |
| Male | 14-17 | Total | Total | PTSD | Child and adolescent psychiatrist | 12 | 16 | 122.00 | 145.31 | 76.44 | 121.39 | .389 | .605 | 0.35 | -45.56 | [-144.39, 53.27] |
| Male | 14-17 | Total | Total | PTSD | Child and adolescent psychotherapist | 17 | 10 | 85.65 | 139.56 | 44.30 | 53.93 | .287 | .513 | 0.36 | -41.35 | [-132.15, 49.45] |
| Female | Total | Urban | Total | PTSD | General practitioner | 150 | 111 | 108.87 | 131.38 | 73.98 | 106.47 | .019 | .094 | 0.29 | -34.88 | [-64.68, -5.09] |
| Female | Total | Urban | Total | PTSD | Pediatrician | 165 | 101 | 45.42 | 69.84 | 38.37 | 51.62 | .347 | .557 | 0.11 | -7.05 | [-22.79, 8.69] |
| Female | Total | Urban | Total | PTSD | Psychiatrist/Neurologist | 14 | 12 | 294.21 | 219.83 | 118.17 | 131.84 | .020 | .097 | 0.95 | -176.05 | [-318.52, -33.57] |
| Female | Total | Urban | Total | PTSD | Child and adolescent psychiatrist | 54 | 41 | 92.67 | 130.92 | 56.93 | 86.86 | .114 | .301 | 0.31 | -35.74 | [-82.05, 10.57] |
| Female | Total | Urban | Total | PTSD | Psychological psychotherapist | 10 | 6 | 170.70 | 147.41 | 117.00 | 119.14 | .441 | .653 | 0.39 | -53.70 | [-193.36, 85.96] |
| Female | Total | Urban | Total | PTSD | Child and adolescent psychotherapist | 75 | 47 | 65.41 | 97.26 | 45.04 | 70.93 | .185 | .410 | 0.23 | -20.37 | [-52.50, 11.75] |
| Male | Total | Urban | Total | PTSD | General practitioner | 69 | 48 | 76.35 | 117.51 | 113.10 | 130.61 | .122 | .312 | 0.30 | 36.76 | [-8.57, 82.08] |
| Male | Total | Urban | Total | PTSD | Pediatrician | 130 | 87 | 49.89 | 84.97 | 43.09 | 71.89 | .527 | .711 | 0.09 | -6.80 | [-28.52, 14.92] |
| Male | Total | Urban | Total | PTSD | Child and adolescent psychiatrist | 31 | 28 | 94.71 | 147.90 | 83.96 | 118.02 | .758 | .844 | 0.08 | -10.75 | [-79.51, 58.02] |
| Male | Total | Urban | Total | PTSD | Child and adolescent psychotherapist | 29 | 19 | 75.21 | 135.07 | 56.47 | 78.97 | .548 | .725 | 0.16 | -18.73 | [-86.06, 48.60] |
| Female | Total | Rural | Total | PTSD | General practitioner | 110 | 51 | 73.30 | 108.68 | 63.04 | 99.73 | .556 | .732 | 0.10 | -10.26 | [-45.44, 24.92] |
| Female | Total | Rural | Total | PTSD | Pediatrician | 94 | 53 | 61.30 | 114.98 | 43.60 | 62.08 | .228 | .461 | 0.18 | -17.69 | [-51.13, 15.74] |
| Female | Total | Rural | Total | PTSD | Child and adolescent psychiatrist | 33 | 17 | 70.12 | 105.96 | 53.18 | 71.03 | .505 | .699 | 0.18 | -16.94 | [-72.97, 39.08] |
| Female | Total | Rural | Total | PTSD | Child and adolescent psychotherapist | 50 | 30 | 80.68 | 110.69 | 69.77 | 99.68 | .651 | .778 | 0.10 | -10.91 | [-59.23, 37.40] |
| Male | Total | Rural | Total | PTSD | General practitioner | 55 | 32 | 120.98 | 151.67 | 106.94 | 154.23 | .682 | .792 | 0.09 | -14.04 | [-80.55, 52.46] |
| Male | Total | Rural | Total | PTSD | Pediatrician | 75 | 34 | 42.28 | 57.82 | 50.79 | 77.82 | .571 | .739 | 0.13 | 8.51 | [-17.68, 34.71] |
| Male | Total | Rural | Total | PTSD | Child and adolescent psychiatrist | 16 | 15 | 97.69 | 124.92 | 109.53 | 117.62 | .788 | .867 | 0.10 | 11.85 | [-73.71, 97.40] |
| Male | Total | Rural | Total | PTSD | Child and adolescent psychotherapist | 29 | 14 | 97.07 | 164.57 | 53.71 | 54.11 | .208 | .436 | 0.31 | -43.35 | [-132.25, 45.54] |
| Total | 0-9 | Total | Low SES | PTSD | General practitioner | 20 | 9 | 65.00 | 68.20 | 212.78 | 176.32 | .038 | .143 | 1.32 | 147.78 | [59.88, 235.68] |
| Total | 0-9 | Total | Low SES | PTSD | Pediatrician | 54 | 31 | 32.39 | 43.00 | 24.77 | 30.08 | .342 | .553 | 0.20 | -7.61 | [-24.76, 9.53] |
| Total | 0-9 | Total | Low SES | PTSD | Child and adolescent psychotherapist | 11 | 5 | 112.64 | 202.80 | 122.80 | 95.87 | .894 | .933 | 0.06 | 10.16 | [-178.95, 199.28] |
| Total | 0-9 | Total | Medium SES | PTSD | General practitioner | 46 | 24 | 116.87 | 144.00 | 115.21 | 126.38 | .961 | .973 | 0.01 | -1.66 | [-69.91, 66.59] |
| Total | 0-9 | Total | Medium SES | PTSD | Pediatrician | 127 | 70 | 39.37 | 59.54 | 34.60 | 54.30 | .570 | .739 | 0.08 | -4.77 | [-21.62, 12.08] |
| Total | 0-9 | Total | Medium SES | PTSD | Child and adolescent psychiatrist | 9 | 15 | 30.78 | 37.15 | 46.20 | 64.05 | .464 | .672 | 0.28 | 15.42 | [-30.69, 61.53] |
| Total | 0-9 | Total | Medium SES | PTSD | Child and adolescent psychotherapist | 30 | 13 | 42.37 | 68.15 | 49.62 | 48.85 | .696 | .807 | 0.11 | 7.25 | [-33.83, 48.33] |
| Total | 0-9 | Total | High SES | PTSD | Pediatrician | 55 | 18 | 27.47 | 54.58 | 33.50 | 66.92 | .732 | .829 | 0.10 | 6.03 | [-24.72, 36.78] |
| Total | 10-13 | Total | Low SES | PTSD | General practitioner | 22 | 18 | 141.45 | 160.65 | 108.78 | 135.93 | .490 | .692 | 0.22 | -32.68 | [-126.18, 60.82] |
| Total | 10-13 | Total | Low SES | PTSD | Pediatrician | 23 | 24 | 48.65 | 75.37 | 45.29 | 87.04 | .888 | .928 | 0.04 | -3.36 | [-50.00, 43.28] |
| Total | 10-13 | Total | Low SES | PTSD | Child and adolescent psychiatrist | 10 | 8 | 149.90 | 192.23 | 111.50 | 126.30 | .618 | .766 | 0.23 | -38.40 | [-193.31, 116.51] |
| Total | 10-13 | Total | Low SES | PTSD | Child and adolescent psychotherapist | 11 | 10 | 39.00 | 46.66 | 51.00 | 52.92 | .590 | .748 | 0.24 | 12.00 | [-30.59, 54.59] |
| Total | 10-13 | Total | Medium SES | PTSD | General practitioner | 64 | 35 | 106.97 | 143.12 | 117.00 | 138.90 | .735 | .829 | 0.07 | 10.03 | [-48.34, 68.40] |
| Total | 10-13 | Total | Medium SES | PTSD | Pediatrician | 89 | 47 | 59.36 | 83.28 | 52.15 | 67.88 | .588 | .748 | 0.09 | -7.21 | [-34.90, 20.47] |
| Total | 10-13 | Total | Medium SES | PTSD | Child and adolescent psychiatrist | 32 | 15 | 86.62 | 141.59 | 131.27 | 126.58 | .287 | .513 | 0.33 | 44.64 | [-39.44, 128.72] |
| Total | 10-13 | Total | Medium SES | PTSD | Child and adolescent psychotherapist | 43 | 19 | 72.65 | 105.66 | 42.00 | 72.00 | .190 | .413 | 0.32 | -30.65 | [-82.92, 21.61] |
| Total | 10-13 | Total | High SES | PTSD | General practitioner | 20 | 7 | 104.70 | 87.39 | 117.29 | 140.78 | .831 | .896 | 0.12 | 12.59 | [-75.87, 101.04] |
| Total | 10-13 | Total | High SES | PTSD | Pediatrician | 22 | 10 | 54.55 | 65.65 | 26.80 | 31.42 | .117 | .306 | 0.48 | -27.75 | [-70.77, 15.28] |
| Total | 10-13 | Total | High SES | PTSD | Child and adolescent psychiatrist | 9 | 6 | 71.22 | 108.75 | 4.67 | 4.03 | .104 | .284 | 0.78 | -66.56 | [-154.72, 21.61] |
| Total | 10-13 | Total | High SES | PTSD | Child and adolescent psychotherapist | 5 | 6 | 30.80 | 56.05 | 56.33 | 65.48 | .506 | .699 | 0.42 | 25.53 | [-47.42, 98.49] |
| Total | 14-17 | Total | Low SES | PTSD | General practitioner | 42 | 22 | 63.21 | 70.78 | 85.27 | 139.72 | .493 | .692 | 0.22 | 22.06 | [-29.33, 73.45] |
| Total | 14-17 | Total | Low SES | PTSD | Pediatrician | 17 | 16 | 82.47 | 168.49 | 62.69 | 91.20 | .676 | .790 | 0.14 | -19.78 | [-113.08, 73.51] |
| Total | 14-17 | Total | Low SES | PTSD | Child and adolescent psychiatrist | 11 | 9 | 76.73 | 77.61 | 52.56 | 74.94 | .490 | .692 | 0.32 | -24.17 | [-91.51, 43.17] |
| Total | 14-17 | Total | Low SES | PTSD | Child and adolescent psychotherapist | 22 | 9 | 114.50 | 131.68 | 71.11 | 90.82 | .305 | .524 | 0.36 | -43.39 | [-137.84, 51.06] |
| Total | 14-17 | Total | Medium SES | PTSD | General practitioner | 115 | 97 | 74.46 | 108.27 | 50.04 | 72.82 | .052 | .176 | 0.26 | -24.42 | [-49.75, 0.91] |
| Total | 14-17 | Total | Medium SES | PTSD | Pediatrician | 62 | 49 | 67.79 | 118.71 | 52.06 | 69.34 | .385 | .602 | 0.16 | -15.73 | [-53.20, 21.74] |
| Total | 14-17 | Total | Medium SES | PTSD | Psychiatrist/Neurologist | 18 | 12 | 275.83 | 253.23 | 131.83 | 138.69 | .055 | .183 | 0.67 | -144.00 | [-301.50, 13.50] |
| Total | 14-17 | Total | Medium SES | PTSD | Child and adolescent psychiatrist | 39 | 34 | 84.85 | 128.45 | 64.32 | 91.88 | .431 | .649 | 0.18 | -20.52 | [-72.46, 31.41] |
| Total | 14-17 | Total | Medium SES | PTSD | Psychological psychotherapist | 10 | 6 | 116.70 | 98.71 | 135.00 | 211.91 | .849 | .903 | 0.12 | 18.30 | [-132.85, 169.45] |
| Total | 14-17 | Total | Medium SES | PTSD | Child and adolescent psychotherapist | 38 | 36 | 93.82 | 141.35 | 60.47 | 101.91 | .247 | .477 | 0.27 | -33.34 | [-89.76, 23.07] |
| Total | 14-17 | Total | High SES | PTSD | General practitioner | 35 | 26 | 109.77 | 150.18 | 55.58 | 101.36 | .098 | .272 | 0.41 | -54.19 | [-121.04, 12.65] |
| Total | 14-17 | Total | High SES | PTSD | Pediatrician | 15 | 10 | 96.40 | 132.50 | 50.90 | 51.20 | .244 | .476 | 0.42 | -45.50 | [-132.09, 41.09] |
| Total | 14-17 | Total | High SES | PTSD | Child and adolescent psychiatrist | 8 | 7 | 101.50 | 117.99 | 40.29 | 51.39 | .216 | .450 | 0.66 | -61.21 | [-155.91, 33.49] |
| Total | 14-17 | Total | High SES | PTSD | Child and adolescent psychotherapist | 12 | 10 | 40.67 | 52.13 | 23.70 | 25.33 | .334 | .547 | 0.40 | -16.97 | [-52.41, 18.48] |

*Note.* Cohen's |d|: small effect: ≥0.2, medium effect: ≥0.5, large effect: ≥0.8; n, sample size; *M*, mean; *SD*, standard deviation; *ΔM*, mean difference (intra-COVID-19 – pre-COVID-19); Δ%; *p.adj*, Benjamini-Hochberg adjusted *p*-values, Benjamini-Hochberg procedure was applied with the following parameters: i = rank of p-value, m = 668 = total number of tests and Q = 0.05 = false discovery rate, using the formula for critical value = (i/m))* Q; only comparisons with *n* >5 are included and only comparisons with *p.adj* < .05 and |d| ≥ 0.2 are highlighted.

^ölasklökda^
