# Supplementary material for: Continuity of care: time to first outpatient appointment after child and adolescent psychiatric hospital stays in Germany
Source: BMC Health Serv Res. 2026 Mar 13;26:486. doi: 10.1186/s12913-026-14322-7 (PMC13063905; doi:10.1186/s12913-026-14322-7)
Supplement: Supplementary file 2 — Supplementary Material 2: Duration of inpatient stay (LOS) of children and adolescents with psychiatric disorders before vs. during the COVID-19 pandemic, by sex, age, residency, status and disorder. [file 12913_2026_14322_MOESM3_ESM.docx]

**Supplemental table 2**. Duration of inpatient stay^1^ (LOS) of children and adolescents with psychiatric disorders before vs. during the COVID-19 pandemic, by sex, age, residency, status and disorder

| Sex | Age | Residency | Status | ICD | *2019* | *2021* | *2019* | | *2021* | | *p* | *p.adj* | *\|d\|* | *ΔM* | *95% CI ΔM* |
| --- | --- | --- | --- | --- | --- | --- | --- | --- | --- | --- | --- | --- | --- | --- | --- |
|  |  |  |  |  | *n* | *n* | *M* | *SD* | *M* | *SD* |  |  |  |  |  |
| Total | Total | Total | Total | Anorexia nervosa | 413 | 421 | 88.67 | 95.61 | 74.44 | 73.76 | .016 | .118 | 0.17 | -14.23 | [-25.81, -2.66] |
| Total | Total | Total | Total | Anxiety disorder | 5,309 | 3,532 | 33.03 | 49.01 | 29.91 | 43.14 | .002 | .050 | 0.07 | -3.12 | [-5.11, -1.13] |
| Total | Total | Total | Total | Depressive disorder | 3,260 | 2,438 | 53.76 | 64.29 | 46.96 | 55.17 | < .001 | .004 | 0.11 | -6.80 | [-9.98, -3.62] |
| Total | Total | Total | Total | OCD | 167 | 137 | 49.72 | 55.09 | 55.41 | 57.62 | .383 | .659 | 0.10 | 5.69 | [-7.02, 18.40] |
| Total | Total | Total | Total | PTSD | 710 | 453 | 33.07 | 56.53 | 37.61 | 63.85 | .217 | .482 | 0.08 | 4.54 | [-2.47, 11.55] |
| Female | Total | Total | Total | Anorexia nervosa | 387 | 389 | 90.98 | 96.78 | 76.34 | 74.62 | .019 | .129 | 0.17 | -14.64 | [-26.80, -2.49] |
| Male | Total | Total | Total | Anorexia nervosa | 26 | 32 | 54.27 | 68.76 | 51.31 | 58.43 | .863 | .932 | 0.05 | -2.96 | [-35.69, 29.78] |
| Total | 0-9 | Total | Total | Anorexia nervosa | 7 | 9 | 46.86 | 79.93 | 26.11 | 41.91 | .550 | .803 | 0.34 | -20.75 | [-81.16, 39.67] |
| Total | 10-13 | Total | Total | Anorexia nervosa | 120 | 141 | 95.62 | 106.59 | 70.38 | 73.01 | .029 | .157 | 0.28 | -25.23 | [-47.14, -3.32] |
| Total | 14-17 | Total | Total | Anorexia nervosa | 286 | 271 | 86.78 | 90.95 | 78.15 | 74.47 | .220 | .485 | 0.10 | -8.63 | [-22.48, 5.22] |
| Total | Total | Urban | Total | Anorexia nervosa | 286 | 127 | 86.87 | 92.68 | 77.71 | 76.35 | .294 | .575 | 0.10 | -9.17 | [-27.56, 9.22] |
| Total | Total | Rural | Total | Anorexia nervosa | 296 | 125 | 92.72 | 102.19 | 66.70 | 66.89 | .002 | .050 | 0.28 | -26.02 | [-45.49, -6.55] |
| Total | Total | Total | Low SES | Anorexia nervosa | 53 | 47 | 72.49 | 90.80 | 65.15 | 71.24 | .652 | .889 | 0.09 | -7.34 | [-39.62, 24.94] |
| Total | Total | Total | Medium SES | Anorexia nervosa | 253 | 259 | 86.36 | 90.44 | 70.71 | 69.61 | .029 | .157 | 0.19 | -15.65 | [-29.61, -1.69] |
| Total | Total | Total | High SES | Anorexia nervosa | 107 | 115 | 102.16 | 108.24 | 86.63 | 82.54 | .233 | .496 | 0.16 | -15.52 | [-40.74, 9.69] |
| Female | Total | Total | Total | Anxiety disorder | 2,617 | 1,820 | 33.21 | 51.06 | 30.53 | 45.40 | .066 | .262 | 0.05 | -2.68 | [-5.60, 0.24] |
| Male | Total | Total | Total | Anxiety disorder | 2,692 | 1,712 | 32.86 | 46.94 | 29.26 | 40.60 | .007 | .072 | 0.08 | -3.60 | [-6.30, -0.90] |
| Total | 0-9 | Total | Total | Anxiety disorder | 2,505 | 1,437 | 24.93 | 41.82 | 22.50 | 35.13 | .052 | .226 | 0.06 | -2.42 | [-4.99, 0.14] |
| Total | 10-13 | Total | Total | Anxiety disorder | 1,607 | 1,109 | 41.14 | 53.54 | 35.28 | 45.09 | .002 | .050 | 0.12 | -5.86 | [-9.71, -2.02] |
| Total | 14-17 | Total | Total | Anxiety disorder | 1,197 | 986 | 39.10 | 53.73 | 34.68 | 49.49 | .046 | .212 | 0.09 | -4.42 | [-8.79, -0.05] |
| Total | Total | Urban | Total | Anxiety disorder | 3,713 | 2,439 | 32.88 | 48.87 | 29.82 | 42.99 | .010 | .078 | 0.07 | -3.07 | [-5.45, -0.68] |
| Total | Total | Rural | Total | Anxiety disorder | 1,596 | 1,092 | 33.37 | 49.36 | 30.15 | 43.50 | .075 | .280 | 0.07 | -3.22 | [-6.84, 0.40] |
| Total | Total | Total | Low SES | Anxiety disorder | 852 | 573 | 29.97 | 42.03 | 30.88 | 40.87 | .684 | .898 | 0.02 | 0.91 | [-3.49, 5.31] |
| Total | Total | Total | Medium SES | Anxiety disorder | 3,371 | 2,261 | 34.18 | 49.78 | 30.62 | 44.29 | .005 | .058 | 0.07 | -3.56 | [-6.10, -1.02] |
| Total | Total | Total | High SES | Anxiety disorder | 1,086 | 697 | 31.86 | 51.53 | 26.85 | 41.05 | .023 | .140 | 0.11 | -5.01 | [-9.55, -0.47] |
| Female | Total | Total | Total | Depressive disorder | 2,229 | 1,840 | 57.16 | 69.24 | 49.29 | 57.56 | < .001 | .009 | 0.12 | -7.87 | [-11.25, -4.48] |
| Male | Total | Total | Total | Depressive disorder | 1,031 | 598 | 46.41 | 51.28 | 39.79 | 46.35 | .008 | .072 | 0.13 | -6.62 | [-11.61, -1.63] |
| Total | 0-9 | Total | Total | Depressive disorder | 133 | 62 | 46.02 | 54.48 | 39.27 | 46.95 | .377 | .656 | 0.13 | -6.74 | [-22.48, 9.00] |
| Total | 10-13 | Total | Total | Depressive disorder | 887 | 687 | 54.91 | 65.52 | 45.43 | 54.08 | .002 | .050 | 0.16 | -9.48 | [-15.54, -3.42] |
| Total | 14-17 | Total | Total | Depressive disorder | 2,240 | 1,689 | 53.76 | 64.33 | 47.87 | 55.87 | .002 | .050 | 0.10 | -5.90 | [-9.74, -2.05] |
| Total | Total | Urban | Total | Depressive disorder | 2,278 | 1,734 | 52.02 | 63.18 | 45.73 | 54.13 | .001 | .050 | 0.11 | -6.29 | [-10.00, -2.58] |
| Total | Total | Rural | Total | Depressive disorder | 980 | 704 | 57.79 | 66.68 | 50.01 | 57.55 | .011 | .084 | 0.12 | -7.78 | [-13.88, -1.68] |
| Total | Total | Total | Low SES | Depressive disorder | 454 | 326 | 47.45 | 58.98 | 42.51 | 46.66 | .192 | .432 | 0.09 | -4.94 | [-12.65, 2.77] |
| Total | Total | Total | Medium SES | Depressive disorder | 2,064 | 1,515 | 53.38 | 64.02 | 47.51 | 55.28 | .003 | .052 | 0.10 | -5.87 | [-9.88, -1.86] |
| Total | Total | Total | High SES | Depressive disorder | 740 | 597 | 58.66 | 67.81 | 47.99 | 59.00 | .002 | .050 | 0.17 | -10.66 | [-17.56, -3.76] |
| Female | Total | Total | Total | OCD | 84 | 77 | 49.57 | 57.30 | 54.12 | 50.62 | .594 | .845 | 0.08 | 4.55 | [-12.22, 21.31] |
| Male | Total | Total | Total | OCD | 83 | 60 | 49.87 | 53.10 | 57.07 | 65.93 | .487 | .775 | 0.12 | 7.20 | [-12.33, 26.73] |
| Total | 0-9 | Total | Total | OCD | 30 | 31 | 49.57 | 66.81 | 31.45 | 49.68 | .236 | .496 | 0.31 | -18.12 | [-47.59, 11.36] |
| Total | 10-13 | Total | Total | OCD | 70 | 31 | 43.66 | 51.57 | 46.35 | 39.48 | .775 | .898 | 0.06 | 2.70 | [-17.70, 23.09] |
| Total | 14-17 | Total | Total | OCD | 67 | 75 | 56.12 | 53.02 | 69.05 | 63.29 | .187 | .432 | 0.22 | 12.93 | [-6.40, 32.26] |
| Total | Total | Urban | Total | OCD | 111 | 94 | 50.01 | 53.27 | 57.72 | 61.73 | .344 | .620 | 0.13 | 7.71 | [-8.03, 23.46] |
| Total | Total | Rural | Total | OCD | 56 | 43 | 49.14 | 59.02 | 50.35 | 47.69 | .911 | .935 | 0.02 | 1.21 | [-20.42, 22.83] |
| Total | Total | Total | Low SES | OCD | 18 | 24 | 55.61 | 57.77 | 45.54 | 65.79 | .602 | .850 | 0.16 | -10.07 | [-48.27, 28.13] |
| Total | Total | Total | Medium SES | OCD | 113 | 79 | 43.76 | 51.54 | 55.86 | 50.93 | .109 | .332 | 0.24 | 12.10 | [-2.64, 26.84] |
| Total | Total | Total | High SES | OCD | 36 | 34 | 65.47 | 62.33 | 61.32 | 66.53 | .789 | .898 | 0.06 | -4.15 | [-34.34, 26.04] |
| Female | Total | Total | Total | PTSD | 424 | 276 | 34.06 | 58.84 | 38.91 | 63.04 | .308 | .578 | 0.08 | 4.85 | [-4.33, 14.03] |
| Male | Total | Total | Total | PTSD | 286 | 177 | 31.61 | 52.99 | 35.59 | 65.22 | .494 | .775 | 0.07 | 3.98 | [-6.88, 14.85] |
| Total | 0-9 | Total | Total | PTSD | 276 | 141 | 25.67 | 56.50 | 35.81 | 64.39 | .114 | .332 | 0.17 | 10.14 | [-1.89, 22.17] |
| Total | 10-13 | Total | Total | PTSD | 198 | 118 | 38.93 | 55.93 | 34.86 | 53.95 | .523 | .795 | 0.07 | -4.07 | [-16.66, 8.51] |
| Total | 14-17 | Total | Total | PTSD | 236 | 194 | 36.81 | 56.36 | 40.60 | 68.99 | .539 | .797 | 0.06 | 3.79 | [-8.06, 15.64] |
| Total | Total | Urban | Total | PTSD | 444 | 313 | 34.68 | 60.61 | 36.16 | 60.15 | .741 | .898 | 0.02 | 1.48 | [-7.26, 10.22] |
| Total | Total | Rural | Total | PTSD | 266 | 140 | 30.38 | 48.97 | 40.86 | 71.55 | .122 | .340 | 0.18 | 10.48 | [-1.34, 22.30] |
| Total | Total | Total | Low SES | PTSD | 146 | 95 | 31.58 | 44.57 | 32.98 | 56.10 | .838 | .922 | 0.03 | 1.40 | [-11.37, 14.17] |
| Total | Total | Total | Medium SES | PTSD | 424 | 288 | 31.63 | 52.85 | 39.21 | 60.89 | .086 | .304 | 0.13 | 7.58 | [-0.84, 16.00] |
| Total | Total | Total | High SES | PTSD | 140 | 70 | 38.99 | 75.39 | 37.31 | 83.40 | .887 | .935 | 0.02 | -1.68 | [-24.10, 20.74] |
| Female | 0-9 | Total | Total | Anorexia nervosa | 5 | 6 | 54.20 | 94.98 | 37.33 | 48.52 | .734 | .898 | 0.23 | -16.87 | [-103.41, 69.68] |
| Female | 10-13 | Total | Total | Anorexia nervosa | 109 | 130 | 98.41 | 109.65 | 71.35 | 74.43 | .030 | .157 | 0.29 | -27.06 | [-50.52, -3.60] |
| Female | 14-17 | Total | Total | Anorexia nervosa | 273 | 253 | 88.69 | 91.29 | 79.83 | 75.03 | .223 | .486 | 0.11 | -8.86 | [-23.21, 5.48] |
| Male | 10-13 | Total | Total | Anorexia nervosa | 11 | 11 | 67.91 | 66.42 | 58.91 | 54.86 | .733 | .898 | 0.15 | -9.00 | [-59.91, 41.91] |
| Male | 14-17 | Total | Total | Anorexia nervosa | 13 | 18 | 46.69 | 75.65 | 54.61 | 63.29 | .761 | .898 | 0.12 | 7.92 | [-41.07, 56.91] |
| Female | Total | Urban | Total | Anorexia nervosa | 266 | 121 | 88.83 | 93.96 | 79.50 | 77.34 | .305 | .578 | 0.10 | -9.33 | [-28.49, 9.82] |
| Male | Total | Urban | Total | Anorexia nervosa | 20 | 6 | 60.85 | 70.17 | 57.42 | 61.77 | .911 | .935 | 0.05 | -3.43 | [-65.93, 59.06] |
| Female | Total | Rural | Total | Anorexia nervosa | 272 | 117 | 95.71 | 102.95 | 69.00 | 67.63 | .003 | .050 | 0.28 | -26.71 | [-47.03, -6.39] |
| Male | Total | Rural | Total | Anorexia nervosa | 24 | 8 | 32.33 | 64.67 | 33.00 | 45.53 | .975 | .979 | 0.01 | 0.67 | [-47.94, 49.27] |
| Total | 10-13 | Total | Low SES | Anorexia nervosa | 14 | 16 | 75.14 | 90.90 | 65.00 | 81.61 | .752 | .898 | 0.12 | -10.14 | [-71.86, 51.58] |
| Total | 10-13 | Total | Medium SES | Anorexia nervosa | 75 | 84 | 81.87 | 88.71 | 61.38 | 66.64 | .105 | .332 | 0.26 | -20.49 | [-44.72, 3.75] |
| Total | 10-13 | Total | High SES | Anorexia nervosa | 31 | 41 | 138.13 | 139.78 | 90.93 | 79.56 | .099 | .323 | 0.43 | -47.20 | [-98.28, 3.88] |
| Total | 14-17 | Total | Low SES | Anorexia nervosa | 38 | 30 | 73.34 | 92.46 | 65.90 | 67.75 | .703 | .898 | 0.09 | -7.44 | [-46.94, 32.06] |
| Total | 14-17 | Total | Medium SES | Anorexia nervosa | 174 | 172 | 88.44 | 91.50 | 76.26 | 70.87 | .167 | .402 | 0.15 | -12.18 | [-29.44, 5.08] |
| Total | 14-17 | Total | High SES | Anorexia nervosa | 74 | 69 | 89.77 | 89.49 | 88.19 | 85.18 | .914 | .935 | 0.02 | -1.58 | [-30.26, 27.10] |
| Female | 0-9 | Total | Total | Anxiety disorder | 988 | 556 | 20.91 | 39.87 | 18.31 | 30.01 | .147 | .383 | 0.07 | -2.61 | [-6.41, 1.20] |
| Female | 10-13 | Total | Total | Anxiety disorder | 840 | 607 | 42.10 | 57.67 | 34.42 | 46.24 | .005 | .058 | 0.14 | -7.69 | [-13.24, -2.14] |
| Female | 14-17 | Total | Total | Anxiety disorder | 789 | 657 | 39.13 | 53.03 | 37.28 | 52.84 | .508 | .792 | 0.03 | -1.85 | [-7.33, 3.63] |
| Male | 0-9 | Total | Total | Anxiety disorder | 1,517 | 502 | 27.54 | 42.85 | 25.15 | 37.79 | .236 | .496 | 0.06 | -2.39 | [-6.59, 1.82] |
| Male | 10-13 | Total | Total | Anxiety disorder | 767 | 502 | 40.09 | 48.62 | 36.32 | 43.68 | .152 | .391 | 0.08 | -3.76 | [-9.02, 1.50] |
| Male | 14-17 | Total | Total | Anxiety disorder | 408 | 329 | 39.03 | 55.14 | 29.47 | 41.59 | .007 | .072 | 0.19 | -9.56 | [-16.76, -2.36] |
| Female | Total | Urban | Total | Anxiety disorder | 1,848 | 1,260 | 33.52 | 50.19 | 30.79 | 45.67 | .116 | .332 | 0.06 | -2.73 | [-6.20, 0.73] |
| Male | Total | Urban | Total | Anxiety disorder | 1,865 | 1,179 | 32.25 | 47.53 | 28.78 | 39.91 | .030 | .157 | 0.08 | -3.47 | [-6.74, -0.21] |
| Female | Total | Rural | Total | Anxiety disorder | 769 | 559 | 32.46 | 53.11 | 29.99 | 44.84 | .360 | .644 | 0.05 | -2.47 | [-7.89, 2.96] |
| Male | Total | Rural | Total | Anxiety disorder | 827 | 533 | 34.22 | 45.60 | 30.32 | 42.08 | .107 | .332 | 0.09 | -3.90 | [-8.72, 0.92] |
| Female | Total | Total | Low SES | Anxiety disorder | 397 | 319 | 28.15 | 43.10 | 31.30 | 43.08 | .332 | .608 | 0.07 | 3.15 | [-3.20, 9.50] |
| Male | Total | Total | Low SES | Anxiety disorder | 455 | 254 | 31.56 | 41.05 | 30.35 | 37.99 | .695 | .898 | 0.03 | -1.20 | [-7.34, 4.94] |
| Female | Total | Total | Medium SES | Anxiety disorder | 1,675 | 1,166 | 35.05 | 52.32 | 31.62 | 47.32 | .069 | .269 | 0.07 | -3.43 | [-7.19, 0.34] |
| Male | Total | Total | Medium SES | Anxiety disorder | 1,696 | 1,095 | 33.33 | 47.14 | 29.56 | 40.82 | .025 | .149 | 0.08 | -3.76 | [-7.16, -0.36] |
| Female | Total | Total | High SES | Anxiety disorder | 545 | 334 | 31.24 | 52.22 | 26.06 | 40.34 | .100 | .323 | 0.11 | -5.18 | [-11.72, 1.36] |
| Male | Total | Total | High SES | Anxiety disorder | 541 | 363 | 32.49 | 50.87 | 27.58 | 41.74 | .113 | .332 | 0.10 | -4.91 | [-11.21, 1.40] |
| Total | 0-9 | Total | Low SES | Anxiety disorder | 393 | 217 | 25.80 | 38.16 | 27.80 | 36.91 | .528 | .795 | 0.05 | 1.99 | [-4.26, 8.25] |
| Total | 0-9 | Total | Medium SES | Anxiety disorder | 1,575 | 877 | 26.18 | 43.46 | 22.74 | 35.37 | .034 | .168 | 0.08 | -3.44 | [-6.81, -0.08] |
| Total | 0-9 | Total | High SES | Anxiety disorder | 537 | 343 | 20.61 | 39.17 | 18.56 | 32.93 | .404 | .674 | 0.06 | -2.05 | [-7.04, 2.95] |
| Total | 10-13 | Total | Low SES | Anxiety disorder | 260 | 192 | 34.83 | 42.24 | 33.30 | 46.57 | .720 | .898 | 0.03 | -1.53 | [-9.76, 6.70] |
| Total | 10-13 | Total | Medium SES | Anxiety disorder | 1,040 | 733 | 41.53 | 54.63 | 35.33 | 43.80 | .008 | .072 | 0.12 | -6.20 | [-10.97, -1.43] |
| Total | 10-13 | Total | High SES | Anxiety disorder | 307 | 184 | 45.17 | 57.86 | 37.14 | 48.62 | .100 | .323 | 0.15 | -8.03 | [-18.00, 1.95] |
| Total | 14-17 | Total | Low SES | Anxiety disorder | 199 | 164 | 31.84 | 48.03 | 32.12 | 38.64 | .951 | .968 | 0.01 | 0.28 | [-8.83, 9.38] |
| Total | 14-17 | Total | Medium SES | Anxiety disorder | 756 | 651 | 40.74 | 52.59 | 35.95 | 53.26 | .091 | .316 | 0.09 | -4.79 | [-10.33, 0.76] |
| Total | 14-17 | Total | High SES | Anxiety disorder | 242 | 170 | 39.94 | 60.96 | 32.44 | 43.58 | .146 | .383 | 0.14 | -7.51 | [-18.19, 3.18] |
| Female | 0-9 | Total | Total | Depressive disorder | 41 | 23 | 27.76 | 41.62 | 31.91 | 40.31 | .697 | .898 | 0.10 | 4.16 | [-16.86, 25.17] |
| Female | 10-13 | Total | Total | Depressive disorder | 616 | 540 | 57.26 | 69.36 | 47.14 | 56.81 | .006 | .070 | 0.16 | -10.12 | [-16.52, -3.73] |
| Female | 14-17 | Total | Total | Depressive disorder | 1,572 | 1,277 | 57.89 | 69.62 | 50.52 | 58.09 | .002 | .050 | 0.11 | -7.37 | [-12.15, -2.59] |
| Male | 0-9 | Total | Total | Depressive disorder | 92 | 39 | 54.15 | 57.68 | 43.62 | 50.46 | .299 | .577 | 0.19 | -10.54 | [-31.38, 10.31] |
| Male | 10-13 | Total | Total | Depressive disorder | 271 | 147 | 49.56 | 55.54 | 39.15 | 42.14 | .032 | .164 | 0.20 | -10.41 | [-20.70, -0.12] |
| Male | 14-17 | Total | Total | Depressive disorder | 668 | 412 | 44.06 | 48.37 | 39.66 | 47.47 | .142 | .377 | 0.09 | -4.41 | [-10.30, 1.49] |
| Female | Total | Urban | Total | Depressive disorder | 1,547 | 1,308 | 54.96 | 68.03 | 48.05 | 56.52 | .003 | .050 | 0.11 | -6.92 | [-11.55, -2.28] |
| Male | Total | Urban | Total | Depressive disorder | 731 | 426 | 45.78 | 50.91 | 38.60 | 45.38 | .013 | .101 | 0.15 | -7.18 | [-13.03, -1.33] |
| Female | Total | Rural | Total | Depressive disorder | 681 | 532 | 62.21 | 71.73 | 52.36 | 59.99 | .009 | .078 | 0.15 | -9.86 | [-17.44, -2.28] |
| Male | Total | Rural | Total | Depressive disorder | 299 | 172 | 47.70 | 52.16 | 42.73 | 48.68 | .300 | .577 | 0.10 | -4.97 | [-14.52, 4.58] |
| Female | Total | Total | Low SES | Depressive disorder | 302 | 248 | 49.08 | 60.54 | 43.01 | 47.64 | .189 | .432 | 0.11 | -6.07 | [-15.32, 3.19] |
| Male | Total | Total | Low SES | Depressive disorder | 152 | 78 | 44.23 | 55.80 | 40.94 | 43.64 | .624 | .865 | 0.06 | -3.29 | [-17.49, 10.90] |
| Female | Total | Total | Medium SES | Depressive disorder | 1,417 | 1,145 | 57.23 | 69.45 | 49.74 | 57.45 | .003 | .050 | 0.12 | -7.49 | [-12.50, -2.47] |
| Male | Total | Total | Medium SES | Depressive disorder | 647 | 370 | 44.95 | 49.12 | 40.62 | 47.36 | .167 | .402 | 0.09 | -4.33 | [-10.53, 1.86] |
| Female | Total | Total | High SES | Depressive disorder | 509 | 447 | 61.85 | 73.10 | 51.64 | 62.53 | .020 | .132 | 0.15 | -10.22 | [-18.90, -1.53] |
| Male | Total | Total | High SES | Depressive disorder | 231 | 150 | 51.61 | 53.82 | 37.14 | 45.39 | .005 | .058 | 0.29 | -14.47 | [-24.88, -4.05] |
| Total | 0-9 | Total | Low SES | Depressive disorder | 18 | 5 | 48.50 | 46.61 | 44.40 | 41.51 | .855 | .928 | 0.09 | -4.10 | [-49.37, 41.17] |
| Total | 0-9 | Total | Medium SES | Depressive disorder | 89 | 40 | 43.63 | 54.08 | 29.80 | 37.04 | .094 | .323 | 0.28 | -13.83 | [-32.29, 4.63] |
| Total | 0-9 | Total | High SES | Depressive disorder | 26 | 17 | 52.46 | 61.90 | 60.06 | 62.76 | .699 | .898 | 0.12 | 7.60 | [-30.45, 45.64] |
| Total | 10-13 | Total | Low SES | Depressive disorder | 134 | 86 | 50.55 | 55.74 | 41.01 | 49.04 | .184 | .431 | 0.18 | -9.54 | [-23.96, 4.87] |
| Total | 10-13 | Total | Medium SES | Depressive disorder | 556 | 430 | 54.38 | 66.24 | 45.56 | 52.67 | .020 | .132 | 0.15 | -8.81 | [-16.46, -1.17] |
| Total | 10-13 | Total | High SES | Depressive disorder | 196 | 171 | 59.08 | 69.60 | 47.32 | 59.86 | .083 | .295 | 0.18 | -11.77 | [-25.15, 1.62] |
| Total | 14-17 | Total | Low SES | Depressive disorder | 302 | 235 | 46.02 | 61.10 | 43.02 | 46.04 | .517 | .795 | 0.05 | -3.00 | [-12.38, 6.39] |
| Total | 14-17 | Total | Medium SES | Depressive disorder | 1,419 | 1,045 | 53.60 | 63.70 | 48.99 | 56.78 | .059 | .241 | 0.08 | -4.61 | [-9.47, 0.26] |
| Total | 14-17 | Total | High SES | Depressive disorder | 518 | 409 | 58.81 | 67.51 | 47.78 | 58.57 | .008 | .072 | 0.17 | -11.03 | [-19.29, -2.77] |
| Female | 0-9 | Total | Total | OCD | 9 | 14 | 79.44 | 68.65 | 23.93 | 31.98 | .046 | .212 | 1.13 | -55.52 | [-96.78, -14.25] |
| Female | 10-13 | Total | Total | OCD | 35 | 19 | 38.09 | 54.70 | 42.21 | 36.80 | .743 | .898 | 0.08 | 4.12 | [-23.38, 31.63] |
| Female | 14-17 | Total | Total | OCD | 40 | 44 | 52.90 | 55.47 | 68.86 | 55.41 | .191 | .432 | 0.29 | 15.96 | [-7.78, 39.70] |
| Male | 0-9 | Total | Total | OCD | 21 | 17 | 36.76 | 63.35 | 37.65 | 60.88 | .965 | .975 | 0.01 | 0.89 | [-38.93, 40.70] |
| Male | 10-13 | Total | Total | OCD | 35 | 12 | 49.23 | 48.39 | 52.92 | 44.25 | .810 | .910 | 0.08 | 3.69 | [-27.40, 34.77] |
| Male | 14-17 | Total | Total | OCD | 27 | 31 | 60.89 | 49.80 | 69.32 | 74.02 | .609 | .850 | 0.13 | 8.43 | [-24.55, 41.42] |
| Female | Total | Urban | Total | OCD | 57 | 52 | 53.33 | 59.29 | 50.60 | 50.00 | .794 | .898 | 0.05 | -2.74 | [-23.43, 17.96] |
| Male | Total | Urban | Total | OCD | 54 | 42 | 46.50 | 46.37 | 66.55 | 73.41 | .127 | .346 | 0.34 | 20.05 | [-4.02, 44.12] |
| Female | Total | Rural | Total | OCD | 27 | 25 | 41.63 | 53.04 | 61.44 | 52.14 | .181 | .431 | 0.38 | 19.81 | [-8.81, 48.43] |
| Male | Total | Rural | Total | OCD | 29 | 18 | 56.14 | 64.23 | 34.94 | 36.74 | .158 | .395 | 0.38 | -21.19 | [-53.82, 11.43] |
| Female | Total | Total | Low SES | OCD | 10 | 12 | 55.50 | 54.43 | 39.17 | 51.20 | .481 | .775 | 0.31 | -16.33 | [-60.54, 27.88] |
| Male | Total | Total | Low SES | OCD | 8 | 12 | 55.75 | 65.55 | 51.92 | 79.62 | .908 | .935 | 0.05 | -3.83 | [-70.45, 62.79] |
| Female | Total | Total | Medium SES | OCD | 58 | 47 | 42.45 | 55.13 | 53.51 | 40.49 | .239 | .498 | 0.23 | 11.06 | [-7.84, 29.96] |
| Male | Total | Total | Medium SES | OCD | 55 | 32 | 45.15 | 47.93 | 59.31 | 63.82 | .281 | .564 | 0.26 | 14.17 | [-9.48, 37.81] |
| Female | Total | Total | High SES | OCD | 16 | 18 | 71.69 | 64.04 | 65.67 | 70.87 | .796 | .898 | 0.09 | -6.02 | [-51.65, 39.61] |
| Male | Total | Total | High SES | OCD | 20 | 16 | 60.50 | 62.14 | 56.44 | 63.23 | .848 | .925 | 0.06 | -4.06 | [-45.23, 37.11] |
| Total | 0-9 | Total | Low SES | OCD | 5 | 6 | 4.60 | 3.13 | 19.67 | 38.94 | .388 | .664 | 0.52 | 15.07 | [-19.47, 49.60] |
| Total | 0-9 | Total | Medium SES | OCD | 18 | 14 | 53.00 | 60.05 | 33.93 | 39.69 | .290 | .572 | 0.37 | -19.07 | [-55.54, 17.39] |
| Total | 0-9 | Total | High SES | OCD | 7 | 11 | 72.86 | 94.85 | 34.73 | 67.04 | .378 | .656 | 0.48 | -38.13 | [-112.64, 36.38] |
| Total | 10-13 | Total | Medium SES | OCD | 53 | 20 | 37.70 | 52.16 | 50.30 | 39.91 | .277 | .561 | 0.26 | 12.60 | [-12.69, 37.90] |
| Total | 10-13 | Total | High SES | OCD | 12 | 8 | 51.67 | 39.10 | 43.38 | 41.26 | .660 | .889 | 0.21 | -8.29 | [-44.04, 27.45] |
| Total | 14-17 | Total | Low SES | OCD | 8 | 15 | 67.50 | 59.18 | 59.40 | 76.05 | .781 | .898 | 0.11 | -8.10 | [-68.92, 52.72] |
| Total | 14-17 | Total | Medium SES | OCD | 42 | 45 | 47.45 | 47.02 | 65.16 | 56.47 | .115 | .332 | 0.34 | 17.70 | [-4.22, 39.62] |
| Total | 14-17 | Total | High SES | OCD | 17 | 15 | 72.18 | 62.13 | 90.40 | 68.75 | .440 | .726 | 0.28 | 18.22 | [-27.12, 63.57] |
| Female | 0-9 | Total | Total | PTSD | 135 | 57 | 17.25 | 45.13 | 31.61 | 63.14 | .123 | .340 | 0.28 | 14.36 | [-1.46, 30.18] |
| Female | 10-13 | Total | Total | PTSD | 121 | 72 | 43.21 | 63.79 | 36.01 | 53.94 | .404 | .674 | 0.12 | -7.20 | [-24.80, 10.40] |
| Female | 14-17 | Total | Total | PTSD | 168 | 147 | 40.96 | 62.25 | 43.15 | 67.07 | .766 | .898 | 0.03 | 2.19 | [-12.10, 16.47] |
| Male | 0-9 | Total | Total | PTSD | 141 | 84 | 33.73 | 64.71 | 38.65 | 65.44 | .584 | .838 | 0.08 | 4.92 | [-12.63, 22.48] |
| Male | 10-13 | Total | Total | PTSD | 77 | 84 | 32.19 | 40.10 | 33.04 | 54.52 | .910 | .935 | 0.02 | 0.85 | [-14.05, 15.74] |
| Male | 14-17 | Total | Total | PTSD | 68 | 47 | 26.54 | 36.51 | 32.62 | 74.87 | .608 | .850 | 0.11 | 6.07 | [-14.54, 26.68] |
| Female | Total | Urban | Total | PTSD | 267 | 191 | 34.87 | 60.75 | 33.54 | 48.97 | .795 | .898 | 0.02 | -1.33 | [-11.76, 9.10] |
| Male | Total | Urban | Total | PTSD | 177 | 122 | 34.40 | 60.55 | 40.26 | 74.40 | .472 | .771 | 0.09 | 5.86 | [-9.49, 21.21] |
| Female | Total | Rural | Total | PTSD | 157 | 85 | 32.67 | 55.60 | 50.96 | 85.87 | .079 | .290 | 0.27 | 18.30 | [0.41, 36.18] |
| Male | Total | Rural | Total | PTSD | 109 | 55 | 27.07 | 37.41 | 25.24 | 36.05 | .762 | .898 | 0.05 | -1.84 | [-13.82, 10.15] |
| Female | Total | Total | Low SES | PTSD | 91 | 54 | 34.27 | 46.83 | 37.19 | 67.93 | .782 | .898 | 0.05 | 2.91 | [-15.81, 21.63] |
| Male | Total | Total | Low SES | PTSD | 55 | 41 | 27.11 | 40.57 | 27.44 | 35.00 | .966 | .975 | 0.01 | 0.33 | [-15.16, 15.82] |
| Female | Total | Total | Medium SES | PTSD | 250 | 174 | 32.30 | 55.73 | 41.60 | 60.60 | .109 | .332 | 0.16 | 9.31 | [-1.87, 20.49] |
| Male | Total | Total | Medium SES | PTSD | 174 | 114 | 30.67 | 48.56 | 35.56 | 61.42 | .475 | .771 | 0.09 | 4.89 | [-7.87, 17.64] |
| Female | Total | Total | High SES | PTSD | 83 | 48 | 39.12 | 77.37 | 31.06 | 66.63 | .531 | .795 | 0.11 | -8.06 | [-34.23, 18.11] |
| Male | Total | Total | High SES | PTSD | 57 | 22 | 38.81 | 73.08 | 50.95 | 112.41 | .642 | .880 | 0.14 | 12.15 | [-29.97, 54.27] |
| Total | 0-9 | Total | Low SES | PTSD | 57 | 32 | 28.67 | 42.68 | 31.31 | 62.50 | .832 | .921 | 0.05 | 2.65 | [-19.28, 24.57] |
| Total | 0-9 | Total | Medium SES | PTSD | 151 | 89 | 19.21 | 47.10 | 35.49 | 55.22 | .021 | .134 | 0.32 | 16.29 | [3.13, 29.45] |
| Total | 0-9 | Total | High SES | PTSD | 68 | 20 | 37.51 | 79.80 | 44.40 | 99.70 | .779 | .898 | 0.08 | 6.89 | [-35.30, 49.07] |
| Total | 10-13 | Total | Low SES | PTSD | 37 | 30 | 31.81 | 38.15 | 23.60 | 26.39 | .303 | .578 | 0.25 | -8.21 | [-24.30, 7.88] |
| Total | 10-13 | Total | Medium SES | PTSD | 130 | 68 | 40.96 | 55.91 | 39.90 | 55.40 | .898 | .935 | 0.02 | -1.06 | [-17.41, 15.29] |
| Total | 10-13 | Total | High SES | PTSD | 31 | 20 | 38.90 | 72.60 | 34.60 | 75.81 | .842 | .922 | 0.06 | -4.30 | [-45.82, 37.22] |
| Total | 14-17 | Total | Low SES | PTSD | 52 | 33 | 34.60 | 51.00 | 43.12 | 68.10 | .539 | .797 | 0.15 | 8.53 | [-16.86, 33.91] |
| Total | 14-17 | Total | Medium SES | PTSD | 143 | 131 | 36.27 | 53.60 | 41.38 | 67.27 | .489 | .775 | 0.08 | 5.12 | [-9.23, 19.46] |
| Total | 14-17 | Total | High SES | PTSD | 41 | 30 | 41.51 | 71.54 | 34.40 | 78.89 | .698 | .898 | 0.10 | -7.11 | [-42.30, 28.07] |
| Female | 0-9 | Total | Low SES | Anxiety disorder | 148 | 96 | 19.09 | 30.72 | 23.22 | 33.83 | .335 | .610 | 0.13 | 4.13 | [-4.08, 12.34] |
| Male | 0-9 | Total | Low SES | Anxiety disorder | 245 | 121 | 29.86 | 41.56 | 31.43 | 38.94 | .723 | .898 | 0.04 | 1.57 | [-7.30, 10.44] |
| Female | 0-9 | Total | Medium SES | Anxiety disorder | 626 | 331 | 23.63 | 44.78 | 18.93 | 30.90 | .057 | .241 | 0.12 | -4.70 | [-10.10, 0.70] |
| Male | 0-9 | Total | Medium SES | Anxiety disorder | 949 | 546 | 27.86 | 42.51 | 25.04 | 37.66 | .185 | .431 | 0.07 | -2.82 | [-7.11, 1.48] |
| Female | 0-9 | Total | High SES | Anxiety disorder | 214 | 129 | 14.21 | 27.64 | 13.05 | 23.40 | .676 | .898 | 0.04 | -1.17 | [-6.88, 4.54] |
| Male | 0-9 | Total | High SES | Anxiety disorder | 323 | 214 | 24.84 | 44.76 | 21.88 | 37.18 | .406 | .674 | 0.07 | -2.96 | [-10.20, 4.28] |
| Female | 10-13 | Total | Low SES | Anxiety disorder | 118 | 113 | 28.48 | 39.50 | 37.28 | 52.64 | .154 | .391 | 0.19 | 8.80 | [-3.17, 20.77] |
| Male | 10-13 | Total | Low SES | Anxiety disorder | 142 | 79 | 40.11 | 43.83 | 27.61 | 35.74 | .023 | .140 | 0.30 | -12.50 | [-23.81, -1.18] |
| Female | 10-13 | Total | Medium SES | Anxiety disorder | 547 | 396 | 43.80 | 58.62 | 34.09 | 44.61 | .004 | .057 | 0.18 | -9.71 | [-16.59, -2.84] |
| Male | 10-13 | Total | Medium SES | Anxiety disorder | 493 | 337 | 39.01 | 49.77 | 36.79 | 42.86 | .493 | .775 | 0.05 | -2.22 | [-8.74, 4.30] |
| Female | 10-13 | Total | High SES | Anxiety disorder | 175 | 98 | 45.98 | 63.68 | 32.43 | 45.19 | .042 | .201 | 0.23 | -13.55 | [-27.83, 0.73] |
| Male | 10-13 | Total | High SES | Anxiety disorder | 132 | 86 | 44.09 | 49.33 | 42.51 | 52.01 | .823 | .919 | 0.03 | -1.58 | [-15.27, 12.11] |
| Female | 14-17 | Total | Low SES | Anxiety disorder | 131 | 110 | 38.09 | 54.73 | 32.20 | 38.41 | .329 | .608 | 0.12 | -5.89 | [-18.05, 6.27] |
| Male | 14-17 | Total | Low SES | Anxiety disorder | 68 | 54 | 19.81 | 28.00 | 31.96 | 39.48 | .059 | .241 | 0.36 | 12.15 | [0.17, 24.14] |
| Female | 14-17 | Total | Medium SES | Anxiety disorder | 502 | 439 | 39.74 | 51.30 | 38.96 | 57.10 | .827 | .919 | 0.01 | -0.78 | [-7.70, 6.15] |
| Male | 14-17 | Total | Medium SES | Anxiety disorder | 254 | 212 | 42.71 | 55.10 | 29.71 | 43.75 | .005 | .058 | 0.26 | -13.00 | [-22.16, -3.83] |
| Female | 14-17 | Total | High SES | Anxiety disorder | 156 | 107 | 38.04 | 57.23 | 35.91 | 47.33 | .742 | .898 | 0.04 | -2.14 | [-15.28, 11.01] |
| Male | 14-17 | Total | High SES | Anxiety disorder | 86 | 63 | 43.38 | 67.42 | 26.54 | 35.92 | .051 | .226 | 0.30 | -16.84 | [-35.15, 1.46] |
| Female | 0-9 | Total | Medium SES | Depressive disorder | 31 | 15 | 24.94 | 38.49 | 26.67 | 33.79 | .877 | .935 | 0.05 | 1.73 | [-21.11, 24.57] |
| Male | 0-9 | Total | Medium SES | Depressive disorder | 58 | 25 | 53.62 | 58.69 | 31.68 | 39.41 | .051 | .226 | 0.41 | -21.94 | [-47.13, 3.24] |
| Female | 0-9 | Total | High SES | Depressive disorder | 6 | 6 | 23.33 | 46.94 | 40.83 | 54.98 | .568 | .823 | 0.34 | 17.50 | [-40.35, 75.35] |
| Male | 0-9 | Total | High SES | Depressive disorder | 20 | 11 | 61.20 | 64.13 | 70.55 | 66.69 | .709 | .898 | 0.14 | 9.35 | [-38.50, 57.19] |
| Female | 10-13 | Total | Low SES | Depressive disorder | 92 | 68 | 53.33 | 54.81 | 41.84 | 49.07 | .166 | .402 | 0.22 | -11.49 | [-27.93, 4.95] |
| Male | 10-13 | Total | Low SES | Depressive disorder | 42 | 18 | 44.48 | 57.95 | 37.89 | 50.20 | .660 | .889 | 0.12 | -6.59 | [-37.39, 24.22] |
| Female | 10-13 | Total | Medium SES | Depressive disorder | 388 | 338 | 55.80 | 70.17 | 47.07 | 55.26 | .061 | .247 | 0.14 | -8.73 | [-18.02, 0.55] |
| Male | 10-13 | Total | Medium SES | Depressive disorder | 168 | 92 | 51.10 | 56.19 | 40.04 | 41.60 | .073 | .278 | 0.21 | -11.05 | [-24.15, 2.04] |
| Female | 10-13 | Total | High SES | Depressive disorder | 136 | 134 | 64.10 | 75.61 | 50.01 | 64.07 | .100 | .323 | 0.20 | -14.09 | [-30.82, 2.64] |
| Male | 10-13 | Total | High SES | Depressive disorder | 60 | 37 | 47.70 | 52.37 | 37.54 | 40.39 | .287 | .571 | 0.21 | -10.16 | [-29.90, 9.58] |
| Female | 14-17 | Total | Low SES | Depressive disorder | 206 | 178 | 47.04 | 63.13 | 43.44 | 47.29 | .524 | .795 | 0.06 | -3.60 | [-14.90, 7.70] |
| Male | 14-17 | Total | Low SES | Depressive disorder | 96 | 57 | 43.82 | 56.75 | 41.72 | 42.28 | .794 | .898 | 0.04 | -2.10 | [-19.10, 14.89] |
| Female | 14-17 | Total | Medium SES | Depressive disorder | 998 | 792 | 58.78 | 69.70 | 51.32 | 58.62 | .014 | .103 | 0.11 | -7.47 | [-13.53, -1.40] |
| Male | 14-17 | Total | Medium SES | Depressive disorder | 421 | 253 | 41.31 | 44.15 | 41.72 | 50.02 | .915 | .935 | 0.01 | 0.41 | [-6.83, 7.65] |
| Female | 14-17 | Total | High SES | Depressive disorder | 367 | 307 | 61.65 | 72.48 | 52.56 | 62.14 | .080 | .291 | 0.13 | -9.09 | [-19.40, 1.21] |
| Male | 14-17 | Total | High SES | Depressive disorder | 151 | 102 | 51.89 | 53.13 | 33.39 | 43.38 | .003 | .050 | 0.37 | -18.50 | [-30.91, -6.08] |
| Female | 0-9 | Total | Medium SES | OCD | 5 | 8 | 109.00 | 57.89 | 24.38 | 29.49 | .029 | .157 | 2.01 | -84.62 | [-131.66, -37.59] |
| Male | 0-9 | Total | Medium SES | OCD | 13 | 6 | 31.46 | 46.71 | 46.67 | 50.37 | .547 | .803 | 0.32 | 15.21 | [-31.05, 61.46] |
| Female | 10-13 | Total | Medium SES | OCD | 27 | 14 | 29.56 | 53.31 | 52.07 | 35.98 | .118 | .334 | 0.47 | 22.52 | [-8.62, 53.65] |
| Male | 10-13 | Total | Medium SES | OCD | 26 | 6 | 46.15 | 50.56 | 46.17 | 51.56 | 1.00 | 1.00 | 0.00 | 0.01 | [-45.02, 45.04] |
| Male | 10-13 | Total | High SES | OCD | 8 | 5 | 53.62 | 43.59 | 56.80 | 43.18 | .901 | .935 | 0.07 | 3.17 | [-45.36, 51.71] |
| Female | 14-17 | Total | Low SES | OCD | 5 | 8 | 41.60 | 39.83 | 44.50 | 55.45 | .915 | .935 | 0.06 | 2.90 | [-53.34, 59.14] |
| Female | 14-17 | Total | Medium SES | OCD | 26 | 25 | 43.04 | 48.69 | 63.64 | 42.37 | .113 | .332 | 0.45 | 20.60 | [-4.49, 45.69] |
| Male | 14-17 | Total | Medium SES | OCD | 16 | 20 | 54.62 | 44.76 | 67.05 | 71.49 | .529 | .795 | 0.20 | 12.42 | [-27.78, 52.63] |
| Female | 14-17 | Total | High SES | OCD | 9 | 11 | 87.67 | 71.02 | 98.45 | 72.35 | .742 | .898 | 0.15 | 10.79 | [-52.43, 74.01] |
| Female | 0-9 | Total | Low SES | PTSD | 26 | 14 | 26.38 | 35.08 | 47.64 | 88.53 | .402 | .674 | 0.36 | 21.26 | [-17.13, 59.65] |
| Male | 0-9 | Total | Low SES | PTSD | 31 | 18 | 30.58 | 48.65 | 18.61 | 27.18 | .275 | .561 | 0.28 | -11.97 | [-36.46, 12.52] |
| Female | 0-9 | Total | Medium SES | PTSD | 72 | 34 | 8.22 | 13.33 | 29.50 | 57.64 | .041 | .199 | 0.62 | 21.28 | [7.29, 35.26] |
| Male | 0-9 | Total | Medium SES | PTSD | 79 | 55 | 29.22 | 62.39 | 39.20 | 53.86 | .325 | .605 | 0.17 | 9.98 | [-10.34, 30.31] |
| Female | 0-9 | Total | High SES | PTSD | 37 | 9 | 28.41 | 77.62 | 14.67 | 25.88 | .378 | .656 | 0.19 | -13.74 | [-65.51, 38.03] |
| Male | 0-9 | Total | High SES | PTSD | 31 | 11 | 48.39 | 82.28 | 68.73 | 130.02 | .636 | .877 | 0.21 | 20.34 | [-46.01, 86.69] |
| Female | 10-13 | Total | Low SES | PTSD | 24 | 17 | 35.83 | 42.74 | 20.00 | 24.07 | .140 | .377 | 0.44 | -15.83 | [-38.36, 6.70] |
| Male | 10-13 | Total | Low SES | PTSD | 13 | 13 | 24.38 | 27.82 | 28.31 | 29.46 | .730 | .898 | 0.14 | 3.92 | [-18.10, 25.95] |
| Female | 10-13 | Total | Medium SES | PTSD | 78 | 39 | 44.21 | 65.05 | 48.56 | 64.90 | .733 | .898 | 0.07 | 4.36 | [-20.63, 29.34] |
| Male | 10-13 | Total | Medium SES | PTSD | 52 | 29 | 36.10 | 38.45 | 28.24 | 37.20 | .372 | .656 | 0.21 | -7.85 | [-25.12, 9.41] |
| Female | 10-13 | Total | High SES | PTSD | 19 | 16 | 48.47 | 81.15 | 22.44 | 39.85 | .228 | .493 | 0.40 | -26.04 | [-69.72, 17.65] |
| Female | 14-17 | Total | Low SES | PTSD | 41 | 23 | 38.37 | 55.28 | 43.52 | 75.66 | .776 | .898 | 0.08 | 5.16 | [-27.15, 37.46] |
| Male | 14-17 | Total | Low SES | PTSD | 11 | 10 | 20.55 | 27.66 | 42.20 | 49.91 | .247 | .509 | 0.54 | 21.65 | [-12.41, 55.72] |
| Female | 14-17 | Total | Medium SES | PTSD | 100 | 101 | 40.34 | 61.61 | 42.99 | 59.88 | .757 | .898 | 0.04 | 2.65 | [-14.15, 19.45] |
| Male | 14-17 | Total | Medium SES | PTSD | 43 | 30 | 26.79 | 25.20 | 35.97 | 88.79 | .586 | .838 | 0.15 | 9.18 | [-18.78, 37.13] |
| Female | 14-17 | Total | High SES | PTSD | 27 | 23 | 47.22 | 75.39 | 43.48 | 88.50 | .874 | .935 | 0.05 | -3.74 | [-49.16, 41.67] |
| Male | 14-17 | Total | High SES | PTSD | 14 | 7 | 30.50 | 64.68 | 4.57 | 3.41 | .159 | .395 | 0.48 | -25.93 | [-74.50, 22.64] |

*Note.* ^1^with at least one inpatient stay in one of the following departments: child and adolescent psychiatry, general psychiatry, psychosomatics/psychotherapy or pediatrics; Cohen's |d|: small effect: ≥0.2, medium effect: ≥0.5, large effect: ≥0.8; n, sample size; *M*, mean; *SD*, standard deviation; *ΔM*, mean difference (intra-COVID-19 – pre-COVID-19); Δ%; *p.adj*, Benjamini-Hochberg adjusted *p*-values, Benjamini-Hochberg procedure was applied with the following parameters: i = rank of p-value, m = 229 = total number of tests and Q = 0.05 = false discovery rate, using the formula for critical value = (i/m))* Q; only comparisons with *n* >5 are included and only comparisons with *p.adj* < .05 and |d| ≥ 0.2 are highlighted.
