# Supplementary material for: Continuity of care: time to first outpatient appointment after child and adolescent psychiatric hospital stays in Germany
Source: BMC Health Serv Res. 2026 Mar 13;26:486. doi: 10.1186/s12913-026-14322-7 (PMC13063905; doi:10.1186/s12913-026-14322-7)
Supplement: Supplementary file 3 — Supplementary Material 3: After hospitalization, inpatient readmission and emergency room admission before first outpatient contact of female and male patients by disorder and readmission facility. [file 12913_2026_14322_MOESM4_ESM.docx]

**Supplemental Table 3**. After hospitalization, inpatient readmission and emergency room admission before first outpatient contact of female and male patients by disorder and readmission facility

**Female and male patients, all age groups**

|  |  | Pre-COVID | | Intra-COVID | |  |
| --- | --- | --- | --- | --- | --- | --- |
| ICD |  | *n* | *%* | *n* | *%* | *Δ%* |
| Anorexia nervosa |  |  |  |  |  |  |
|  | Child and adolescent psychiatry | 55 | 14.47 | 57 | 14.25 | -1.52 |
|  | General Psychiatry | 6 | 1.58 | <5 | - | - |
|  | Psychosomatics/Psychotherapy | 11 | 2.89 | 5 | 1.25 | -56.75 |
|  | Pediatrics | 30 | 7.89 | 30 | 7.50 | -4.94 |
|  | Emergency room admission | 24 | 6.32 | 23 | 5.75 | -9.02 |
| Anxiety disorder |  |  |  |  |  |  |
|  | Child and adolescent psychiatry | 227 | 4.60 | 122 | 3.71 | -19.35 |
|  | General Psychiatry | 11 | 0.22 | <5 | - | - |
|  | Psychosomatics/Psychotherapy | 5 | 0.10 | <5 | - | - |
|  | Pediatrics | 381 | 7.72 | 275 | 8.35 | +8.16 |
|  | Emergency room admission | 180 | 3.65 | 134 | 4.07 | +11.51 |
| Depressive disorder |  |  |  |  |  |  |
|  | Child and adolescent psychiatry | 283 | 9.32 | 200 | 8.69 | -6.76 |
|  | General Psychiatry | 38 | 1.25 | 12 | 0.52 | -58.40 |
|  | Psychosomatics/Psychotherapy | 17 | 0.56 | 8 | 0.35 | -37.50 |
|  | Pediatrics | 154 | 5.07 | 111 | 4.82 | -4.93 |
|  | Emergency room admission | 203 | 6.69 | 164 | 7.13 | +6.58 |
| OCD |  |  |  |  |  |  |
|  | Child and adolescent psychiatry | 18 | 11.69 | 11 | 8.66 | -25.92 |
|  | General Psychiatry | - | - | <5 | - | - |
|  | Psychosomatics/Psychotherapy | - | - | <5 | - | - |
|  | Pediatrics | 7 | 4.55 | 8 | 6.30 | +38.46 |
|  | Emergency room admission | 9 | 5.84 | 9 | 7.09 | +21.40 |
| PTSD |  |  |  |  |  |  |
|  | Child and adolescent psychiatry | 33 | 4.99 | 23 | 5.53 | +10.82 |
|  | General Psychiatry | <5 | - | <5 | - | - |
|  | Psychosomatics/Psychotherapy | - | - | - | - | - |
|  | Pediatrics | 72 | 10.89 | 41 | 9.86 | -9.46 |
|  | Emergency room admission | 47 | 7.11 | 30 | 7.21 | +1.41 |

*Note.* Base populations for the percentages refer to cases with at least one hospital stay with discharge before the end of the study period and at least one contact with an included specialist groups between the date of discharge and the end of the respective study period; *Δ%*, relative percentual change ((intra-COVID-19 – pre-COVID-19)/pre-COVID-19).
